# ‘Rich’ and ‘poor’ in mentalizing: Do expert mentalizers exist?

**DOI:** 10.1371/journal.pone.0259030

**Published:** 2021-10-25

**Authors:** Simon Rogoff, Alesia Moulton-Perkins, Fiona Warren, Tobias Nolte, Peter Fonagy

**Affiliations:** 1 Department of Psychology, University of Surrey, Guildford, United Kingdom; 2 Anna Freud National Centre for Children & Families, London, United Kingdom; 3 Research Department of Clinical, Educational and Health Psychology, UCL, London, United Kingdom; Medical University of Vienna, AUSTRIA

## Abstract

Mentalization theory is concerned with the capacity to notice, and make sense of, thoughts and feelings in self and others. This development may be healthy or impaired and therefore, by extension, it may be theorized that expertise in mentalizing can exist. Furthermore, a continuum from impairment to expertise should exist within separate dimensions of mentalizing: of self and of others. This study hypothesized that three groups would be distinguishable on the basis of their mentalizing capacities. In a cross-sectional design, Psychological Therapists (‘expert’ mentalizers; *n* = 51), individuals with a diagnosis of Borderline Personality Disorder (‘poor’ mentalizers; *n* = 43) and members of the general population (‘non-clinical controls’; *n* = 35) completed a battery of self-report measures. These assessed the mentalizing of self and of others (using an extended version of the Reflective Function Questionnaire (RFQ18)), alexithymia and cognitive empathy. As hypothesized, Psychological Therapists’ scores were higher than controls on self-mentalizing and control group scores were higher than those with BPD. Cognitive empathy scores in the BPD group indicated markedly lower capacities than the other two groups. Contrary to predictions, no significant differences were found between groups on mentalizing others in RFQ18 scores. The Psychological Therapist and BPD profiles were characterized by differential impairment with regards to mentalizing self and others but in opposing directions. Results suggest that the RFQ18 can identify groups with expertise in mentalizing. Implications of these results for the effectiveness of psychological therapy and of Psychological Therapists are discussed.

## Introduction

Mentalizing capacity, or reflective function (RF), is an aspect of personality functioning described as ‘the capacity to make sense of self and of others in terms of subjective mental states and mental processes’ [1, p83]. Bateman and Fonagy [2] suggest distinct dimensions of mentalizing which include mentalizing self, mentalizing others and cognitive vs affective mentalizing. The construct of empathy overlaps with mentalization of others whilst alexithymia is closely related to self-mentalizing [3].

Mentalization theory was developed to explain the particular profile of deficits observed in Borderline Personality Disorder (BPD) [4] which has been shown to be characterised by difficulties mentalizing self and others [5–7]. Part of the emerging evidence is that those with BPD have a heightened sensitivity (affective empathy) specifically to the emotions of others [8–10]. However, this sensitivity to distress in the other can have a ‘contagion effect’ leading to the unmoderated emotional arousal in the self [11, 12]. Concurrently, an individual with BPD may have a propensity to ‘hyper-mentalize’ about the mind of the other (cognitive empathy) to the extent that they generate coherent yet over-confident accounts of the other’s perspective [13, 14]. The resulting complex profile of mentalizing capacity has been termed the ‘double dissociation’ hypothesis [11]; combining impaired self-mentalizing with some proficiency in mentalizing others’ feelings coupled with impairment in relation to others’ thoughts.

As mentalizing impairment becomes increasingly acknowledged as a target for treatment of BPD, understanding both the variance in this capacity and its multidimensional structure become more important. If there is proficiency on one dimension for an individual then impairment on others might still be a necessary focus for treatment [15, p196].

Understood as a normal developmental achievement, mentalizing should offer the potential for expertise in addition to impairment [16]. Allen et al. [15], reviewing the literature state that ‘super-mentalizers’ might be found amongst ‘mind-minded mothers’ [17] and psychotherapists [15]. ‘Super-mentalizing’ however, should not be confused with a ‘hyperactive’ style in which reflections do not lead to increased understanding [18]. Instead, proficient mentalizing must contain both a capacity for nuanced observation of mental states but also the realistic appreciation of their opacity. No published studies to date have investigated ‘expert’ mentalizers explicitly.

As the understanding of deficits in mentalizing self and other*s* in BPD is elaborated, it becomes clear that these capacities are also specific to the expertise of Psychological Therapists. Functions of healthy mentalizing include the capacity to empathise [19], to think rationally in the face of distress [5] and to sustain psychologically intimate relationships [20, 21]. Mentalization might be a helpful framework within which to observe, measure and describe such personality variables in the selection, training and supervision of Psychological Therapists.

### Selecting for a particular profile of mentalizing capacity in Psychological Therapists

It has been demonstrated that some professional groups are characterised by a high prevalence of particular personality traits [22], some of which may reflect mentalizing capacity. With regard to Psychological Therapists however, the literature contains an array of conflicting hypotheses about their mentalizing profile. From this literature a number of factors are implicated; both intra- and extra-personal, which might select for a particular profile of mentalizing in this group.

There is consensus that the profession of Psychological Therapy is indeed a chosen one [23–25] in which personal need satisfaction is a major factor [26]. Whilst motivation might predict enhanced reflective capacity, a now substantial body of research suggests that Psychological Therapists report a higher prevalence of childhood trauma and emotional neglect when compared to other professional groups [26–29]. Such experiences are described by Mentalization theory as predicting deficits in adulthood [2].

An ‘impaired mentalizers’ hypothesis suggests that Psychological Therapists may be motivated by types of past trauma associated with impairment in mentalizing [2, 30]. Some have suggested an impact upon self-mentalizing specifically paired with a preoccupied mentalizing of others (initially of caregivers) [25, 31]. These arguments are at odds with Allen and colleagues’ [15] theory about the centrality of mentalizing in effective psychotherapy. However, there has been little empirical support for such arguments. According to an ‘expert mentalizers’ hypothesis, psychotherapist helpfulness may stem from a process of repair following adverse childhood events [26, 30, 32–34]. Evidence cited for this has tended to imply more about enhanced mentalizing of others than of self [e.g., 35]. An ‘adverse enough’ quality of early emotional neglect or trauma might promote the development of active mentalizing of others accompanied by a relative neglect of self [31]. Some recent studies measuring reflective functioning in therapists have suggested that RF may transform attachment anxiety from a negative to a positive factor in therapist effectiveness; capitalizing on, rather than being impaired by, early adversity [30, 36]. With ‘impaired mentalizers’ arguments referring most often to self-mentalizing and ‘expert mentalizers’ arguments referring most often to empathy (operationalized here as mentalizing others), these are not mutually exclusive.

A second factor influencing membership of this group is selection. Selection criteria for Clinical Psychologists exclude a relatively high number of applicants [24, 37] and include the ability to empathise with a wide range of clients and to self-reflect [38], as assessed by supervisors and training entry criteria [24]. The selection process for Counselling Psychology training is based on similar criteria to Clinical Psychology [39] and requirements for qualification as a psychotherapist and psychoanalyst similarly rely on endorsement of personal attributes [40]. Greason and Cashwell [41] argue that training programs may select for empathic capacities, at the cost of skills related to self-mentalizing.

In terms of role performance, empathy and reflective function are viewed as fundamental aspects of the psychotherapeutic process [42] which correlate with outcomes [30, 43, 44]. Not only are the demands on emotional regulation high, but these operations must be performed strategically [45]. Using the Reflective Function Questionnaire (RFQ8) [5], Brugnera et al. [46] showed that Mentalizing capacity specifically mediates the impact of attachment insecurity in therapists on their perceived wellbeing. Cologon et al. [30] measured RF in 25 therapists and estimated that across 1001 clients RF accounted for 70.5% of variance in therapist effectiveness.

Finally, exposure to training and the role of Psychological Therapists may nurture the ability to tolerate difficult emotions, ambiguity and confusion [47] whilst helping to develop advanced metacognitive skills allied to expert mentalizing [45]. Personal therapy; a requirement for many psychodynamic therapists, is also aimed at nurturing mentalizing skills [29, 36, 48].

### The measured mentalizing capacities of Psychological Therapists

Whitman and Bloch [49, p1] describe a perception held by idealizing patients and public alike that might correspond to expert mentalizers:

‘Therapists are considered to have wonderful marriages, impeccable children, total balance in their emotional lives, outstanding work relationships…’

Some researchers have attempted to measure capacities allied to mentalizing, such as empathy, in Psychological Therapists. Hall et al. [42] found that an orientation towards psychotherapy (or teaching or advising) was significantly associated with higher dispositional empathy than was orientation towards research and testing.

In summary, various selecting factors may act upon the Psychological Therapy profession resulting in a particular profile of mentalizing capacity. Given the poverty of robust research to date, any hypothesis concerning the proficiency or impairment in mentalizing of self vs others in Psychological Therapists must be cautious. However, more empirical evidence appears to support arguments for enhanced mentalizing of others in this group.

### Hypotheses

Between groups, we hypothesized that with regard to both mentalizing self and others, Psychological Therapists would score higher than controls. We hypothesized that controls would in turn score higher than BPD participants with regard to both mentalizing self and others.

Within groups, we hypothesized that relative to the control group, Psychological Therapists would be characterised more by enhanced mentalization of others than by enhanced mentalization of self. Hence, we predicted that there would be a significant difference between self and other mean scores with reference to the control group in this direction.

In the BPD group, based on the ‘double dissociation’ hypothesis alone, we might expect a profile containing enhanced mentalizing of others’ emotions. However, there is also evidence that mentalizing globally is impaired in this group and enhanced empathy has not been consistently detected in past research. Furthermore, emotional and cognitive empathy are conflated in the ‘other’ subscale of the RFQ18. Hence, we hypothesized that the RFQ18 would not detect a significant difference between self and other mean scores with reference to the control group in BPD.

## Methods

### Design

A cross-sectional questionnaire-based design was used in this study. Two dimensions of mentalizing ability were measured in three samples: an ‘expert’ mentalizing group (Psychological Therapists), a ‘poor’ mentalizing group (individuals in treatment for BPD) and a control group (an opportunity sample recruited from the general public in London, UK).

### Participants, settings and ethical considerations

Psychological Therapists were qualified and trainee Clinical or Counselling Psychologists, and qualified Psychoanalysts. Following Hall et al. [42], those primarily interested in psychometric testing, research or management were excluded. Those working in personality disorder services were excluded to reduce any potential confound of attachment styles in those attracted to this client group [50]. Those with a specialist interest in mentalization were also excluded. Trainees were recruited from two Clinical Psychology courses and one Counselling Psychology course at two Universities in South East England, by email. Using a short presentation, Qualified Psychoanalysts were recruited from a monthly supervision group and qualified Clinical Psychologists were recruited from two regional forums for Clinical Psychologists.

BPD group participants were recruited from 15 specialist UK NHS BPD services in London (4), Surrey (10) and Lancashire (1). Individuals were approached by service-based clinicians at assessment or at the end of an introductory psycho-education group. Inclusion criteria was a diagnosis of BPD as assessed by the specialist service, having received no more than five weeks of MBT or DBT therapy sessions, and met the threshold for BPD on the Personality Assessment Inventory [51] screener. Specialist services used a range of structured diagnostic assessment tools (see Measures).

Non-clinical controls were recruited from 10 London cafes. Questionnaire packs were left displayed in those areas used to display posters and information. Psychological Therapists and those meeting a threshold for BPD were excluded.

Power calculations were conducted using G*Power [52], based on the effect size of *r* = .31 reported for the RFQ46 [53] as no such figures were available for the RFQ18 at the start of the study. These suggested a total sample size of 105 was needed to test two-tailed hypotheses using ANOVA. In 2010–11 599 adults aged 18–69 were invited to take part in the study; 129 providing data. Of these, 51 (41% response rate) were Psychological Therapists, 35 (27%) were non-clinical controls and 43 (45%) were BPD (S1 Fig).

Ethical approval regarding procedures and materials was gained from the South East Research Ethics Committee, Kent, UK and from five UK National Health Service Trusts involved. Participants gave written consent and completed a battery of self-report questionnaires. These formed parts of a larger battery administered to participants as part of a wider research project [5] (S3 Text).

### Measures

#### Mentalizing capacity—Self and others

The Reflective Function Questionnaire (RFQ) is a questionnaire developed by Fonagy et al. [5]. Questions probe respondents’ capacities in noticing and making sense of mental experiences in self and others (e.g., ‘People’s thoughts are a mystery to me’). During development a 54-item version of the RFQ was factor analysed by Moulton-Perkins et al. [54] (S1 Presentation). On a sub-sample of 100 participants from the validation study they confirmed earlier work by Perkins [53] identifying two factors; ‘mentalizing self’, and ‘mentalizing others’, with nine items loading onto each. Moulton-Perkins et al. [54] found that the resulting 18-item RFQ18 showed good convergent construct validity; correlating positively with measures of mindfulness (*r* = .40, *p* < .001) and cognitive empathy (*r* = .48, p < .001) [54]. Divergent construct validity was supported in negative correlations with measures of Alexithymia (*r* = -.37, p < .001), Borderline pathology (*r* = -.54, p < .001) and general psychopathology (*r* = -0.51, *p* < .001) [54]. Internal reliability of both 9-item subscales was also adequate (Internal-Self: *α* = .75; Internal-Other: *α* = .76) [54]. RFQ54 global scores differentiate between groups who self-harm or display disordered eating and non-clinical controls [55].

Subsequently, the RFQ54 was developed further and a shortened version containing 8 items from the RFQ54 was published [5]. The RFQ8 contains two subscales measuring ‘uncertainty’ and ‘certainty’ in mentalizing. Seven of the RFQ8’s items appear in the RFQ18. Here, in order to assess reported self- and other- mentalizing, the RFQ54 was administered and the RFQ18 was extracted for analysis.

#### Alexithymia

The 20-item Toronto Alexithymia Scale (TAS) [56] measures the inability to describe experienced feelings to others [57]. TAS correlates negatively with measures of emotional intelligence and psychological mindedness [57]. The TAS shows good internal consistency (Cronbach’s *α* = .81), test-retest reliability and construct validity [56].

#### Cognitive empathy

The Interpersonal Reactivity Index [58] is a multidimensional measure of empathy of which the seven-item Perspective Taking Subscale (PTS) measures cognitive aspects of empathy; the ‘tendency to spontaneously adopt the psychological point of view of others’ [58]. The PTS has shown good construct validity, predictive criterion validity, divergent construct validity [20, 58] and internal consistency (Cronbach’s *α* = .75) [59].

#### Borderline personality disorder

In the 24-item Borderline Subscale of the Personality Assessment Inventory (PAI-BOR) [51] scores correlate highly with clinician assessment using evidence based structured interview tools [60, 61] with good internal consistency (Cronbach’s *α* = .86) [51]. Jacobo et al. [61] found that a cut-off score of T ≥ 65 optimally differentiated between those who did and did not meet criteria for BPD. All participants in the BPD group had been assessed by a specialist service as having BPD using either the Zanarini Rating Scale for Borderline Personality Disorder [62] or the SCID-II [63].

#### Potential confounds

In order to explore the possibility of group differences in RFQ scores reflecting differences in variables such as intelligence, five potential confounds were measured using additional questionnaires. The Brief Symptom Inventory (BSI18) measured anxiety and depression [64] and the Mill Hill Vocabulary Scale (MHV) measured verbal intelligence [65]. Socio-economic status was measured using three categorical variables regarding employment status, occupation type and years in education. Social-desirability was evaluated using the Impression Management Subscale (IMS) [66]. Participants were also asked about the amount of psychological therapy they had previously received.

### Statistical analyses

All analysis was done using SPSS [67]. The distribution of RFQ-other scores in the BPD group appeared to be bimodal with high and low clusters of scores (S2 Fig). Controls and Psychological Therapist groups were therefore combined and their RFQ-other scores were subjected to frequency analysis in order to establish a cut-off RFQ-other score, below which lay the lowest 10^th^ percentile of scores (based on the estimated prevalence of personality disorder in the general population [68]). This RFQ-other cut-off score of 30 was then used to split the BPD group into high and low RFQ-other scorers [69]. The resulting two groups were compared on BSI12, PAI-BOR, MHV, treatment site and IMS using independent t-tests.

For each dependent variable, if there was a theoretical argument for a potential confound having an effect, parametric tests were performed to test for a significant effect (*p* < .05). If a significant relationship was found between a potential confound and RFQ scores then group differences on the variable were tested for using parametric (ANOVA) or non-parametric (Chi-Square) tests. If a significant (*p* < .05) group effect was not found then the variable was controlled for using ANCOVA [70] (S2 Table).

#### Generating self-other mentalizing profiles

In order to test for differential proficiency in self and other mentalizing within groups with reference to a normative sample, all scores were standardised with reference to control group scores. *Z*-scores were calculated by subtracting the control group mean for the subscale from each score and dividing the result by the control group standard deviation for the subscale [71]. Control group means represent here a threshold below and above which impairment and proficiency are measured.

## Results

### Demographics

The groups were not significantly different with regards to age, ethnicity or verbal intelligence (S1 Table). The percentage of females in the Psychological Therapists sample (97.5%) was significantly higher than in the BPD sample (78.9%) and controls (84.8%) (X^2^(2) = 6.36, *p* = .039, Cramer’s *V* = .24). The percentage of Psychological Therapists in a relationship was greater than that of both control and BPD groups (*X*^2^(2) = 19.21, *p* < 0.001, Cramer’s *V* = .42). Psychological therapists were educated to a higher level than controls and controls educated to a higher level than BPD participants (*X*^2^(10) = 1.27, *p* < .001, Cramer’s *V* = .76). In these same directions, the groups were different in employment status (*X*^2^(4) = 60.93, *p* < .001, Cramer’s *V* = .52) and type of employment (*X*^2^(2) = 45.95, *p* < .001, Cramer’s *V* = .65). The BPD group reported more anxiety and depression than the other two groups (BSI12) (*F*(2,106) = 103.77, *p* < .001).

Age, gender, ethnicity and amount of therapy previously received showed no significant relationship to outcome measures. The absence of any relationship between therapy received and RFQ scores or PTS or TAS scores was surprising. However, the simplicity of the questions measuring therapy received precluded further analysis. Of those potential confounds which were significantly related to RFQ scores, all except Impression Management showed a significant group effect (see S2 Table). See S3 Table containing descriptive statistics for outcome variables across groups.

Opportunities to benchmark our control sample against others were restricted by the limited use of the RFQ18 in previous research. Therefore, for PTS and TAS we searched for adult female non-clinical control samples to compare with our 84.8% female controls. For the PTS, comparing our controls with Guttman and Laportes’ [8], t-test revealed a non-significant difference in means (*t*(58) = 1.21, *p* > 0.05)s. On TAS scores, t-tests revealed significant differences such that our control sample scored lower for alexithymia. Compared with those of Parker et al. [72], the effect size was small (*t*(1084) = 3.75, *p* < .01, *r* = 0.11) and with those of Taylor et al. [73], the effect size was medium (*t*(149) = 3.69, *p* < .01, *r* = 0.29).

#### Total RFQ18 scores across groups

Comparing BPD, Controls and Psychological Therapists on RFQ18 scores, ANOVA Welch’s *F* statistic applied to the combined model showed significant differences of large effect (*F*(2,107) = 24.07, *p* < .001, *r* = .53). Planned comparisons revealed significant differences between BPD (*M* = 59.91, *SD* = 17.80) and both controls, (*M* = 76.64, *SD* = 12.56; *t*(64.73) = -4.61, *p* < .001, *r* = .32), and Psychological Therapists, (*M* = 83.07, *SD* = 10.21; *t*(56.418) = -6.96, *p* < .001, *r* = .68) indicating medium and large effect sizes respectively. A significant difference was found also between controls and Psychological Therapists, (*t*(61.40) = -2.37, *p* = .02, *r* = .29) with a medium effect size.

### Mentalizing self

**Fig 1:**
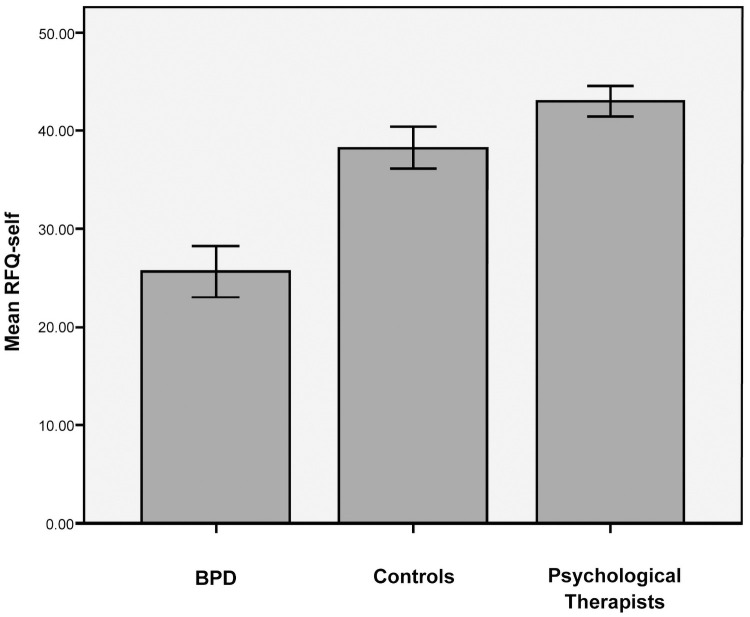
RFQ-self: Comparing means between groups. Error bars: 95% CI.

Comparing BPD, Controls and Psychological Therapists on RFQ-self scores, ANOVA revealed a significant effect of group with large effect size (*F*(2,107) = 75.86, *p* < .001, *r* = .77) (Fig 1). Trend analysis across groups indicated a significant linear relationship between group and RFQ-self, (*F* (1,107) = 151.11, *p* < .001) such that Psychological Therapists (*M* = 42.97, *SD* = 4.87) scored higher than controls (*M* = 38.24, *SD* = 5.98) who scored higher than BPD (*M* = 25.62, *SD* = 7.86) (Fig 1).

Planned contrasts revealed significant differences between Psychological Therapists and both controls (*t*(107) = -3.18, *p* = .002, *r* = .29) and the BPD group (*t*(107) = -12.01, *p* < .001, *r* = .76) with medium and large effect sizes respectively. Borderline Personality Disorder group scores were significantly lower than Controls (*t*(107) = -8.32, *p* < .001, *r* = .63) with a large effect size. Hence, in support of our hypotheses, Psychological Therapists reported self-mentalizing capacities superior to controls and the BPD group whilst the BPD group reported lower self-mentalizing capacities than both controls and Psychological Therapists (Fig 1).

#### Comparing groups on alexithymia

In order to test whether a measure of alexithymia supported the group differences in RFQ-self, TAS scores were subject to a post-hoc one-way ANOVA. Equality of variances could not be assumed and so in ANOVA Welch’s *F* statistic was used. For the combined model the group effect was significant with a large effect size, (*F*(2,67.3) = 103.73, *p* < 0.001, *r* = .84). In planned comparisons however, Psychological Therapist scores (*M* = 36.36, *SD* = 5.96) were not significantly different to controls (*M* = 36.79. *SD* = 7.56). The BPD group (*M* = 61.95, *SD* = 9.73) had significantly higher scores than controls in (*t*(68.20) = -12.24, *p* < .001), and Psychological Therapists (*t*(61.03) = 13.87, *p* < .001). Hence, a superiority of Psychological Therapists on alexithymia over the other two groups, although indicated by RFQ-self scores, was not found.

### Mentalizing others

**Fig 2:**
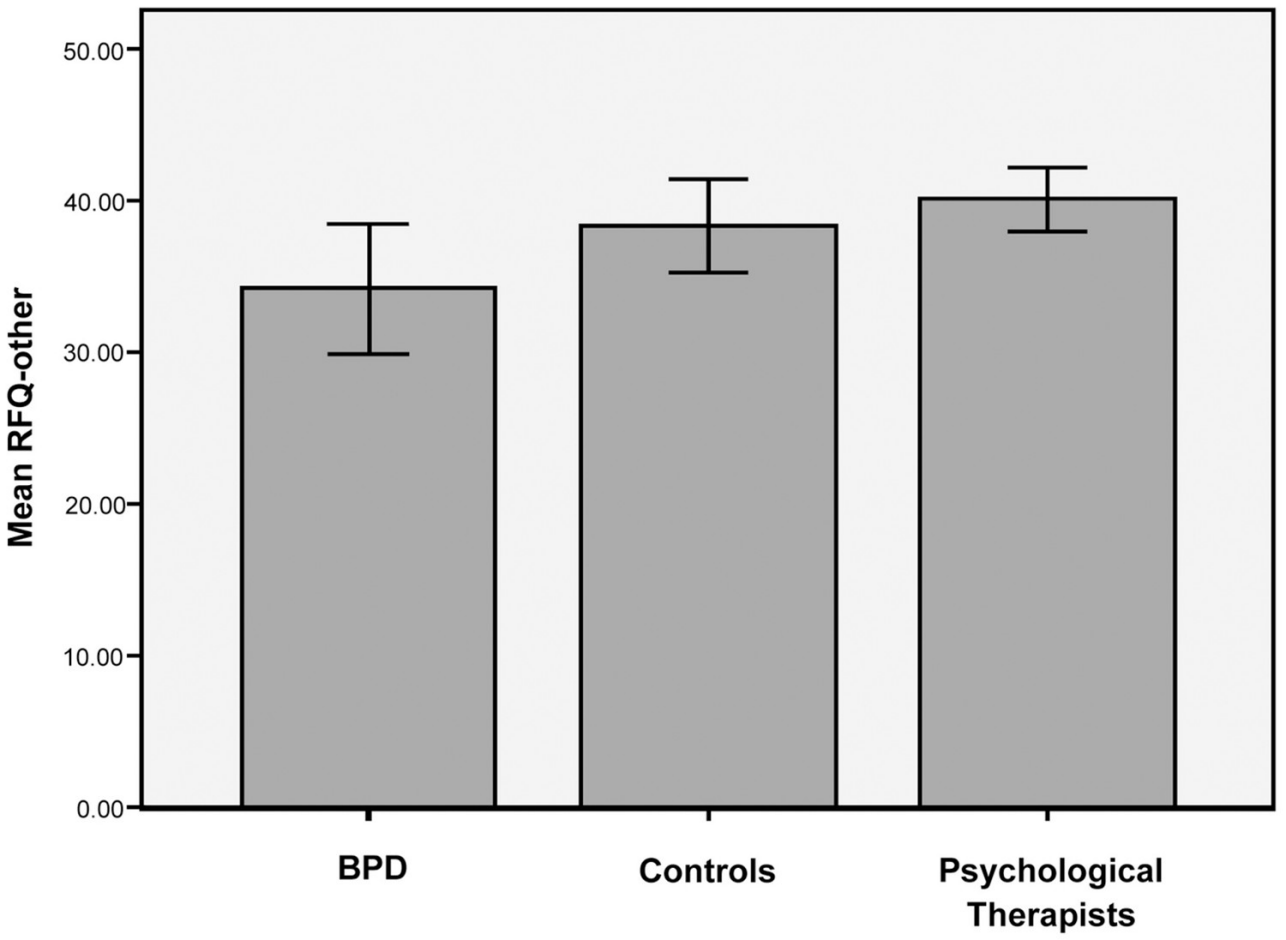
RFQ-other: Comparing means between groups. Error bars: 95% CI.

Comparing BPD, Controls and Psychological Therapists on RFQ-other scores, equality of variances could not be assumed and so in ANOVA Welch’s *F* statistic was used. For the combined model the group effect was significant with a small effect size, (*F*(2,107) = 3.155, *p* = .049, *r* = .07). Trend analysis indicated a significant linear relationship between group and RFQ-other such that Psychological Therapists (*M* = 40.10, *SD* = 6.69) scored higher than controls (*M* = 38.39, *SD* = 8.71) who scored higher than BPD (*M* = 34.19, *SD* = 12.81; *F*(1,107) = 7.43, *p* = .01) (see Fig 2).

Planned comparisons, however, revealed no significant difference on RFQ-other between Psychological Therapists and controls (*t*(59.20) = 0.92, *p* >.05), or between BPD and controls (*t*(63.73) = 1.62, *p* >.05). A significant difference was found only between BPD and Psychological Therapist groups with a medium effect size (*t*(53.31) = -2.51, *p* = .015, *r* = .32). Hence, our hypotheses that Psychological Therapists would score higher than controls who in turn would score higher than the BPD group were not supported.

#### Covariate analysis

Impression management correlated negatively with RFQ-other scores. Controlling for impression management in ANCOVA led to a significant RFQ-other group effect remaining. However, assumptions of homogeneity of variances in ANCOVA were not met. Post hoc pair-wise comparisons with Bonferroni corrections detected no group effect on RFQ-other scores.

#### Comparing groups on cognitive empathy

In order to test whether a measure allied to mentalizing other*s* produced corresponding group differences to the RFQ-other subscale, cognitive empathy was subject to post-hoc one-way ANOVA comparing groups.

Comparing BPD, Controls and Psychological Therapists on PTS(SQRT) scores, ANOVA revealed a significant group effect on cognitive empathy (F(2,107) = 19.75, *p* = .000, *r* = .27) with a small effect size. Planned comparisons revealed a significant difference between BPD and controls (*t*(107) = 5.06, *p* < .001, *r* = .44), and between BPD and Psychological Therapist groups, (*t*(107) = 5.72, *p* < .001, *r* = .48) both with a medium effect size. Hence in cognitive empathy the BPD group scored significantly lower than both controls and Psychological Therapists with medium to large effect sizes.

### Generating group mentalizing profiles

**Fig 3:**
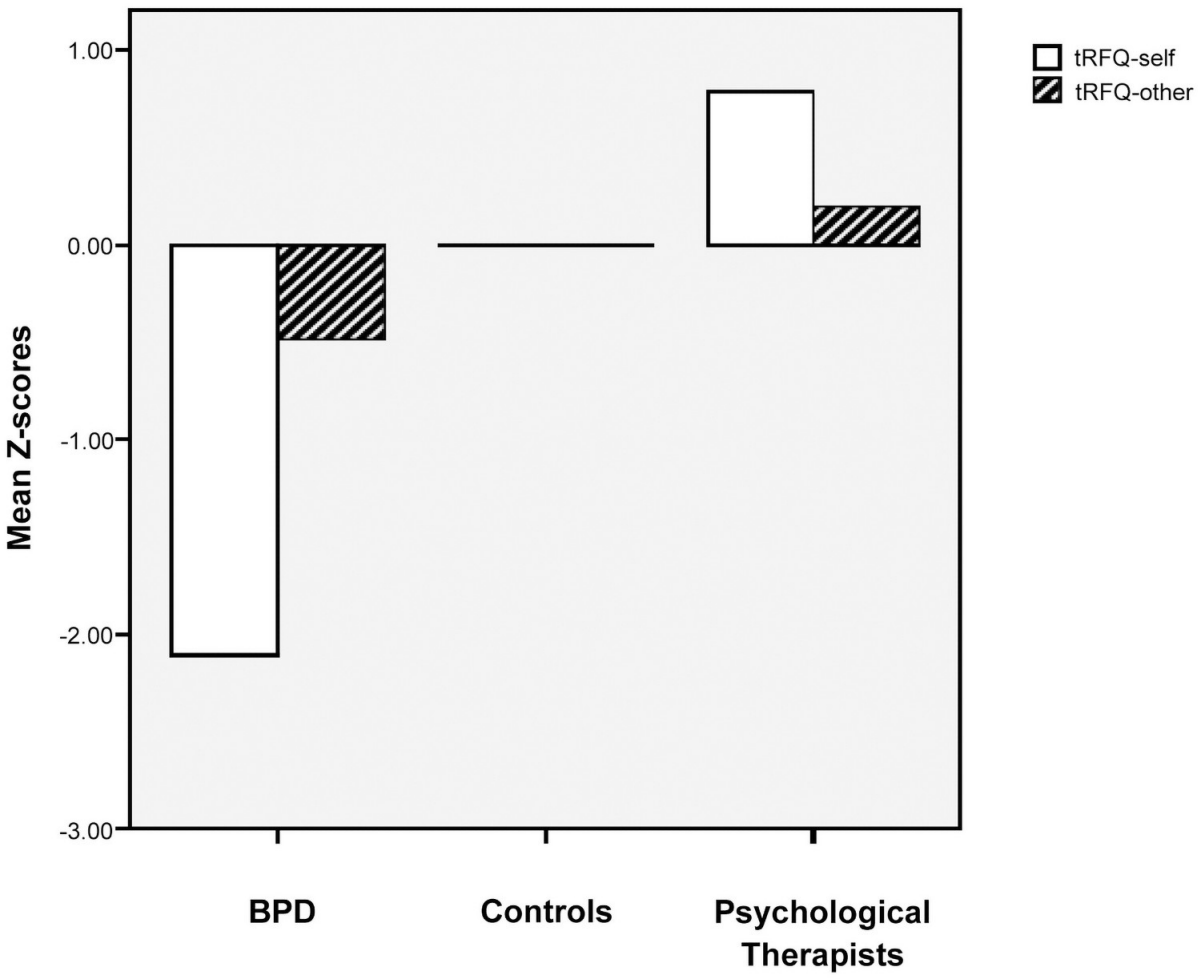
Comparing group self-other mentalizing profiles. Z-scores represent deviations from the control group mean measured in control group standard deviations.

Group mentalizing profiles for standardised RFQ-self and RFQ-other are shown in Fig 3.

T-tests were performed comparing these Z-scores for RFQ-self and RFQ-other in each group in order to measure differential impairment with reference to the control group.

#### Psychological Therapist group self-other profile

For Psychological Therapists Z-scores for RFQ-self were significantly higher than RFQ-other scores (*t*(39) = 5.01, *p* < .001, *r* = .64) with a large effect size. However, this was in the opposite direction to that hypothesized with self-mentalizing being a relative strength.

#### BPD group self-other profile

For the BPD group *Z*-scores for RFQ-self were significantly lower than those for RFQ-other in a paired *t*-test (*t*(36) = -6.76, *p* < .001, *r* = .75). Contrary to our hypothesis this indicated differential impairment in self and other mentalizing with a large effect size. Here, whilst the BPD group profile was characterised by mentalizing impairment on both subscales, the group’s relative strength was their mentalizing of others. By contrast, whilst the Psychological Therapist group scored higher on both, their relative strength was their self-mentalizing.

Bimodal distribution of RFQ-other scores led to two BPD groups being differentiated and compared on BSI12, PAI-BOR, MHV, treatment site and IMS. A significant difference was found only on the Affective Instability subscale of the PAI-BOR (*t*(35) = -2.24, *p* = .032, *r* = .35). Higher scores on this subscale indicate a ‘propensity to alternate rapidly between various negative affects and high emotional responsiveness [51].

## Discussion

With regard to self-mentalizing the RFQ18 distinguished between the BPD group, non-clinical controls and the ‘expert’ group, as hypothesized. The RFQ18 was the only measure to detect enhanced self-mentalizing in the hypothesized expert group according to self-report. With regard to mentalizing other*s*, the RFQ18 did not discriminate between the BPD group and controls, or between controls and Psychological Therapists, disconfirming our hypothesis. In controls, self-other profiles of mentalizing capacity showed differential proficiency in both hypothesized experts and BPD but in opposite directions. The BPD group reported a significant deficit in self-mentalizing only; disconfirming our hypothesis. The Psychological Therapists group reported enhanced self-mentalizing capacity only.

### BPD and mentalizing

The self-other profile of mentalizing generated here characterised more by marked self-mentalizing impairment is broadly in support of the literature to date. However, the RFQ-other subscale did not identify in BPD an impairment in cognitive empathy apparently detected by the PTS and predicted by the ‘double dissociation’ hypothesis [11]. RFQ-other is not specific to cognitive empathy and so may conflate cognitive and emotional dimensions, within this multidimensional capacity [14, 74]. However, most RFQ-other items do appear to reference thoughts and perspectives rather than feelings (S1 Text. RFQ18 self and other subscale items and RFQ54). Post-hoc analysis revealed no correlation between RFQ-other and cognitive empathy (PTS) scores. The recently developed MentS [74] measure of mentalizing, also containing subscales for self and other, has been similarly unable to discriminate BPD participants from controls [74]. However, the MentS ‘other’ subscale appears to have more items referencing feelings—a dimension of empathy thought to be less impaired in BPD [11]. In the light of the apparently acceptable internal reliability of the RFQ-other subscale, it is possible that RFQ-other items are too similar, resulting in a ‘bloated specifics’ effect [75]. However, we cannot exclude the possibility that this absence of a group effect is real rather than merely artefactual.

It was noted that the prevalence of unemployment in the BPD group was much higher than in controls (S1 Table) and this might reflect both consequences of and perpetuation of impaired mentalizing.

The distribution of RFQ-other scores in the BPD sample here appeared bimodal, suggesting two distinct groups; one perhaps characterised by more severe emotional instability, or with co-morbid personality disorders. Indeed, one of the BPD services taking part has previously recorded a mean of 2.8 personality disorder diagnoses in accepted patients [76].

### Psychological Therapists and mentalizing

The mentalizing profile of Psychological Therapists with regards to self and others showed differential proficiency here and was characterised not by enhanced mentalizing of others, but by enhanced self-mentalizing. This appears to support arguments that the activity of psychotherapy is aided by enhanced tolerance, monitoring and attentional control with regards to emotions experienced in the self [41, 47, 77]. This challenges some depictions of Psychological Therapists as preoccupied with the minds of others in the service of pathological self-state avoidance [25, 31]. If childhood adversity, followed by repair is instead an enabling factor in members of this profession [26, 30] then such processes may act particularly to enhance self-mentalizing rather than to inhibit this in favour of empathy as some have argued [31].

Conclusions by Hall et al. [42] that applied psychologists are defined as a group by their emotionally empathic capacities did not appear to be supported here. However, Harari et al. [11] highlight the capacity of Psychological Therapists to inhibit or regulate the ‘contagion’ effect of others’ emotions on the self. The possibility arises that the capacity for mentalizing of others in this profession is characterised by the opposite of what Harari et al. [11] describe in BPD. In BPD they identify enhanced emotional empathy combined with impaired cognitive empathy, Psychological Therapists’ enhanced mentalizing capacities may be defined by their ability to inhibit the impact of others’ emotions on the self.

In support of a role for inhibition of the contagion effect in Psychological Therapists, Hall et al. [42] found that low scores on the Personal Distress subscale in Psychologists orientated towards therapy were related positively to self-reported job satisfaction and effectiveness. In contrast to accounts of Psychological Therapists as globally sensitive to the minds of others, it may be that in one specific dimension of empathy they can be thought of as vitally less so. Moderating emotional empathy might facilitate, for example, the freeing of attentional resources during complex mentalizing tasks [78–80]. Alternatively, such a limiting of emotional empathy might reflect a more static impairment. It remains possible that membership of both the ‘poor’ and the ‘expert’ group here reflect some impairment of a dimension of mentalizing; rendering such labels as ‘expert or ‘poor’ simplistic. Caution about such labels is already urged with regards to BPD since a particular profile of impairment or proficiency might be the result of adaptive strategies in the face of a particular developmental niche [81].

### Limitations

There are some inherent limitations of the RFQ18; a measure of participants’ self-report of mentalizing. As such it may measure the appraisal of mentalizing capacity or, particularly in Psychological Therapists, the knowledge with which to portray oneself as proficient. Measurement in this study also took place in retrospect and outside the context of attachment relationships [82].

Those recruited by displayed questionnaire packs in London cafes might be limited in their demographic diversity. Comparisons of the control group scores with those of other studies suggest that if our controls differ from the wider non-clinical population, it is in the direction of being less alexithymic. This is a construct related to mentalizing self [3] and in the present study TAS scores were strongly related to RFQ-self (*r* = -.69, *p* < .01) across groups. If our control group was in this respect more mentalizing than the wider population, the superiority of psychological therapist scores on RFQ-self may be underestimated here, whilst the inferiority of BPD RFQ-self scores might be somewhat over-estimated. This latter group difference however, is widely demonstrated elsewhere [74].

The Psychological Therapist sample here were mainly trainee Clinical and Counselling Psychologists and so were highly selective in this respect. They were also 100% employed in contrast to the BPD group’s low employment rate. It was surprising that no significant difference in verbal intelligence was found between groups. A Psychological Therapist group that was largely doctorate level educated would be expected to score higher than both other groups.

#### Future research and implications

This study lends support to the multidimensional nature of the construct of mentalization as our results suggest that differential impairment or proficiency on self and other dimensions of mentalizing is possible. Distinguishing between self and other mentalizing is highly relevant to treatment of borderline personality disorder and the results here add support for a focus on self-mentalizing in treatment. The study of dimensions of mentalizing in both impaired and expert groups may help to identify etiology of impairments and mechanisms of change in these capacities. Further study is needed to replicate these findings groups who would be expected to have mentalizing expertise such as other “caring professions” (paramedics, surgeons, primary school teachers e.g. [83]).

If selection for the role of Psychological Therapists by employers and courses is to be linked with superior self-mentalizing this proficiency would have to be shown to be associated with positive outcomes. The empirical evidence base for this appears to be building [30].

Further work in testing the validity of the RFQ-other scale might address the development of separate cognitive and emotional dimensions for both self and other. Measures of emotional empathy and personal distress could be used alongside mentalization measures to explore further their role in the mentalizing profile of Psychological Therapists.

## Summary and conclusions

These results suggest that in Psychological Therapists, an active interest in the minds of others reflects at least in part a superior capacity to self-reflect. In addition to the ability to observe and tolerate self-states, it may be that a vital capacity in Psychological Therapists is the ability to inhibit or regulate the contagion effect whilst engaged in emotionally empathic tasks.

The multidimensional profile approach here to comparing mentalizing in two groups described as ’poor’ and ’expert’ demonstrates how these descriptive adjectives may suffer from the conflating of somewhat independent dimensions of mentalizing self and others; emotionally and cognitively. However, these results show for the first time that, elaborating upon this construct, it is possible to identify groups of people selected for or nurtured for mentalizing expertise.

## Supporting information

S1 Dataset 15.07.11. (SAV)

S1 Fig Consort diagram. (PDF)

S2 Fig RFQ-other histograms by group. (PDF)

S1 Presentation RFQ54 validation. (PPT)

S1 Text RFQ54 with self and other subscales. (PDF)

S2 Text Participants information sheet. (PDF)

S3 Text Questionnaire pack. (PDF)

S1 Table Demographics. (PDF)

S2 Table Relationships between potential confounds and outcome variables. (PDF)

S3 Table Descriptive statistics for outcome measures. (PDF)

S4 Table Correlation matrix for continuous variables. (PDF)

S5 Table Normality of untransformed variables. (PDF)

S6 Table Normality of transformed variables. (PDF)

S7 Table Normality of residuals of main outcome measures. (PDF)
