# Supplementary material for: ‘Rich’ and ‘poor’ in mentalizing: Do expert mentalizers exist?
Source: PLoS One. 2021 Oct 25;16(10):e0259030. doi: 10.1371/journal.pone.0259030 (PMC8544847; doi:10.1371/journal.pone.0259030)
Supplement: S1 Fig — (PDF) [file pone.0259030.s002.pdf]

S1 Fig. Consort diagram

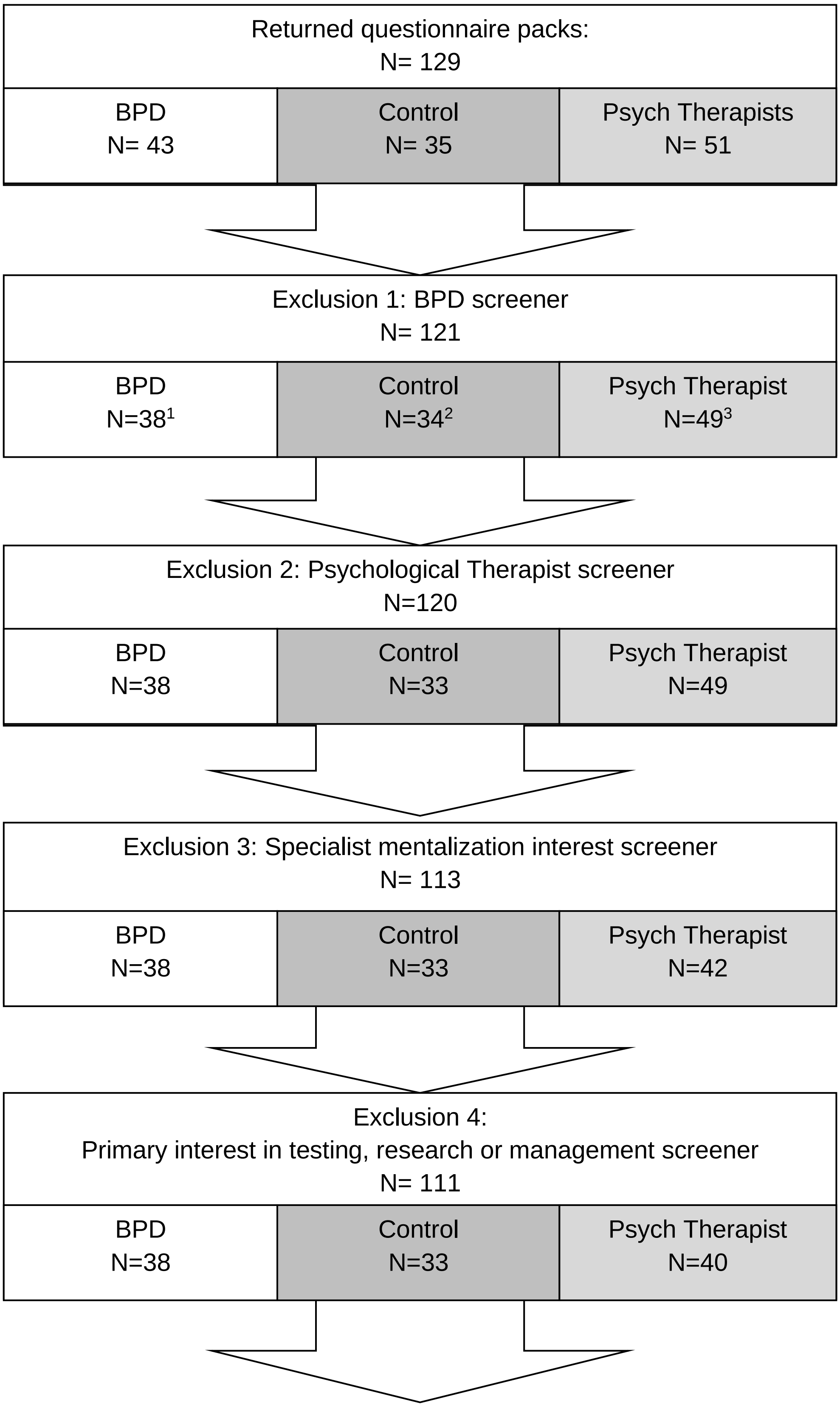

[1] Three exclusions due to missing values on PAI-BOR, two due to scores below cut-off.
[2] One exclusion due to missing values on PAI-BOR.
[3] Two exclusions due to scores above cut-off.
