# Supplementary figures and images for: ‘Rich’ and ‘poor’ in mentalizing: Do expert mentalizers exist?
Source: PLoS One. 2021 Oct 25;16(10):e0259030. doi: 10.1371/journal.pone.0259030 (PMC8544847; doi:10.1371/journal.pone.0259030)

**S2 Fig. RFQ-Other Histograms by group**

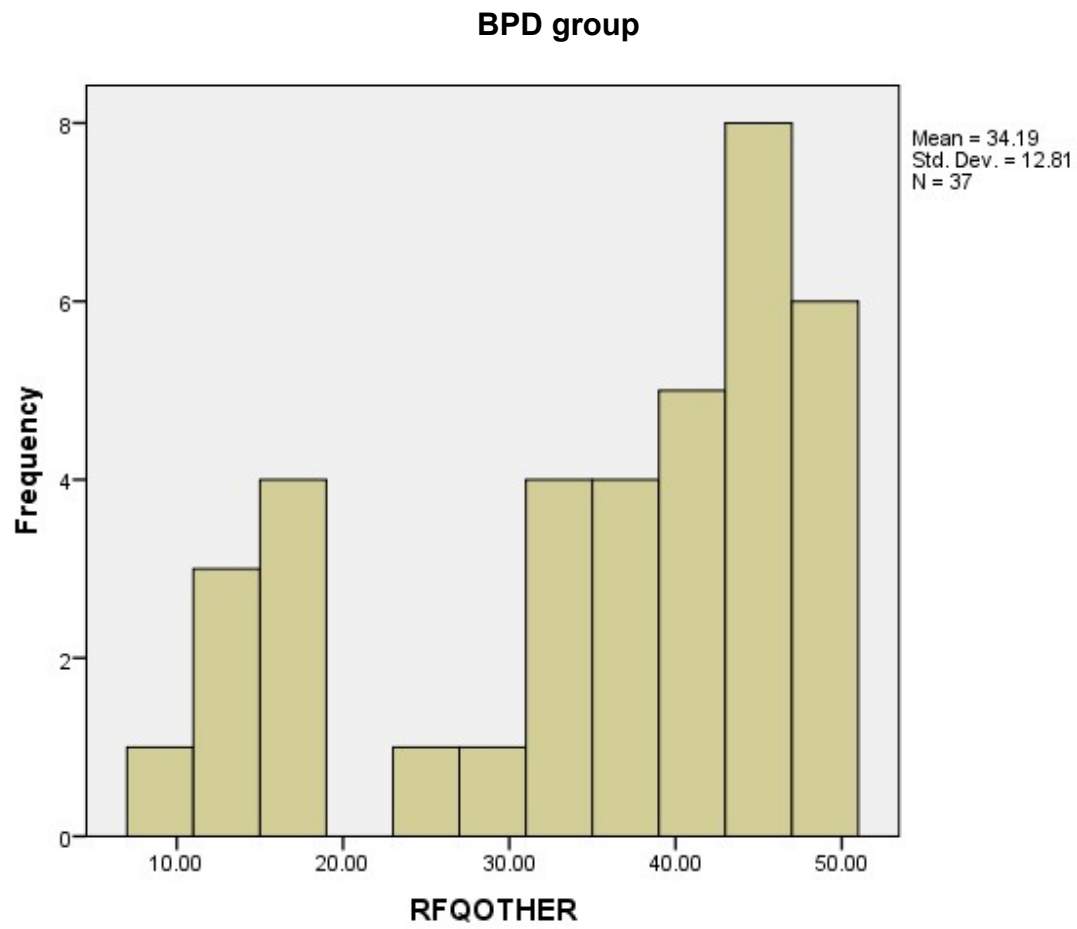

### Control Group

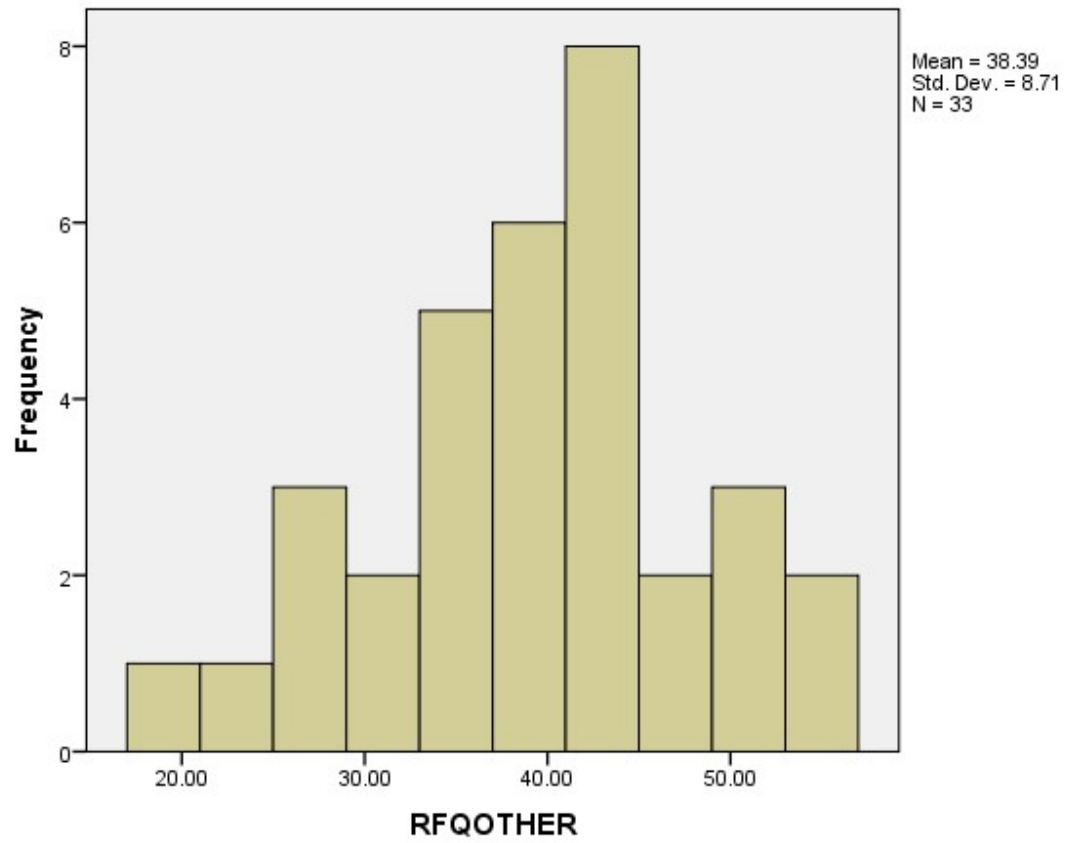

### Psychological Therapist Group

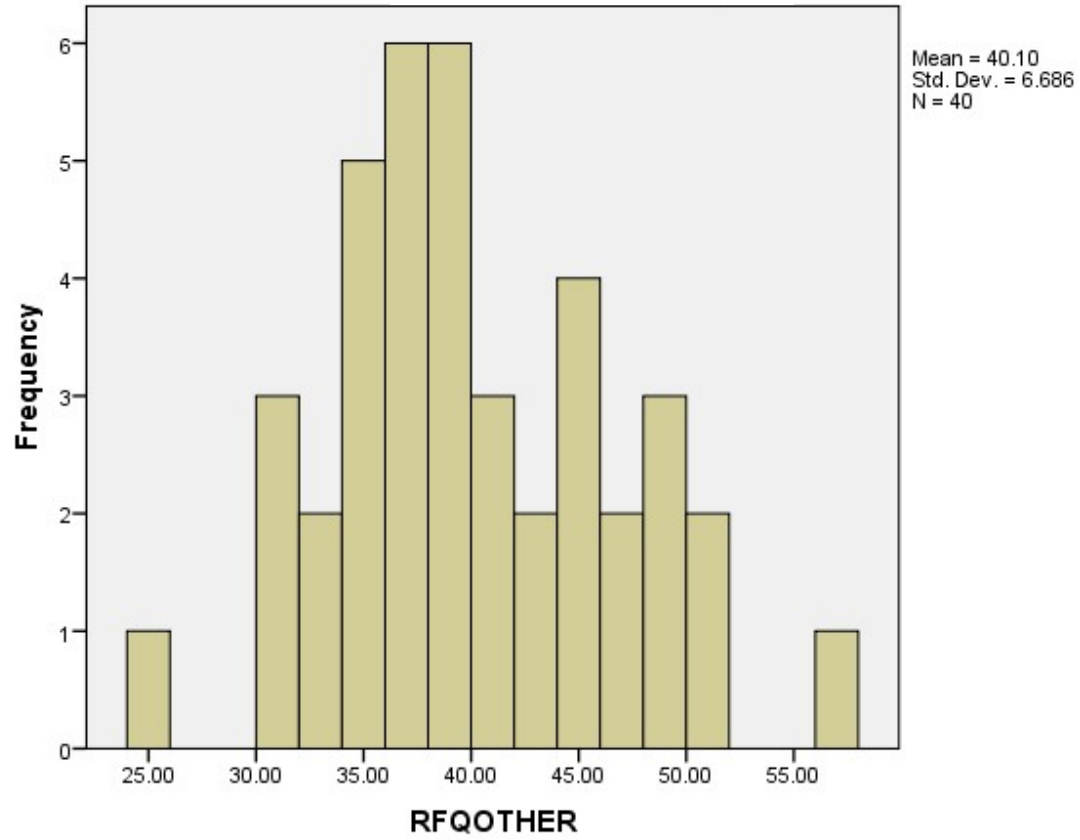

Supplement: S2 Fig — (PDF) [file pone.0259030.s003.pdf]
