# Supplementary material for: ‘Rich’ and ‘poor’ in mentalizing: Do expert mentalizers exist?
Source: PLoS One. 2021 Oct 25;16(10):e0259030. doi: 10.1371/journal.pone.0259030 (PMC8544847; doi:10.1371/journal.pone.0259030)
Supplement: S1 Presentation — (PPT) [file pone.0259030.s004.ppt]

## Slide 1
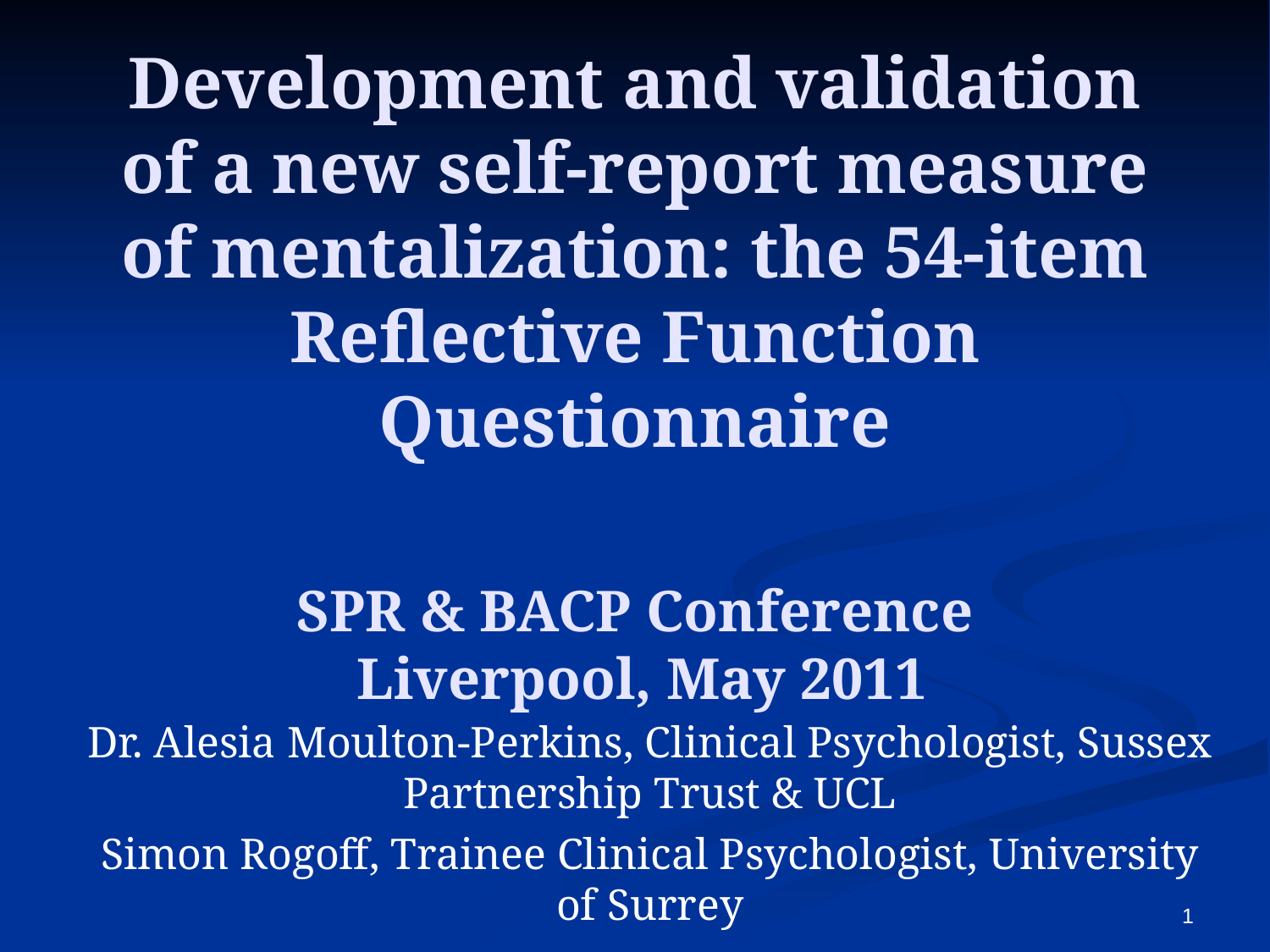

# Development and validation of a new self-report measure of mentalization: the 54-item Reflective Function Questionnaire SPR & BACP Conference Liverpool, May 2011
Dr. Alesia Moulton-Perkins, Clinical Psychologist, Sussex Partnership Trust & UCL
Simon Rogoff, Trainee Clinical Psychologist, University of Surrey
<number>

## Slide 2
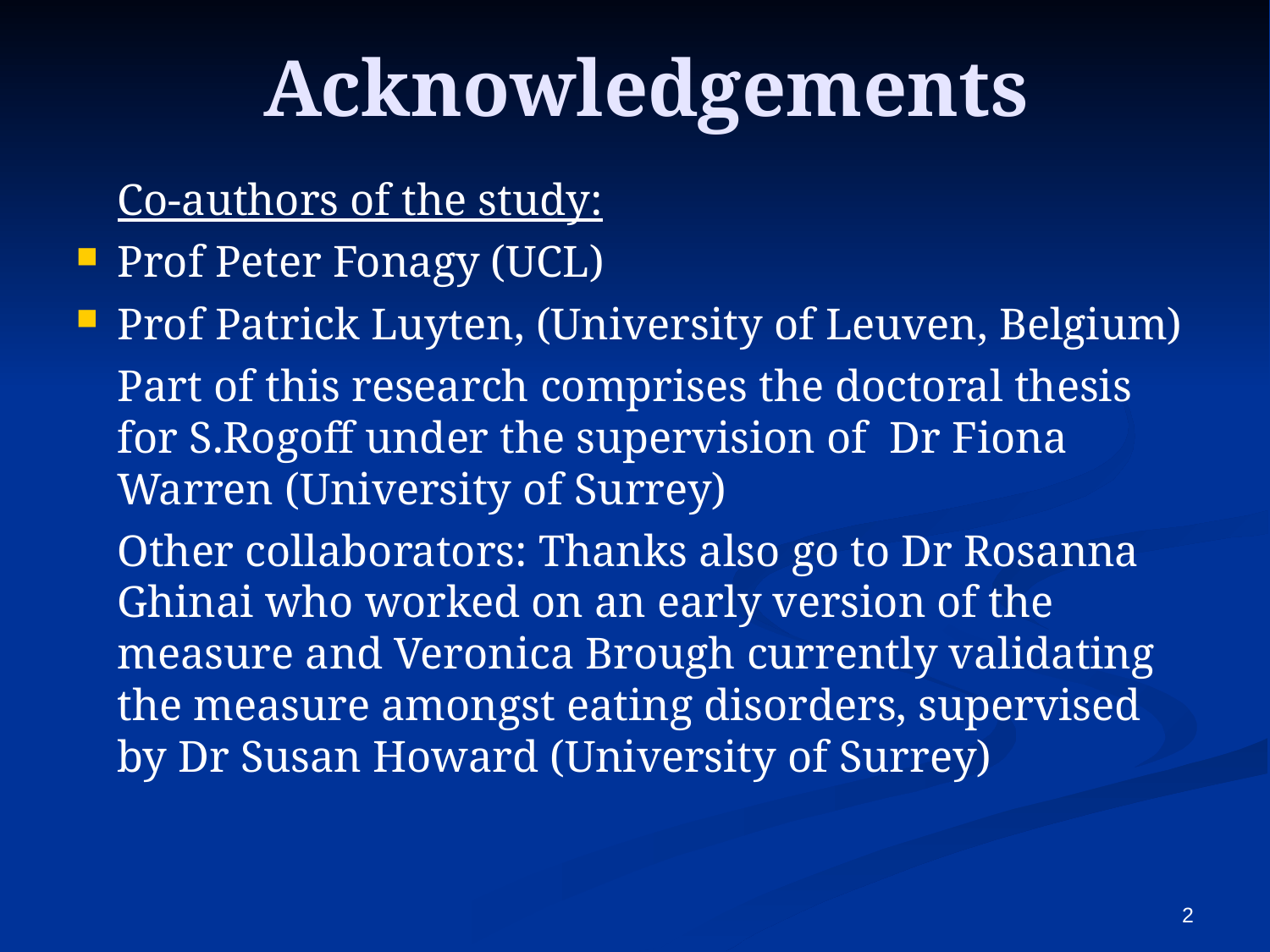

# Acknowledgements
Co-authors of the study:
Prof Peter Fonagy (UCL)
Prof Patrick Luyten, (University of Leuven, Belgium)
Part of this research comprises the doctoral thesis for S.Rogoff under the supervision of Dr Fiona Warren (University of Surrey)
Other collaborators: Thanks also go to Dr Rosanna Ghinai who worked on an early version of the measure and Veronica Brough currently validating the measure amongst eating disorders, supervised by Dr Susan Howard (University of Surrey)
<number>

## Slide 3
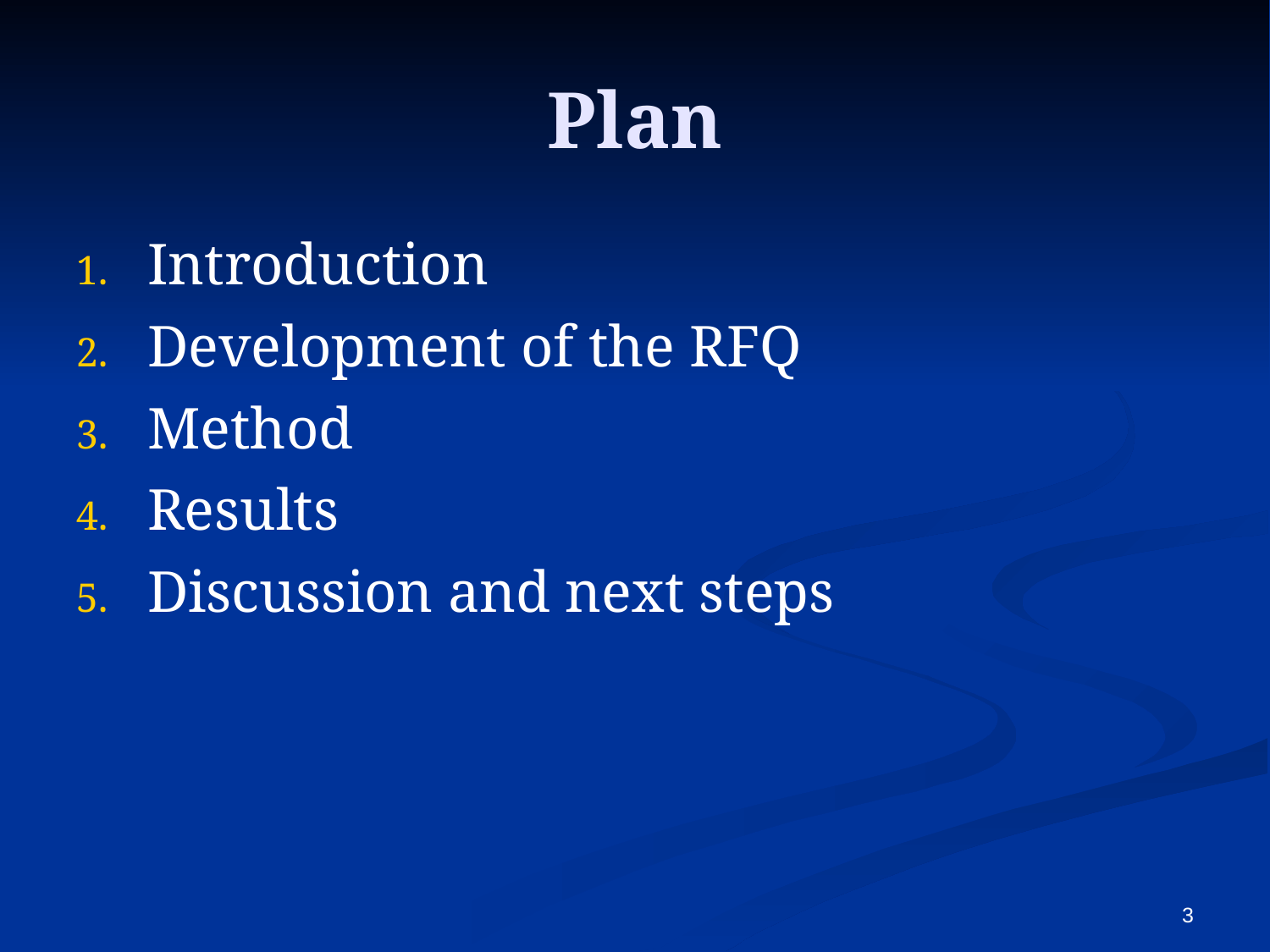

# Plan
Introduction
Development of the RFQ
Method
Results
Discussion and next steps
<number>

## Slide 4
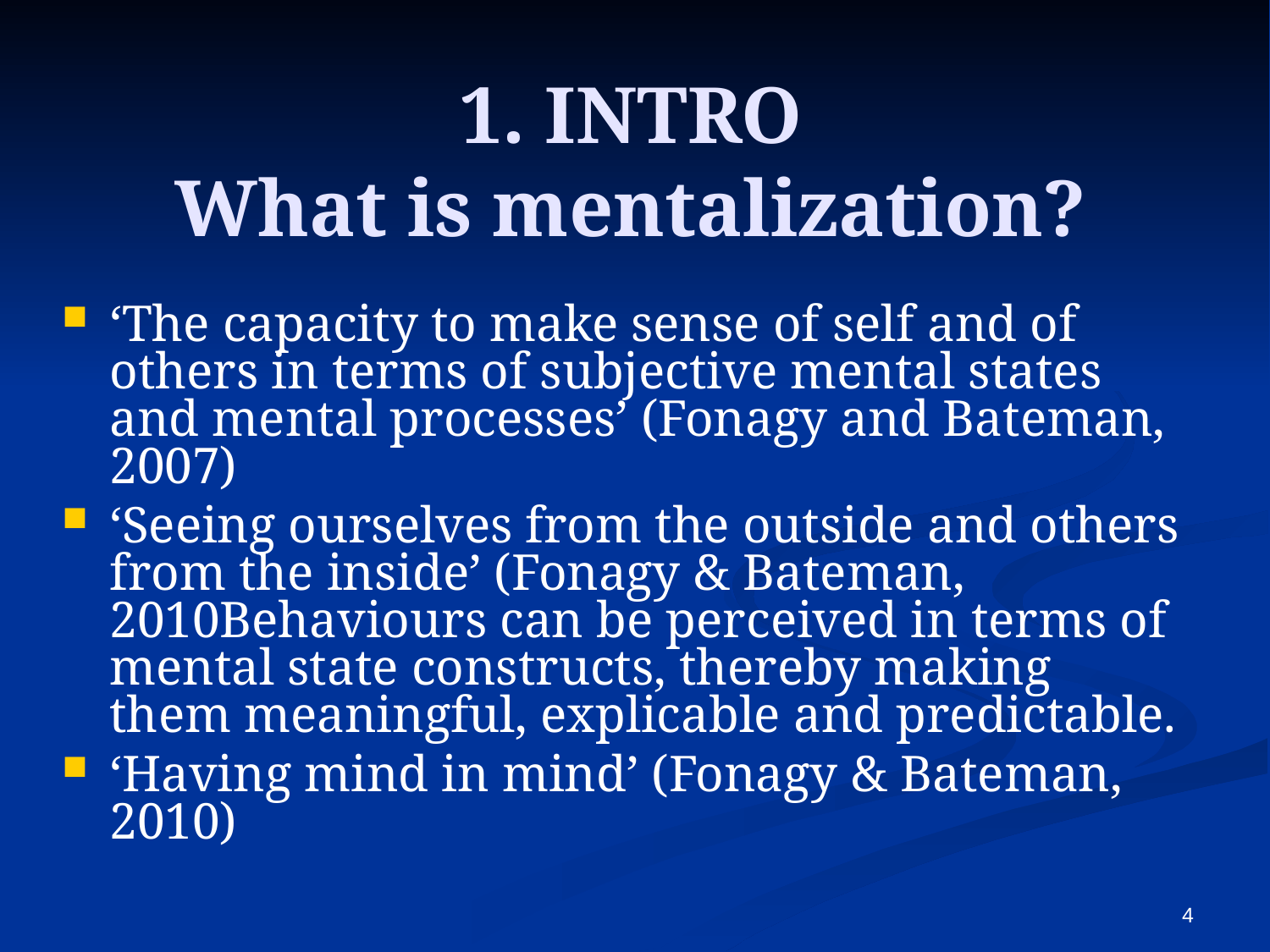

# 1. INTRO What is mentalization?
‘The capacity to make sense of self and of others in terms of subjective mental states and mental processes’ (Fonagy and Bateman, 2007)
‘Seeing ourselves from the outside and others from the inside’ (Fonagy & Bateman, 2010Behaviours can be perceived in terms of mental state constructs, thereby making them meaningful, explicable and predictable.
‘Having mind in mind’ (Fonagy & Bateman, 2010)
<number>

## Slide 5
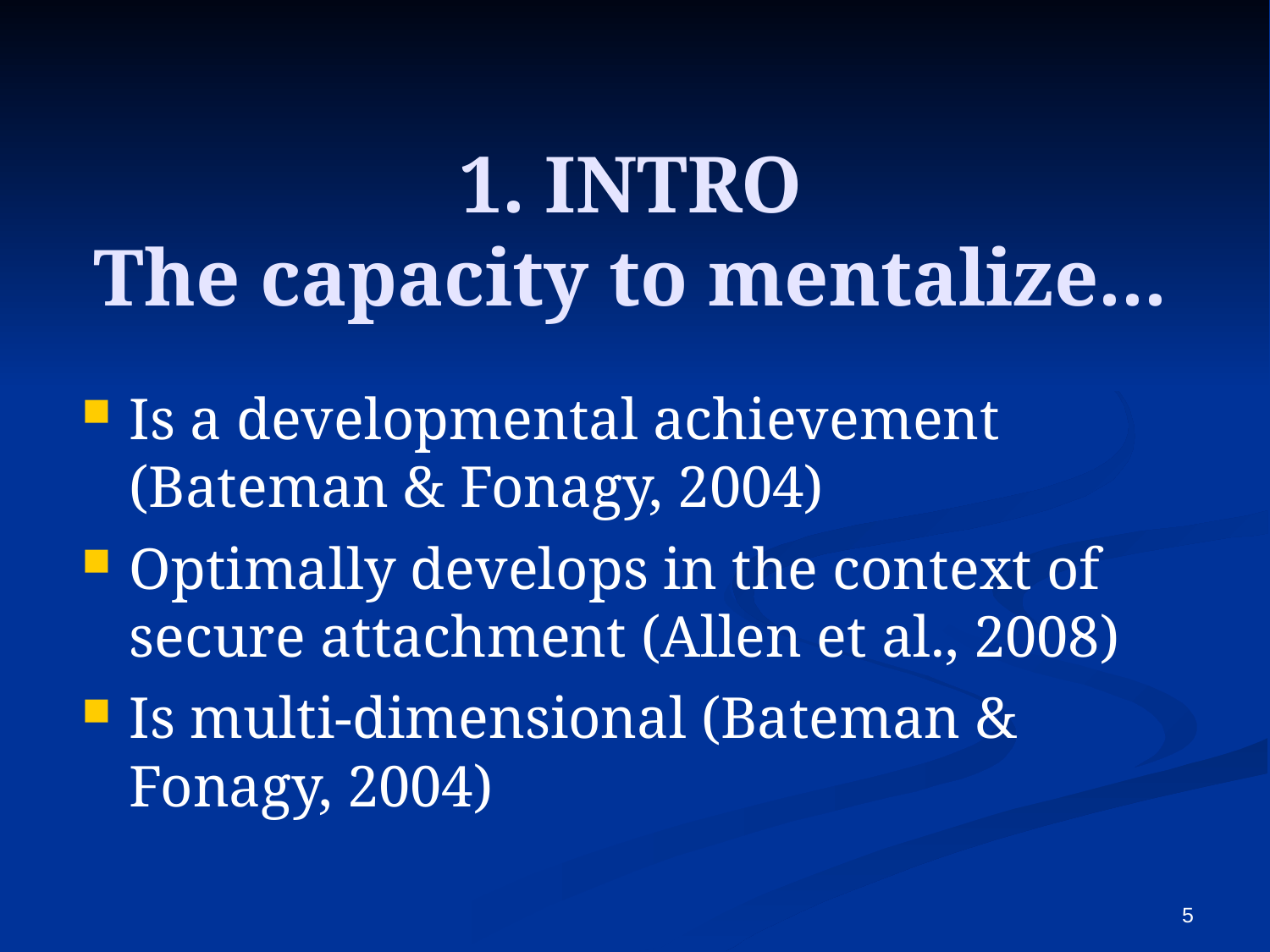

# 1. INTRO The capacity to mentalize...
Is a developmental achievement (Bateman & Fonagy, 2004)
Optimally develops in the context of secure attachment (Allen et al., 2008)
Is multi-dimensional (Bateman & Fonagy, 2004)
<number>

## Slide 6
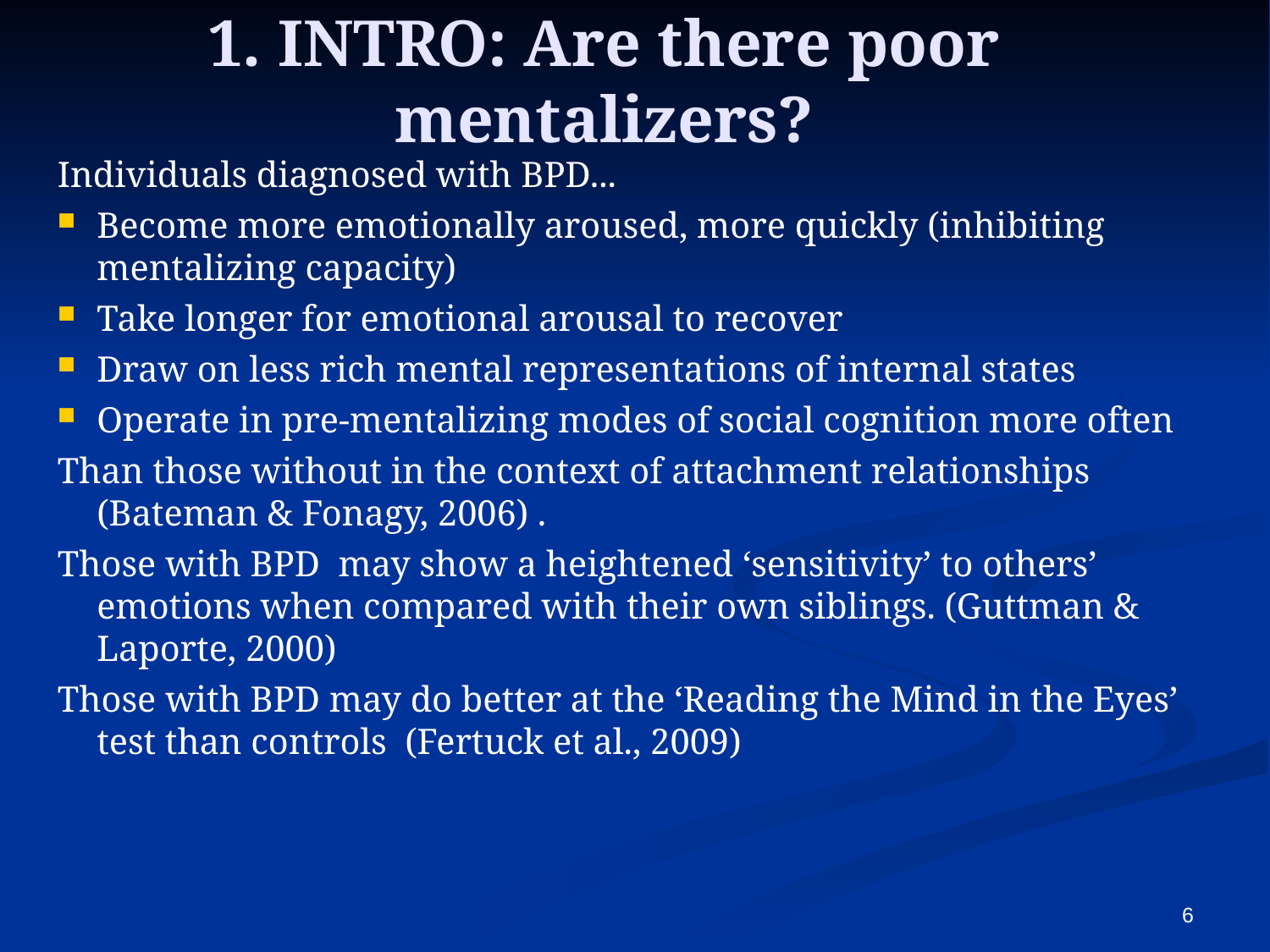

# 1. INTRO: Are there poor mentalizers?
Individuals diagnosed with BPD...
Become more emotionally aroused, more quickly (inhibiting mentalizing capacity)
Take longer for emotional arousal to recover
Draw on less rich mental representations of internal states
Operate in pre-mentalizing modes of social cognition more often
Than those without in the context of attachment relationships (Bateman & Fonagy, 2006) .
Those with BPD may show a heightened ‘sensitivity’ to others’ emotions when compared with their own siblings. (Guttman & Laporte, 2000)
Those with BPD may do better at the ‘Reading the Mind in the Eyes’ test than controls (Fertuck et al., 2009)
<number>

## Slide 7
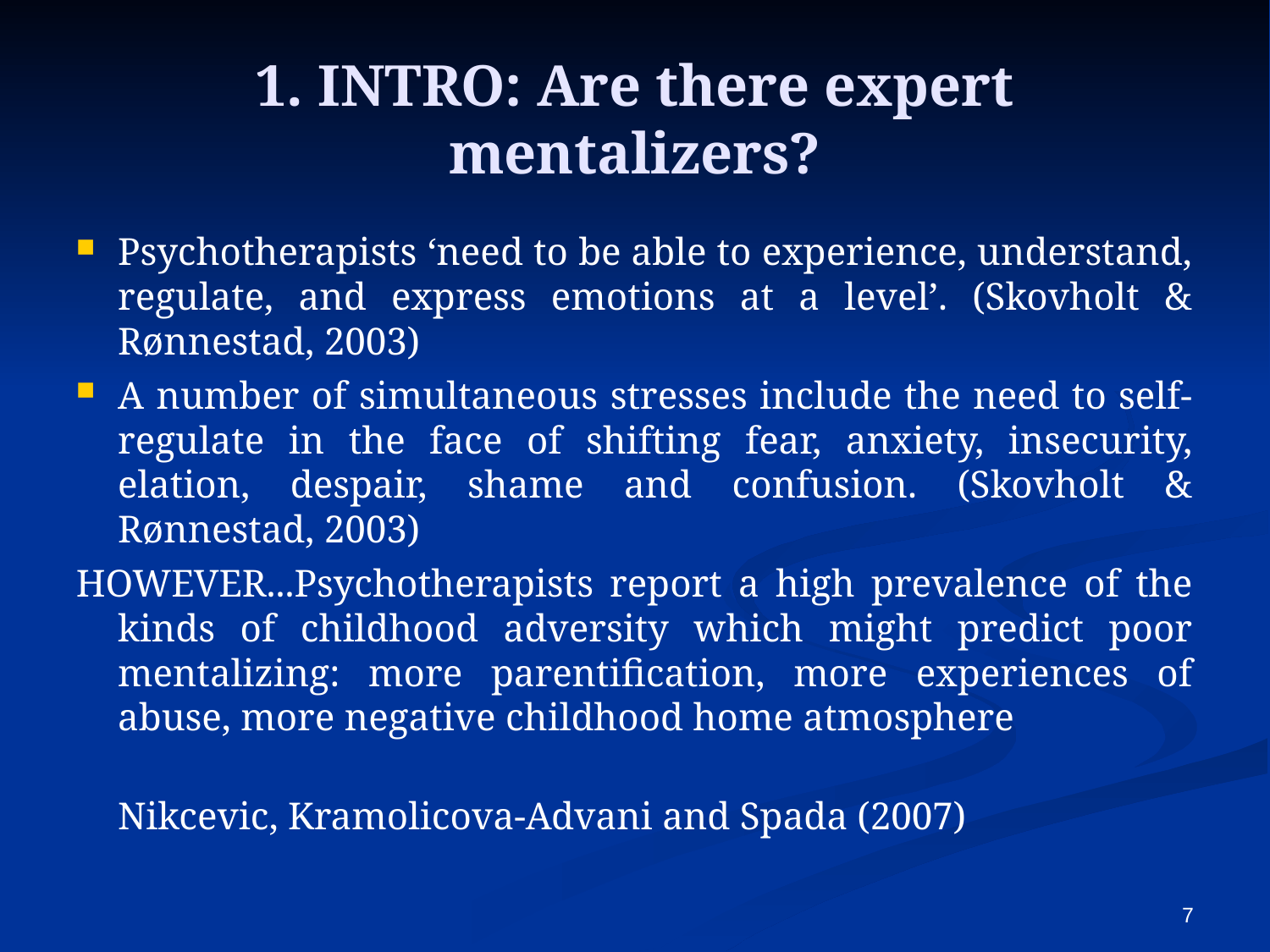

# 1. INTRO: Are there expert mentalizers?
Psychotherapists ‘need to be able to experience, understand, regulate, and express emotions at a level’. (Skovholt & Rønnestad, 2003)
A number of simultaneous stresses include the need to self-regulate in the face of shifting fear, anxiety, insecurity, elation, despair, shame and confusion. (Skovholt & Rønnestad, 2003)
HOWEVER...Psychotherapists report a high prevalence of the kinds of childhood adversity which might predict poor mentalizing: more parentification, more experiences of abuse, more negative childhood home atmosphere
	Nikcevic, Kramolicova-Advani and Spada (2007)
<number>

## Slide 8
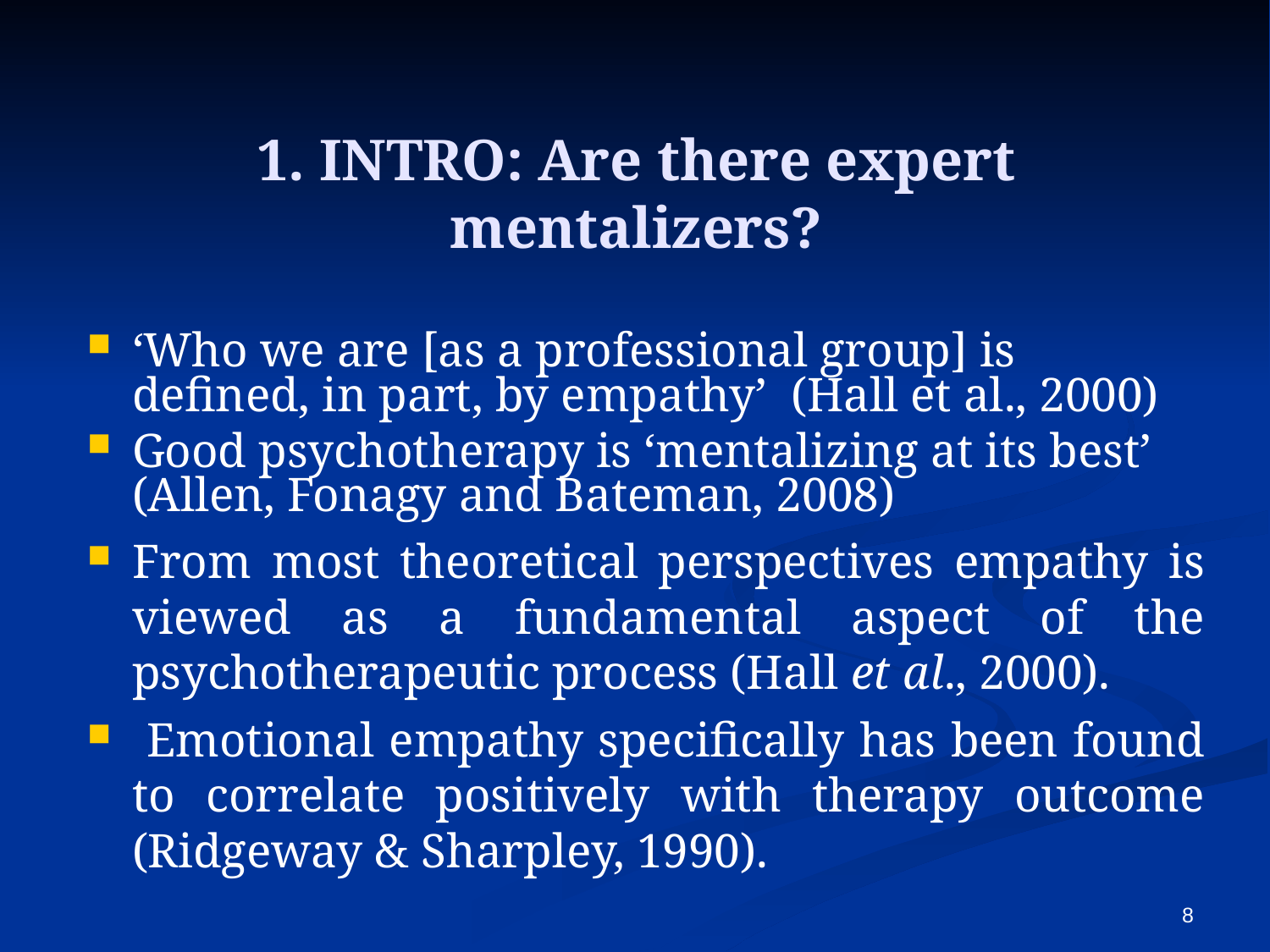

# 1. INTRO: Are there expert mentalizers?
‘Who we are [as a professional group] is defined, in part, by empathy’ (Hall et al., 2000)
Good psychotherapy is ‘mentalizing at its best’ (Allen, Fonagy and Bateman, 2008)
From most theoretical perspectives empathy is viewed as a fundamental aspect of the psychotherapeutic process (Hall et al., 2000).
 Emotional empathy specifically has been found to correlate positively with therapy outcome (Ridgeway & Sharpley, 1990).
<number>

## Slide 9
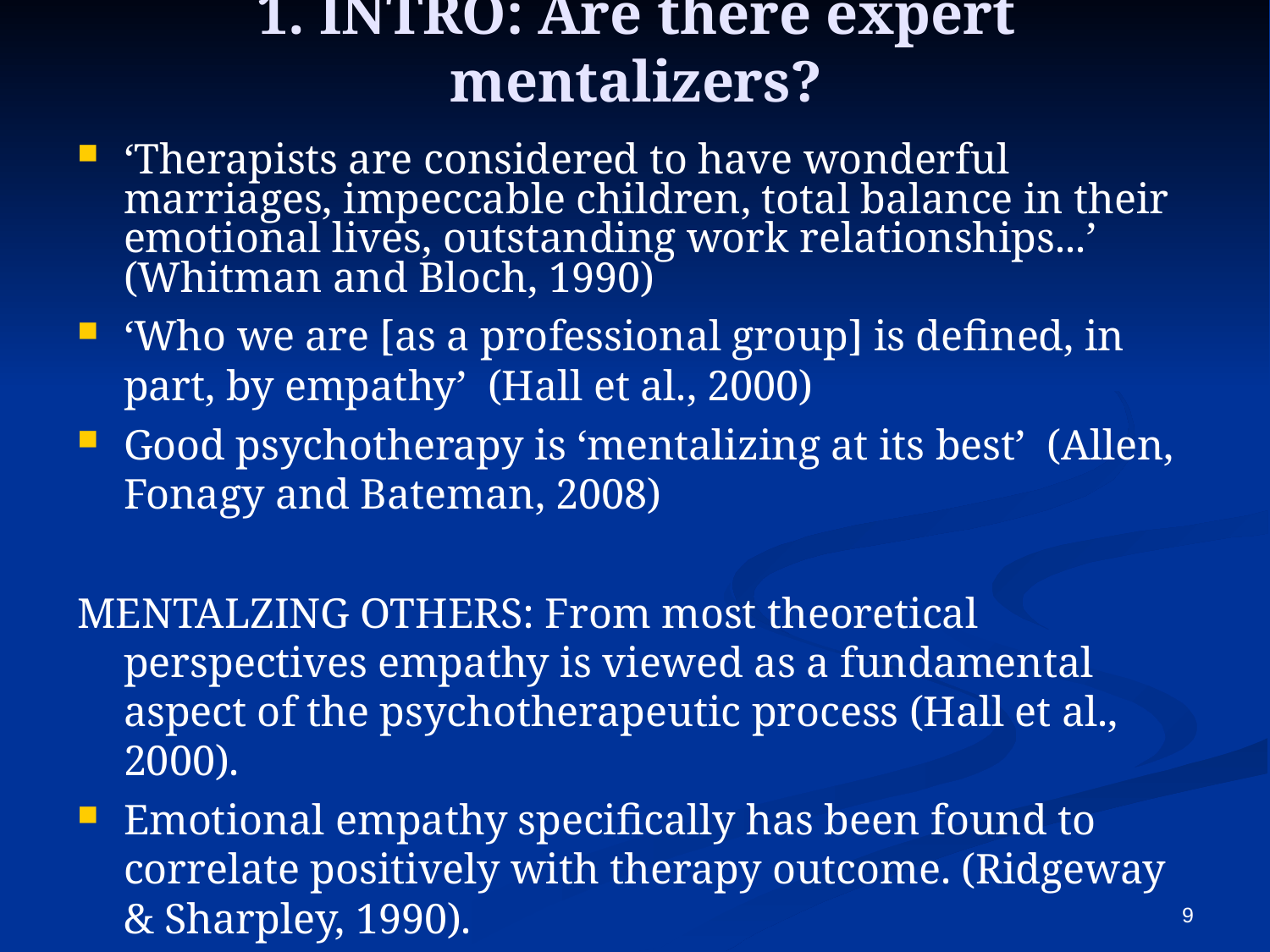

1. INTRO: Are there expert mentalizers?
‘Therapists are considered to have wonderful marriages, impeccable children, total balance in their emotional lives, outstanding work relationships...’ (Whitman and Bloch, 1990)
‘Who we are [as a professional group] is defined, in part, by empathy’ (Hall et al., 2000)
Good psychotherapy is ‘mentalizing at its best’ (Allen, Fonagy and Bateman, 2008)
MENTALZING OTHERS: From most theoretical perspectives empathy is viewed as a fundamental aspect of the psychotherapeutic process (Hall et al., 2000).
Emotional empathy specifically has been found to correlate positively with therapy outcome. (Ridgeway & Sharpley, 1990).
<number>

## Slide 10
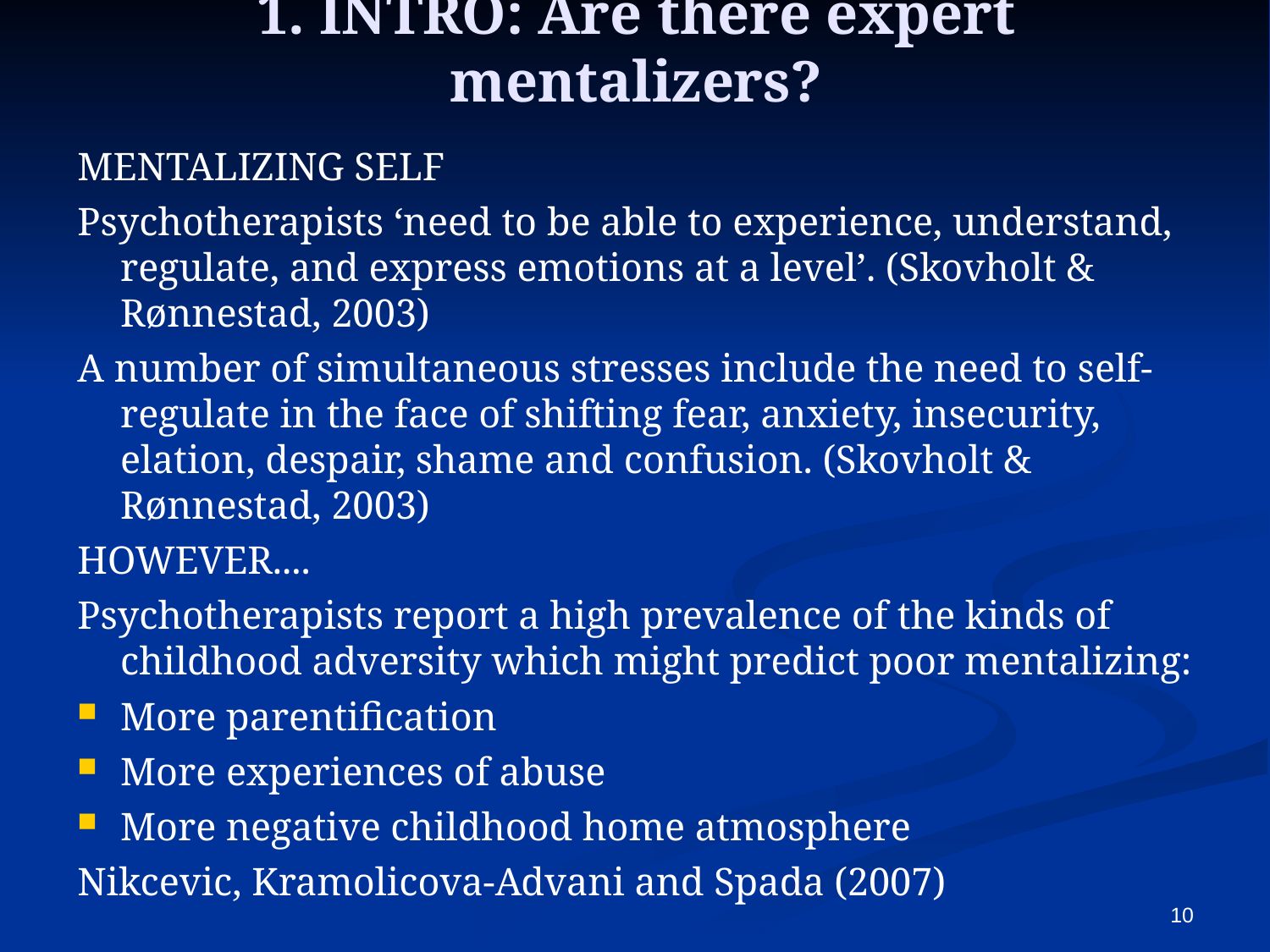

1. INTRO: Are there expert mentalizers?
MENTALIZING SELF
Psychotherapists ‘need to be able to experience, understand, regulate, and express emotions at a level’. (Skovholt & Rønnestad, 2003)
A number of simultaneous stresses include the need to self-regulate in the face of shifting fear, anxiety, insecurity, elation, despair, shame and confusion. (Skovholt & Rønnestad, 2003)
HOWEVER....
Psychotherapists report a high prevalence of the kinds of childhood adversity which might predict poor mentalizing:
More parentification
More experiences of abuse
More negative childhood home atmosphere
Nikcevic, Kramolicova-Advani and Spada (2007)
<number>

## Slide 11
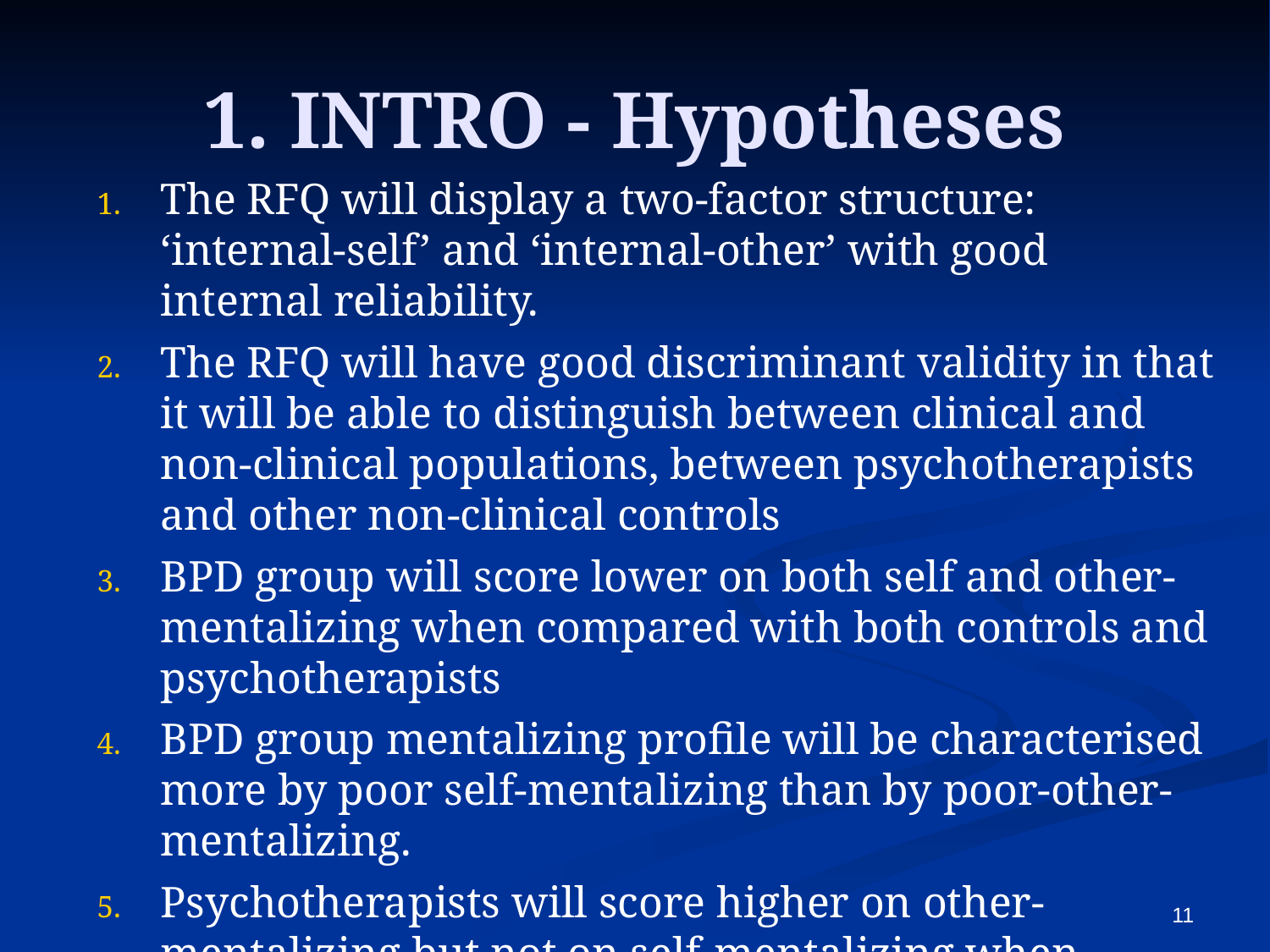

# 1. INTRO - Hypotheses
The RFQ will display a two-factor structure: ‘internal-self’ and ‘internal-other’ with good internal reliability.
The RFQ will have good discriminant validity in that it will be able to distinguish between clinical and non-clinical populations, between psychotherapists and other non-clinical controls
BPD group will score lower on both self and other-mentalizing when compared with both controls and psychotherapists
BPD group mentalizing profile will be characterised more by poor self-mentalizing than by poor-other-mentalizing.
Psychotherapists will score higher on other-mentalizing but not on self-mentalizing when compared with controls.
(other hypotheses will be tested in final study)
<number>

## Slide 12
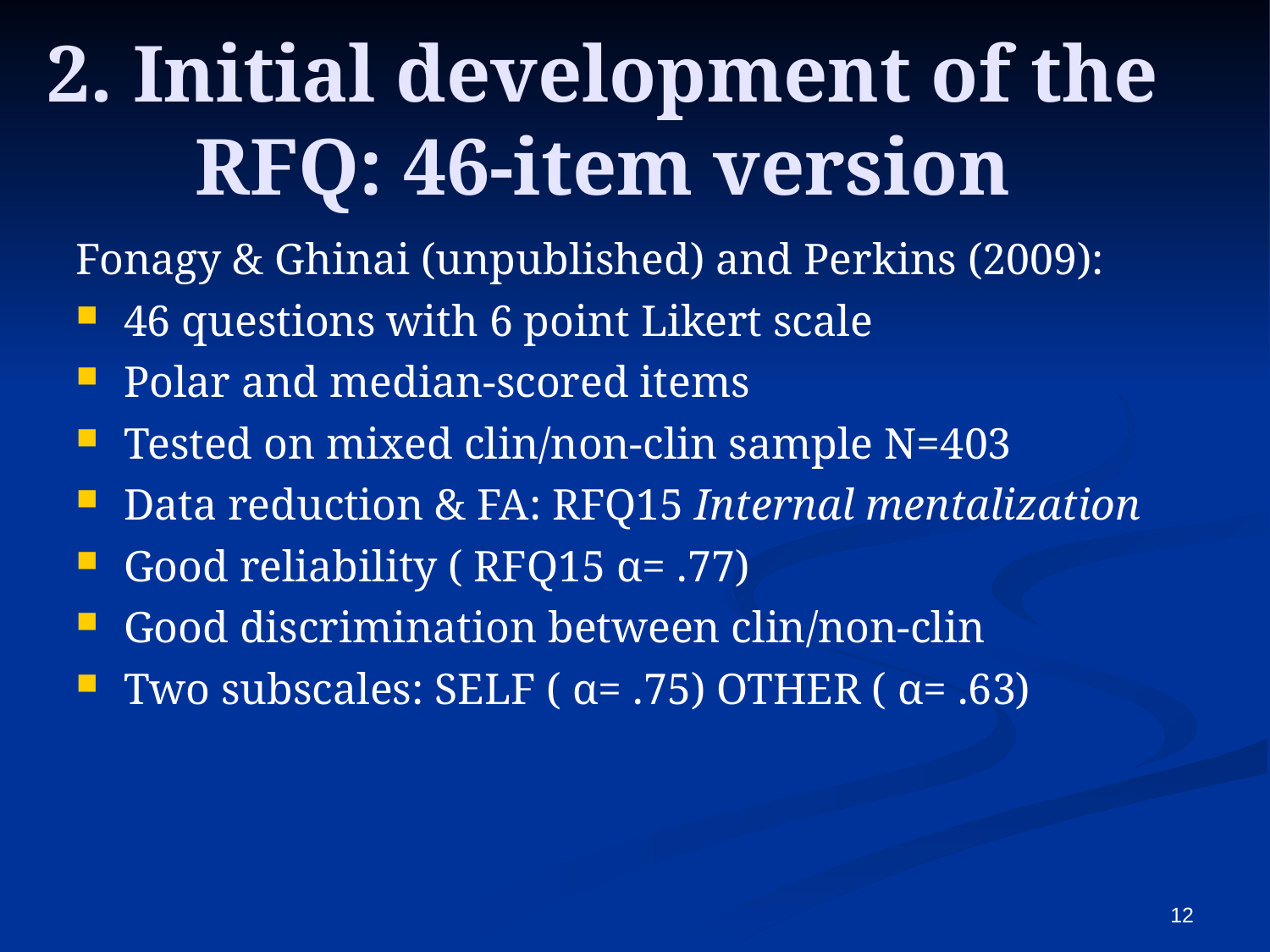

# 2. Initial development of the RFQ: 46-item version
Fonagy & Ghinai (unpublished) and Perkins (2009):
46 questions with 6 point Likert scale
Polar and median-scored items
Tested on mixed clin/non-clin sample N=403
Data reduction & FA: RFQ15 Internal mentalization
Good reliability ( RFQ15 α= .77)
Good discrimination between clin/non-clin
Two subscales: SELF ( α= .75) OTHER ( α= .63)
<number>

## Slide 13
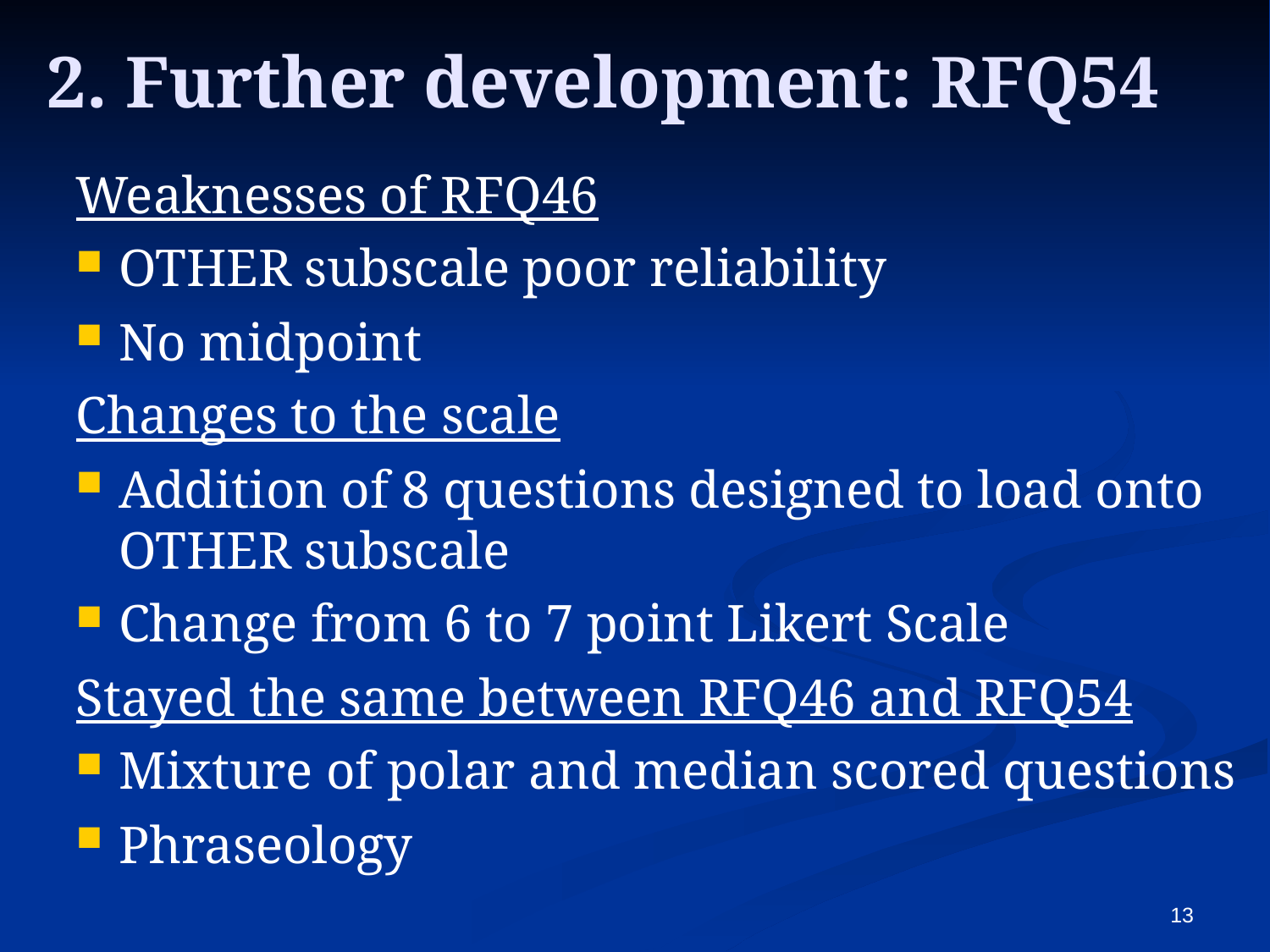

# 2. Further development: RFQ54
Weaknesses of RFQ46
OTHER subscale poor reliability
No midpoint
Changes to the scale
Addition of 8 questions designed to load onto OTHER subscale
Change from 6 to 7 point Likert Scale
Stayed the same between RFQ46 and RFQ54
Mixture of polar and median scored questions
Phraseology
<number>

## Slide 14
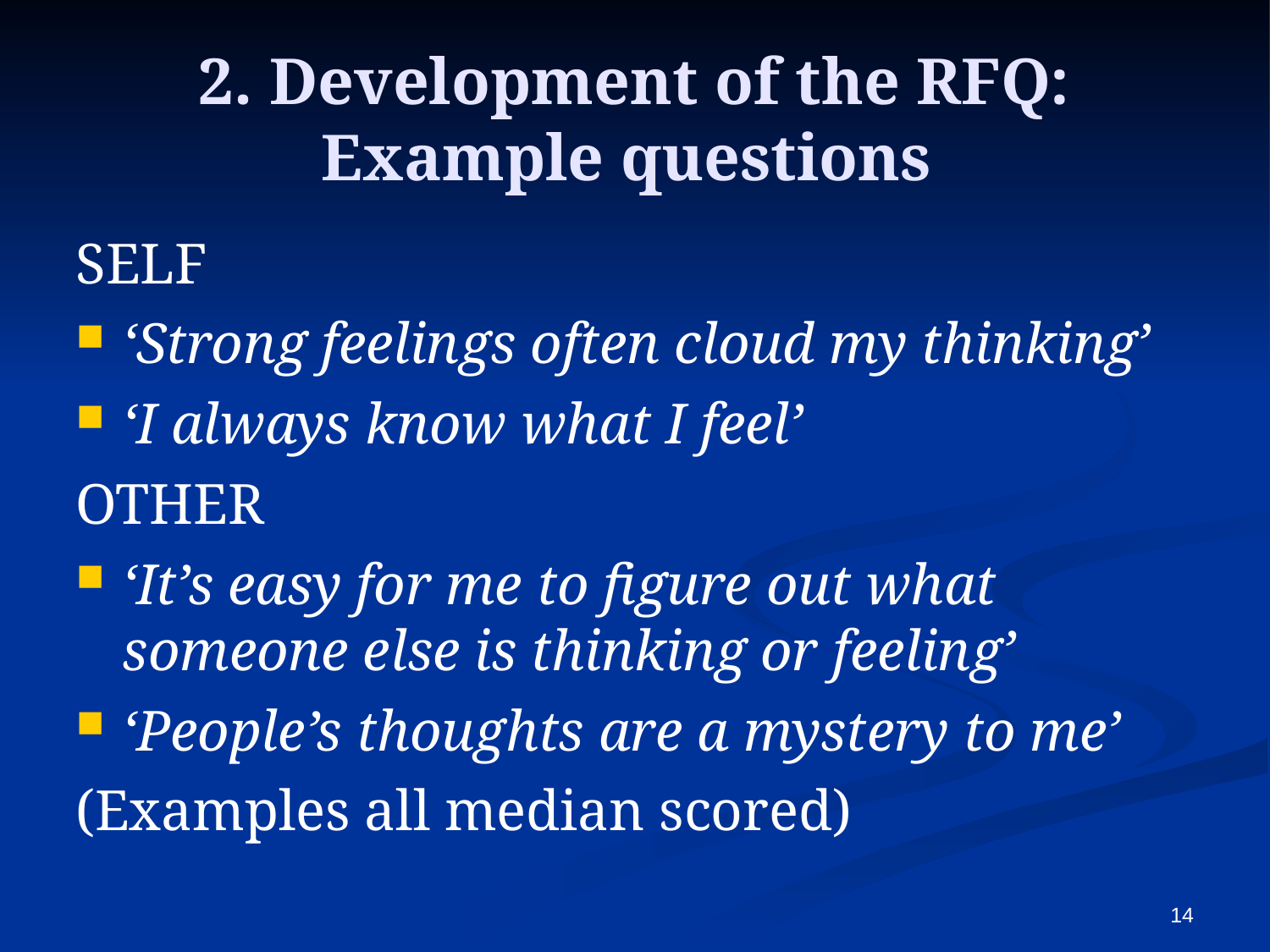

# 2. Development of the RFQ: Example questions
SELF
‘Strong feelings often cloud my thinking’
‘I always know what I feel’
OTHER
‘It’s easy for me to figure out what someone else is thinking or feeling’
‘People’s thoughts are a mystery to me’
(Examples all median scored)
<number>

## Slide 15
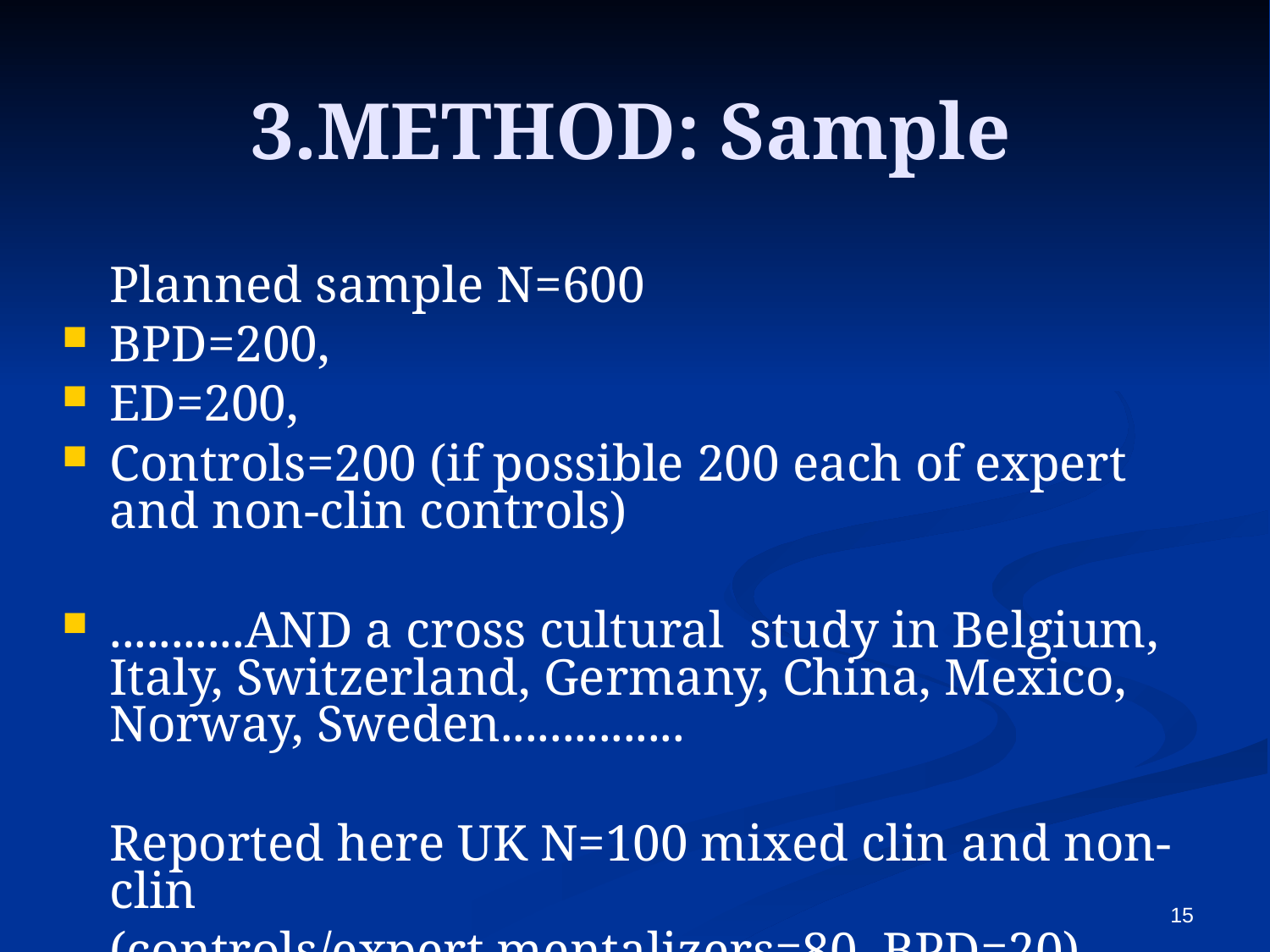

# 3.METHOD: Sample
Planned sample N=600
BPD=200,
ED=200,
Controls=200 (if possible 200 each of expert and non-clin controls)
...........AND a cross cultural study in Belgium, Italy, Switzerland, Germany, China, Mexico, Norway, Sweden...............
Reported here UK N=100 mixed clin and non-clin
(controls/expert mentalizers=80, BPD=20)
<number>

## Slide 16
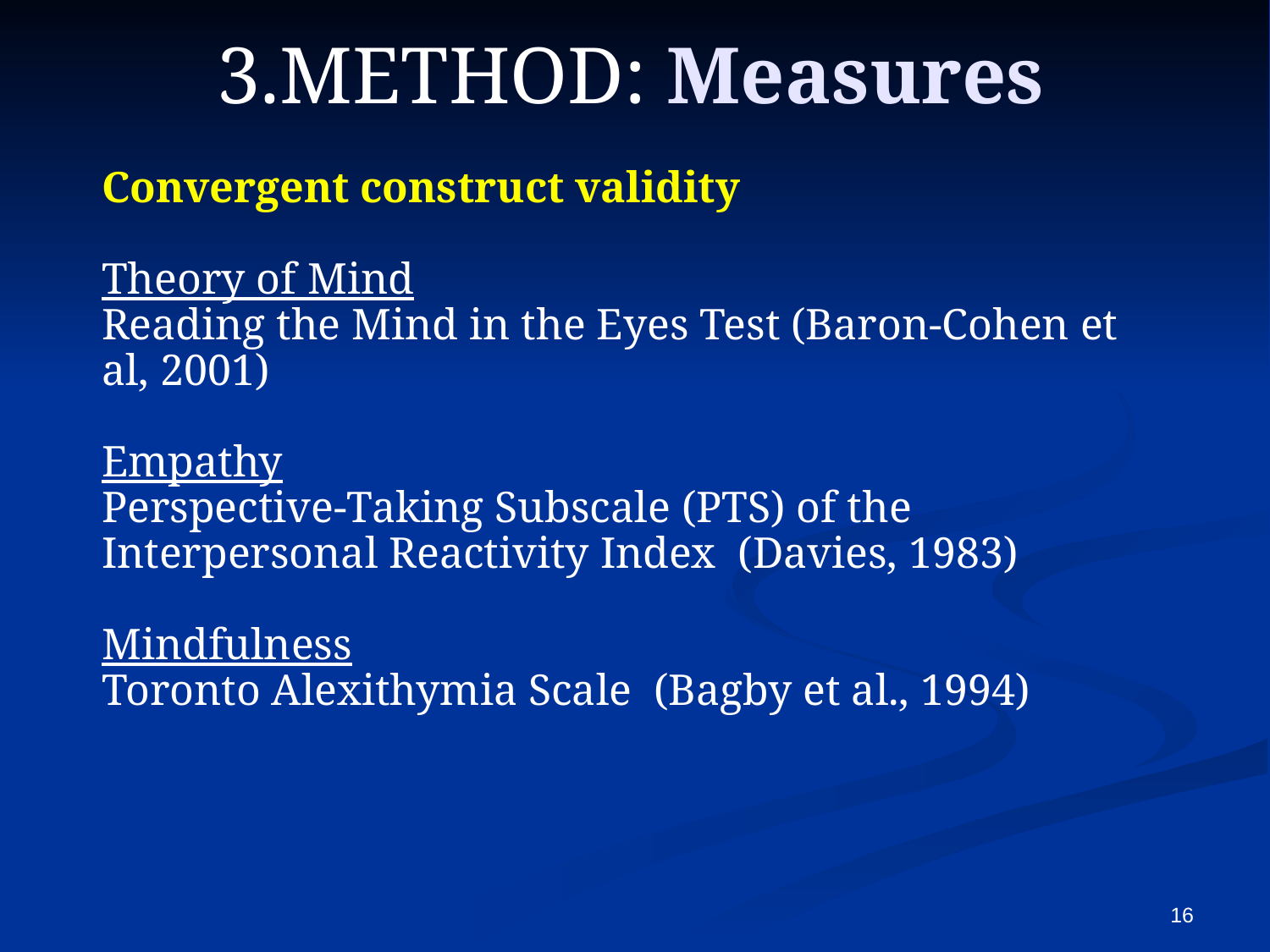

3.METHOD: Measures
Convergent construct validity
Theory of Mind
Reading the Mind in the Eyes Test (Baron-Cohen et al, 2001)
Empathy
Perspective-Taking Subscale (PTS) of the Interpersonal Reactivity Index (Davies, 1983)
Mindfulness
Toronto Alexithymia Scale (Bagby et al., 1994)
<number>

## Slide 17
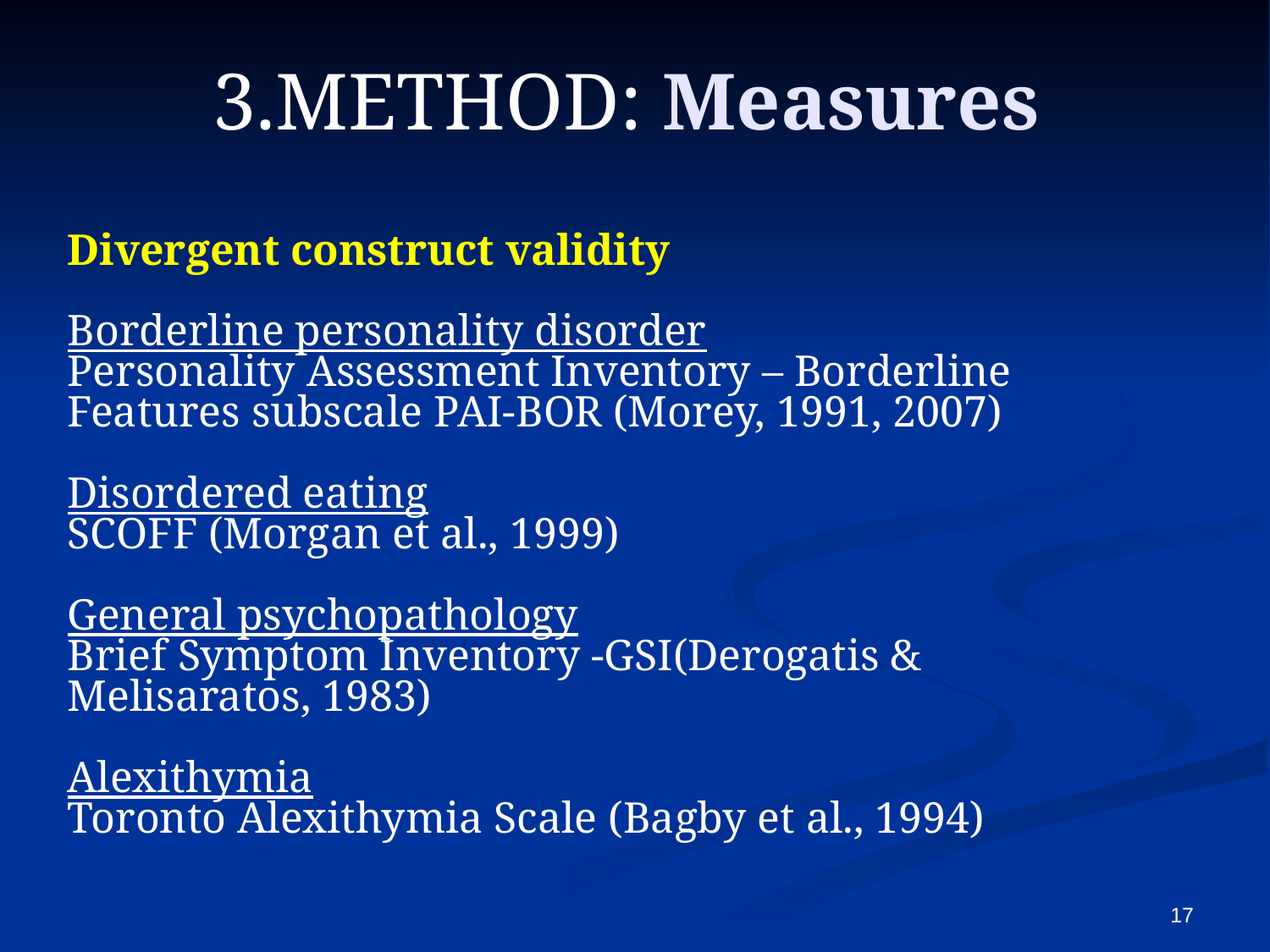

3.METHOD: Measures
Divergent construct validity
Borderline personality disorder
Personality Assessment Inventory – Borderline Features subscale PAI-BOR (Morey, 1991, 2007)
Disordered eating
SCOFF (Morgan et al., 1999)
General psychopathology
Brief Symptom Inventory -GSI(Derogatis & Melisaratos, 1983)
Alexithymia
Toronto Alexithymia Scale (Bagby et al., 1994)
<number>

## Slide 18
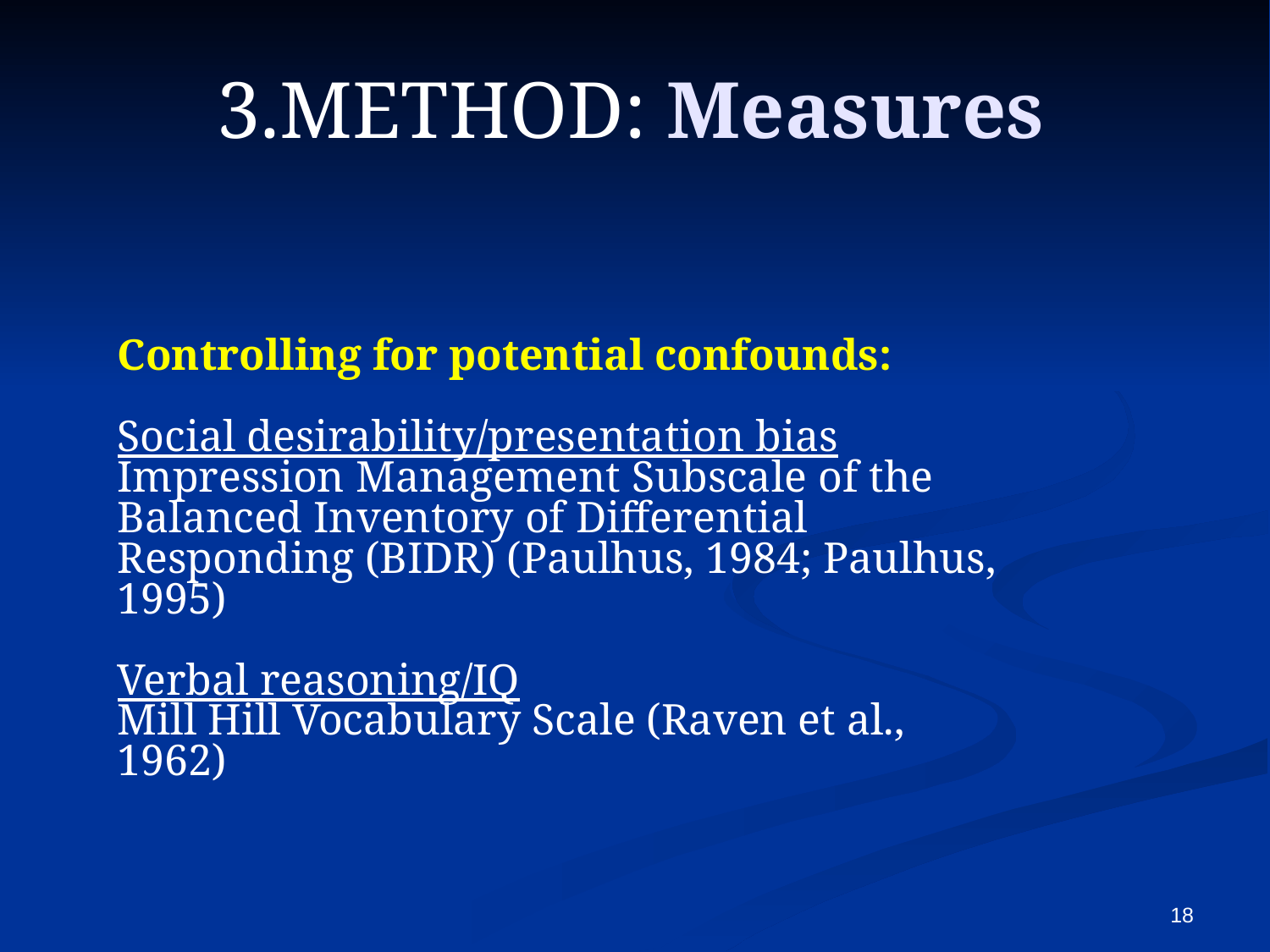

3.METHOD: Measures
Controlling for potential confounds:
Social desirability/presentation bias
Impression Management Subscale of the Balanced Inventory of Differential Responding (BIDR) (Paulhus, 1984; Paulhus, 1995)
Verbal reasoning/IQ
Mill Hill Vocabulary Scale (Raven et al., 1962)
<number>

## Slide 19
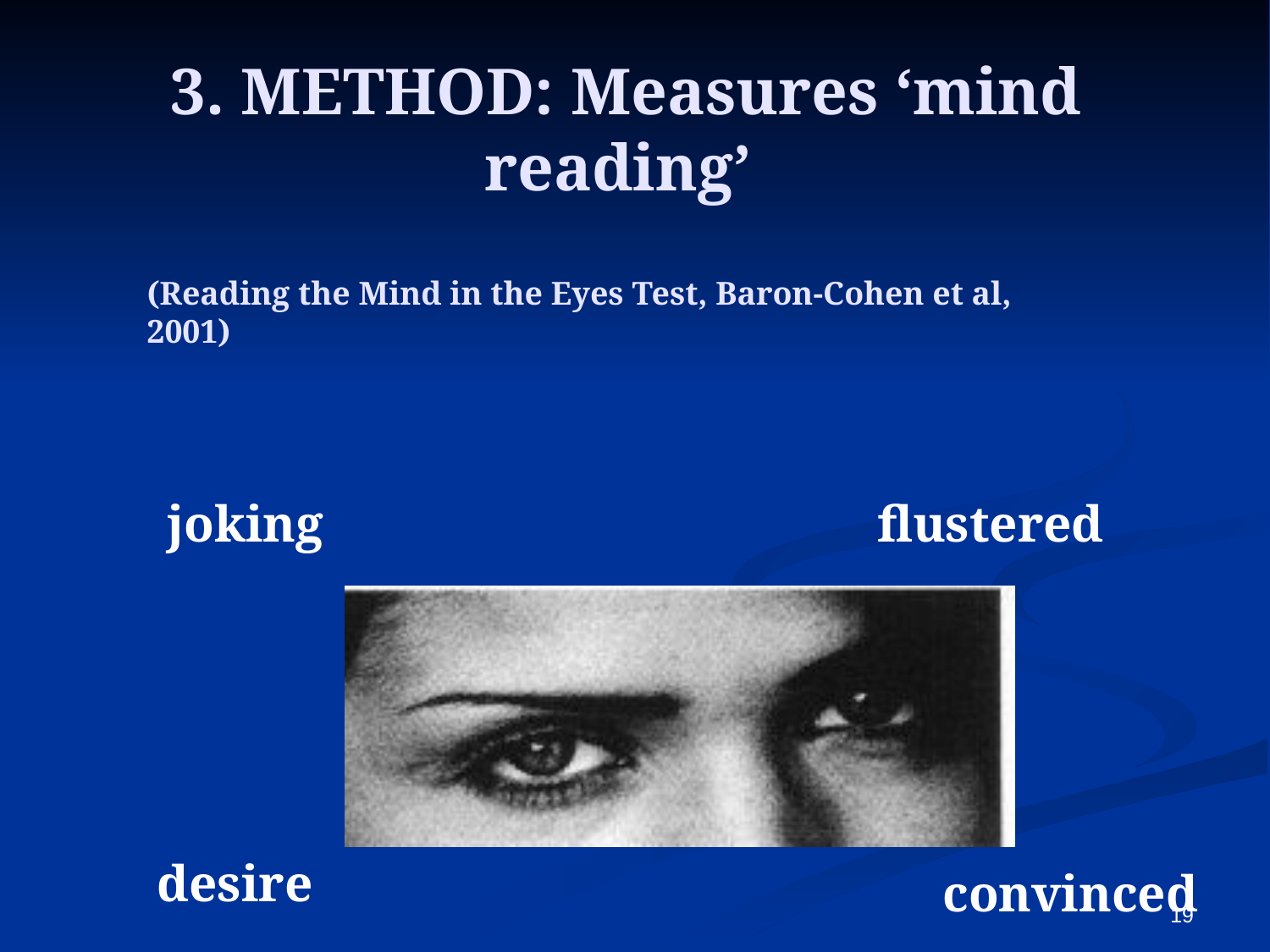

# 3. METHOD: Measures ‘mind reading’
(Reading the Mind in the Eyes Test, Baron-Cohen et al, 2001)
joking
flustered
desire
convinced
<number>

## Slide 20
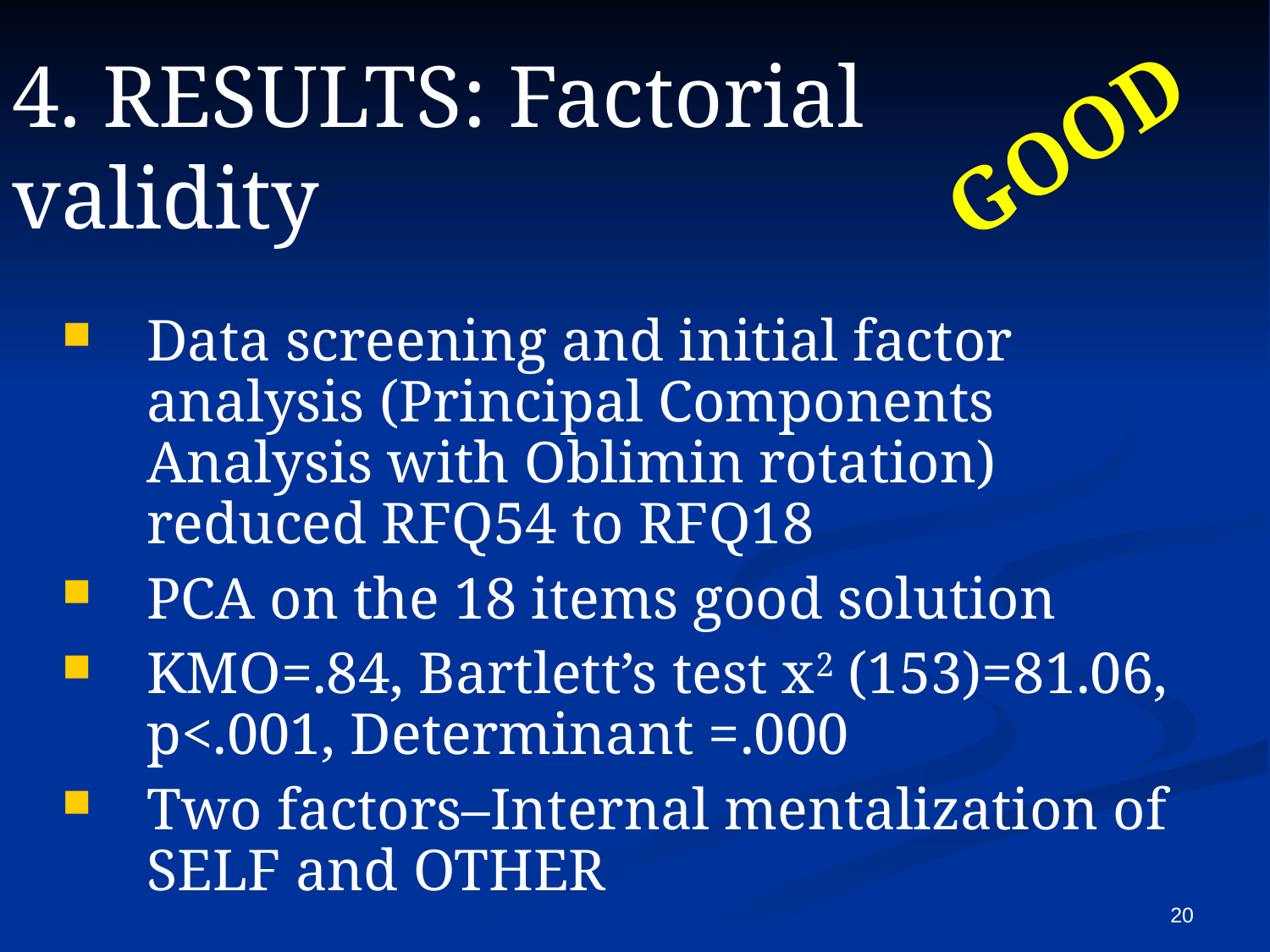

4. RESULTS: Factorial validity
GOOD
Data screening and initial factor analysis (Principal Components Analysis with Oblimin rotation) reduced RFQ54 to RFQ18
PCA on the 18 items good solution
KMO=.84, Bartlett’s test x2 (153)=81.06, p<.001, Determinant =.000
Two factors–Internal mentalization of SELF and OTHER
<number>

## Slide 21
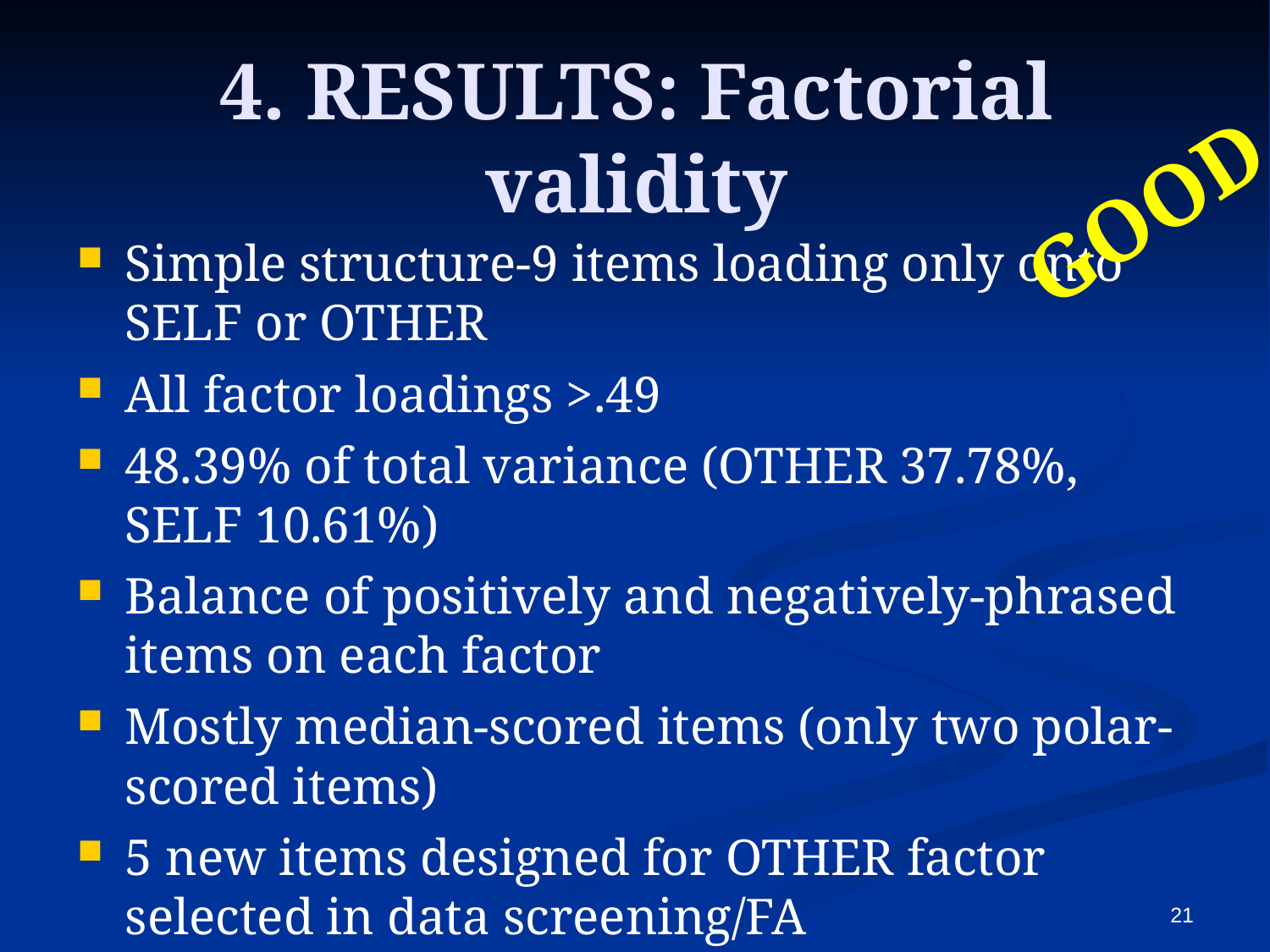

# 4. RESULTS: Factorial validity
GOOD
Simple structure-9 items loading only onto SELF or OTHER
All factor loadings >.49
48.39% of total variance (OTHER 37.78%, SELF 10.61%)
Balance of positively and negatively-phrased items on each factor
Mostly median-scored items (only two polar-scored items)
5 new items designed for OTHER factor selected in data screening/FA
<number>

## Slide 22
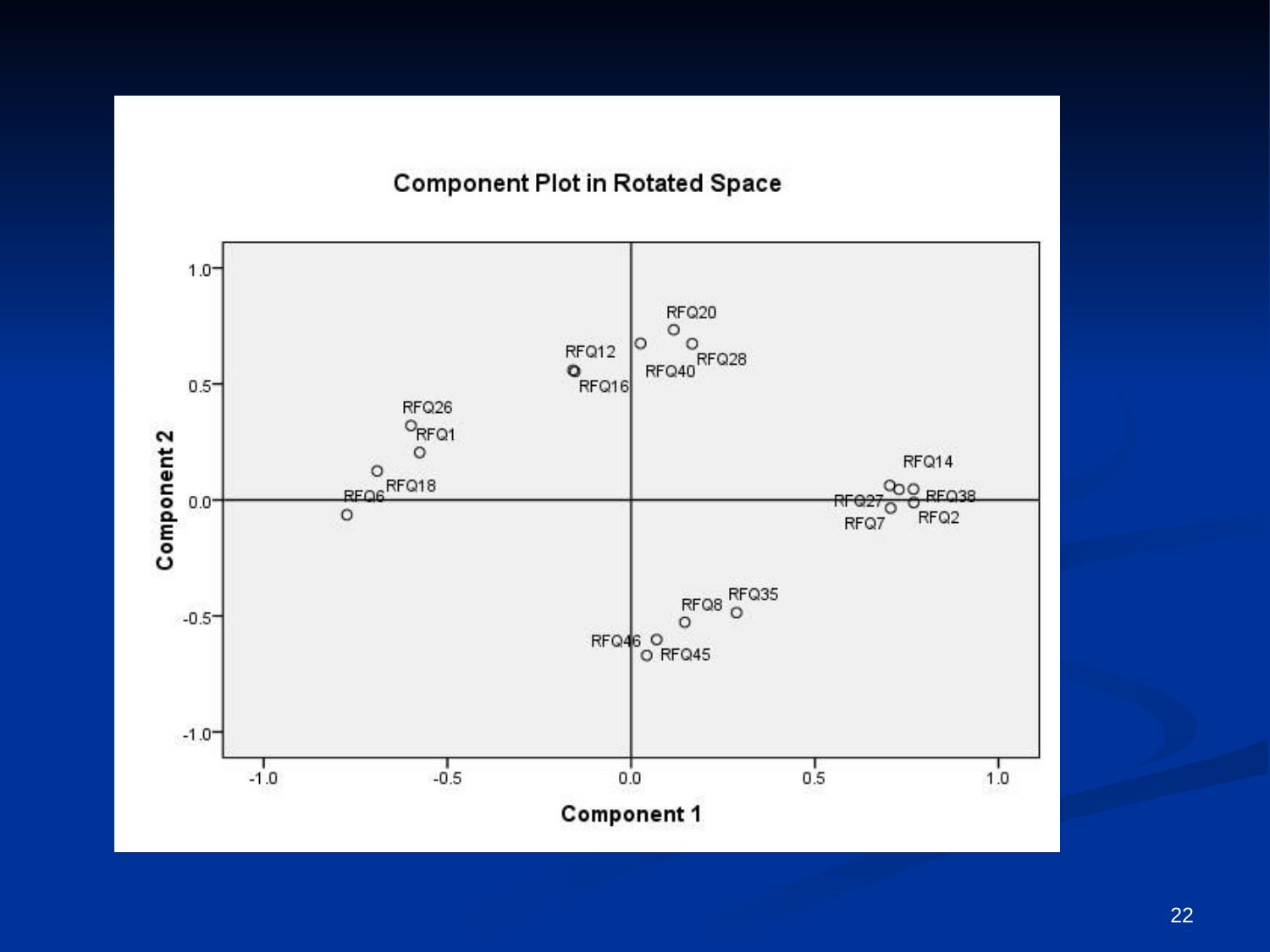

<number>

## Slide 23
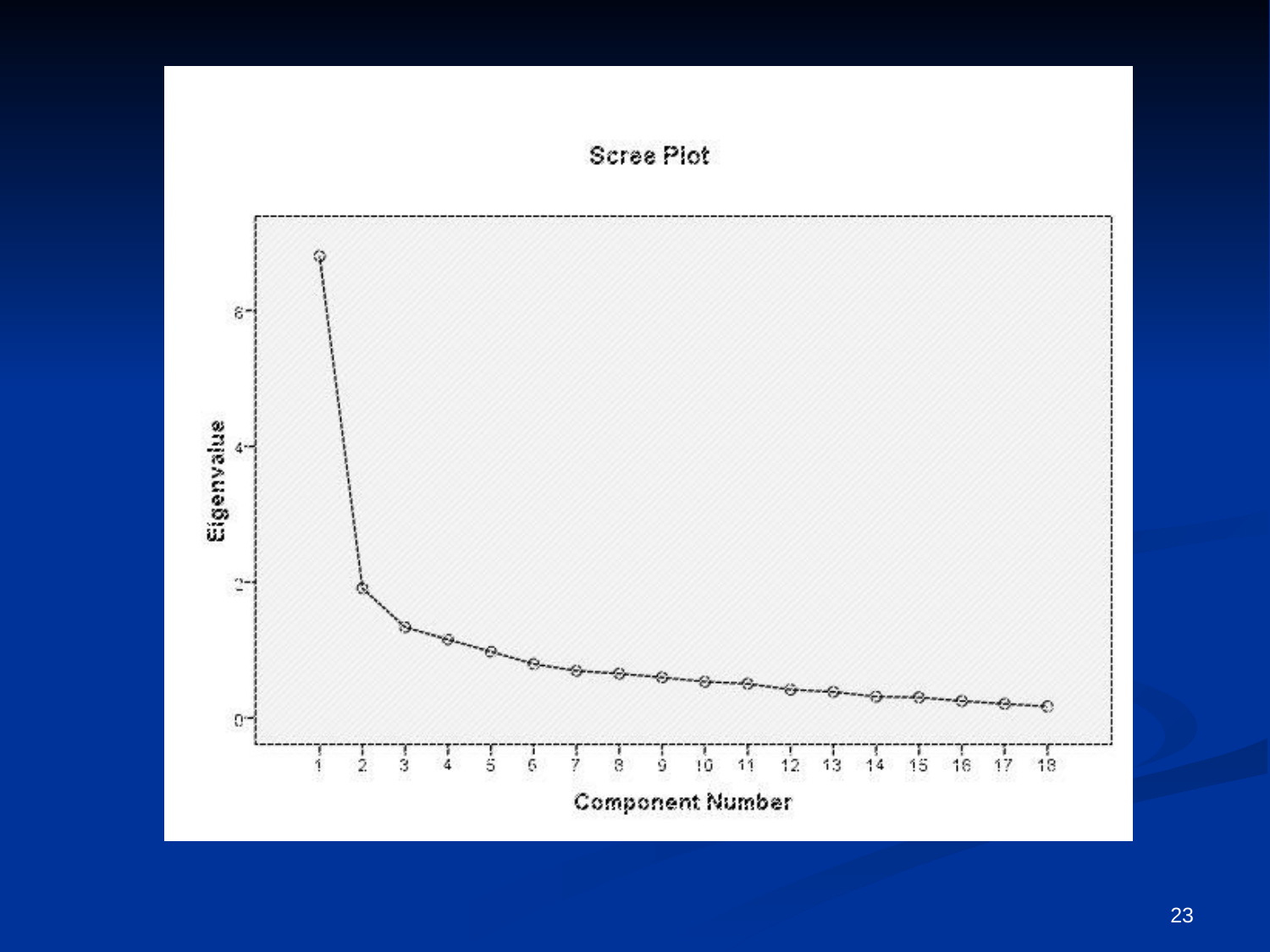

<number>

## Slide 24
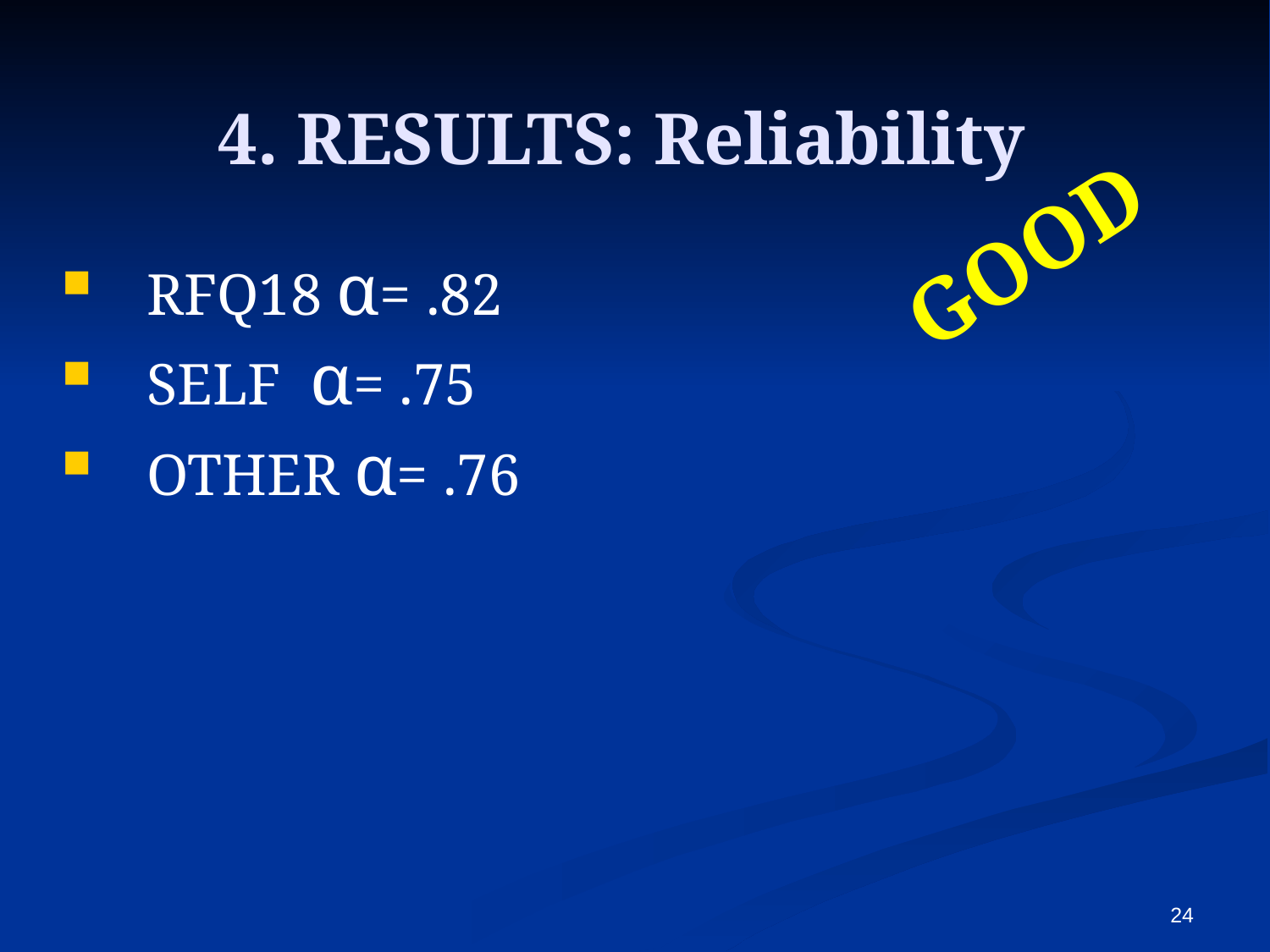

# 4. RESULTS: Reliability
GOOD
RFQ18 α= .82
SELF α= .75
OTHER α= .76
<number>

## Slide 25
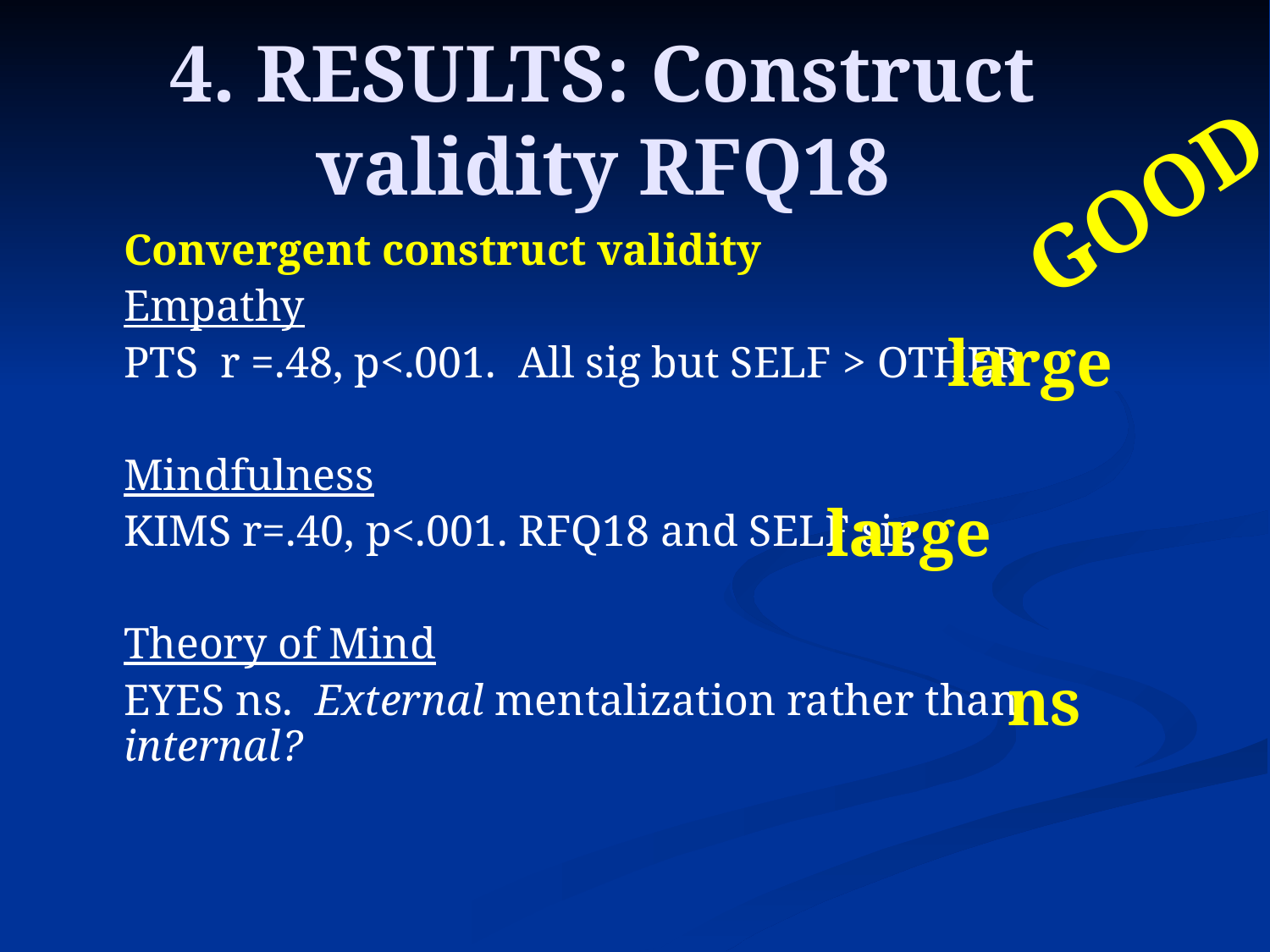

# 4. RESULTS: Construct validity RFQ18
GOOD
Convergent construct validity
Empathy
PTS r =.48, p<.001. All sig but SELF > OTHER
Mindfulness
KIMS r=.40, p<.001. RFQ18 and SELF sig
Theory of Mind
EYES ns. External mentalization rather than internal?
large
large
ns

## Slide 26
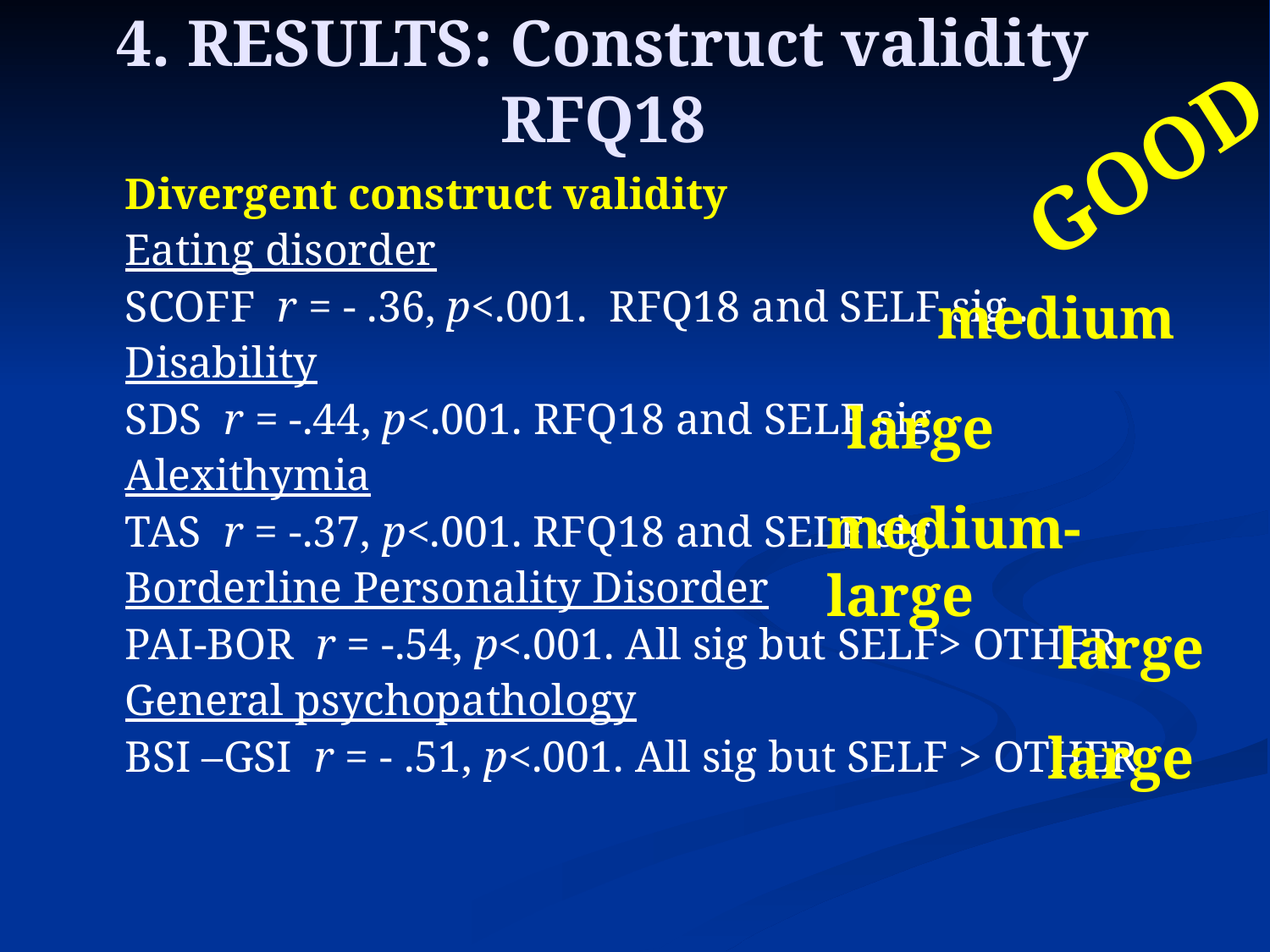

# 4. RESULTS: Construct validity RFQ18
GOOD
Divergent construct validity
Eating disorder
SCOFF r = - .36, p<.001. RFQ18 and SELF sig .
Disability
SDS r = -.44, p<.001. RFQ18 and SELF sig
Alexithymia
TAS r = -.37, p<.001. RFQ18 and SELF sig
Borderline Personality Disorder
PAI-BOR r = -.54, p<.001. All sig but SELF> OTHER
General psychopathology
BSI –GSI r = - .51, p<.001. All sig but SELF > OTHER
medium
large
medium-large
large
large

## Slide 27
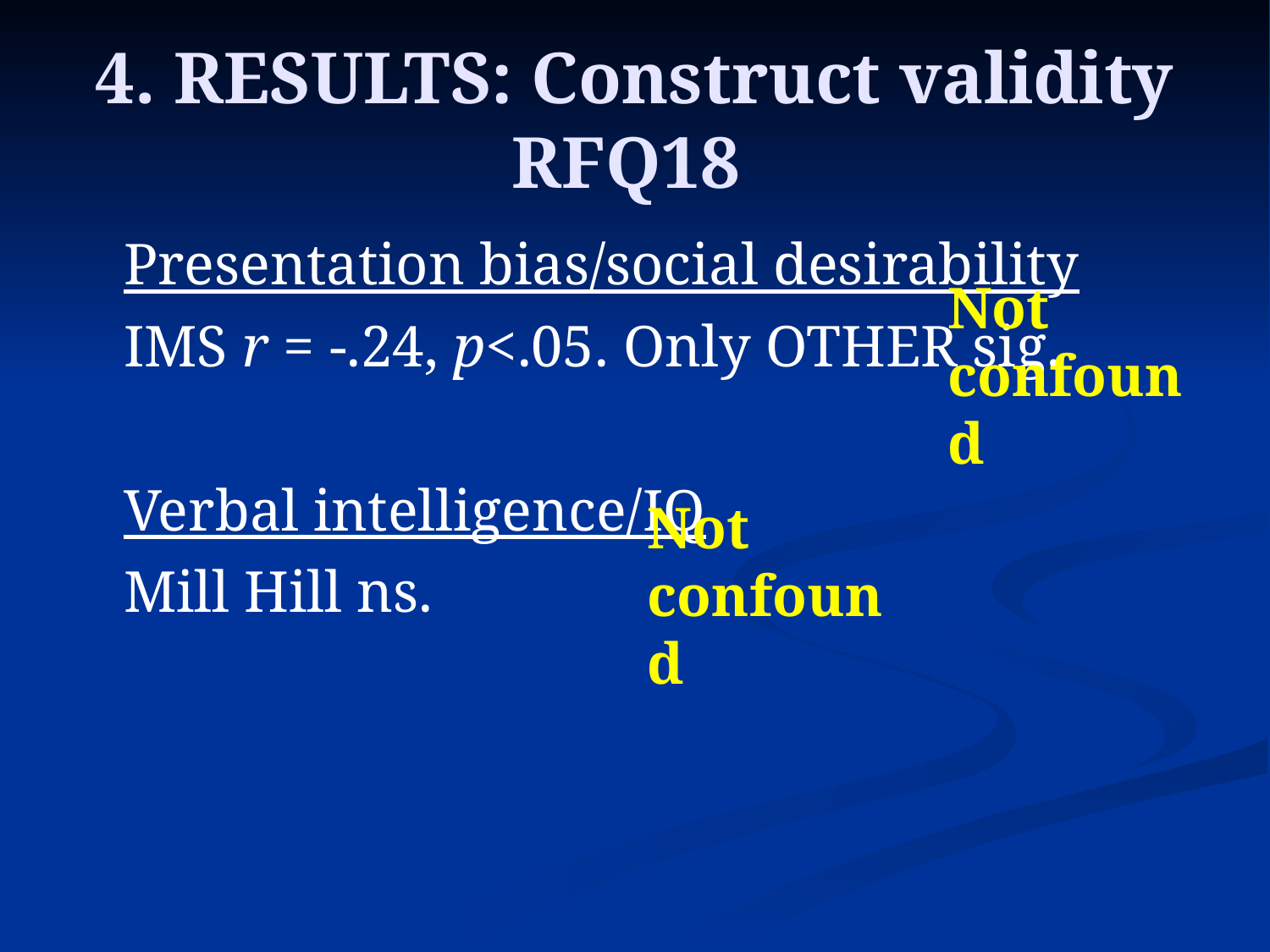

# 4. RESULTS: Construct validity RFQ18
Presentation bias/social desirability
IMS r = -.24, p<.05. Only OTHER sig.
Verbal intelligence/IQ
Mill Hill ns.
Not confound
Not confound

## Slide 28
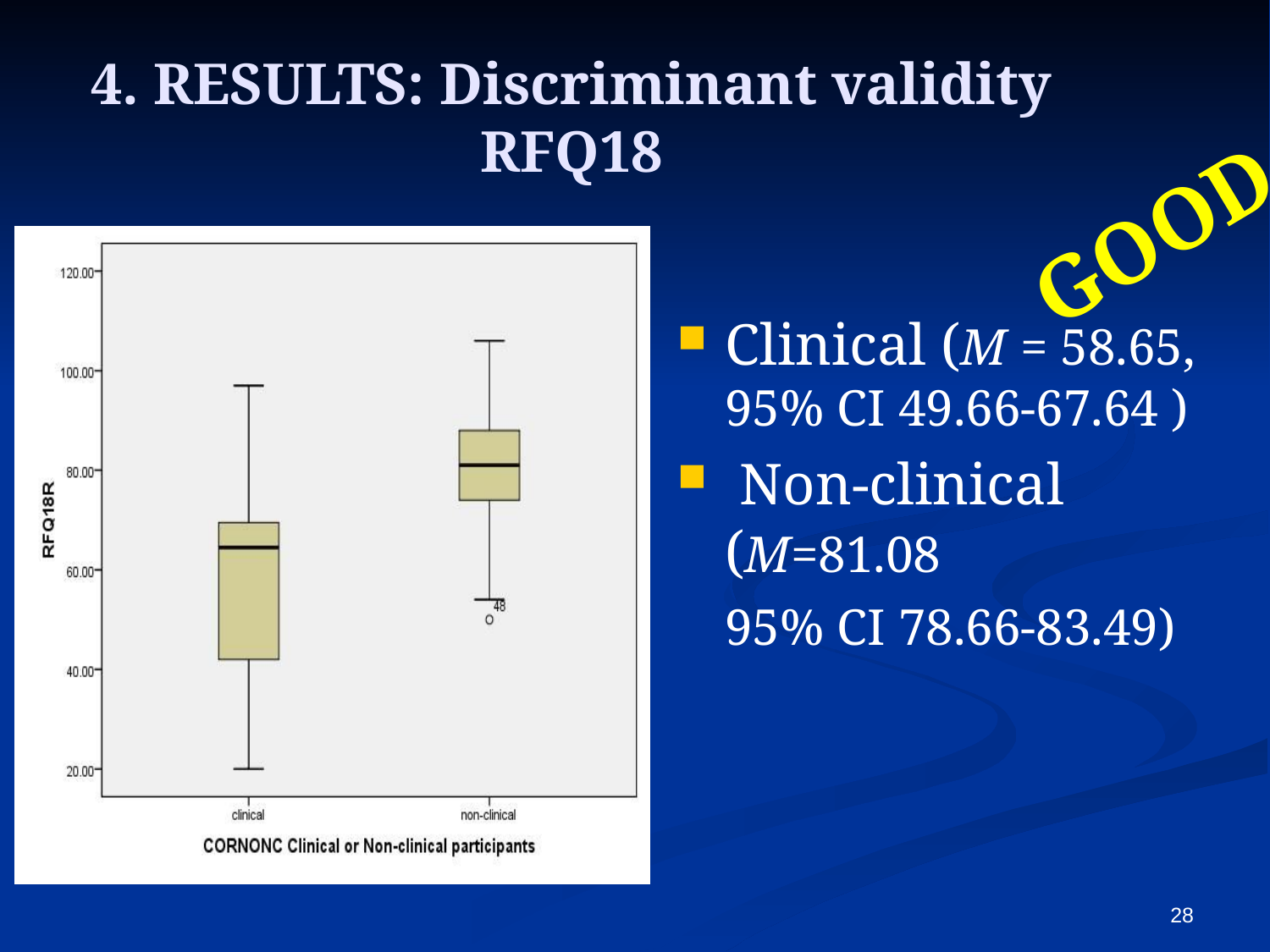

# 4. RESULTS: Discriminant validity RFQ18
GOOD
Clinical (M = 58.65, 95% CI 49.66-67.64 )
 Non-clinical (M=81.08
95% CI 78.66-83.49)
<number>

## Slide 29
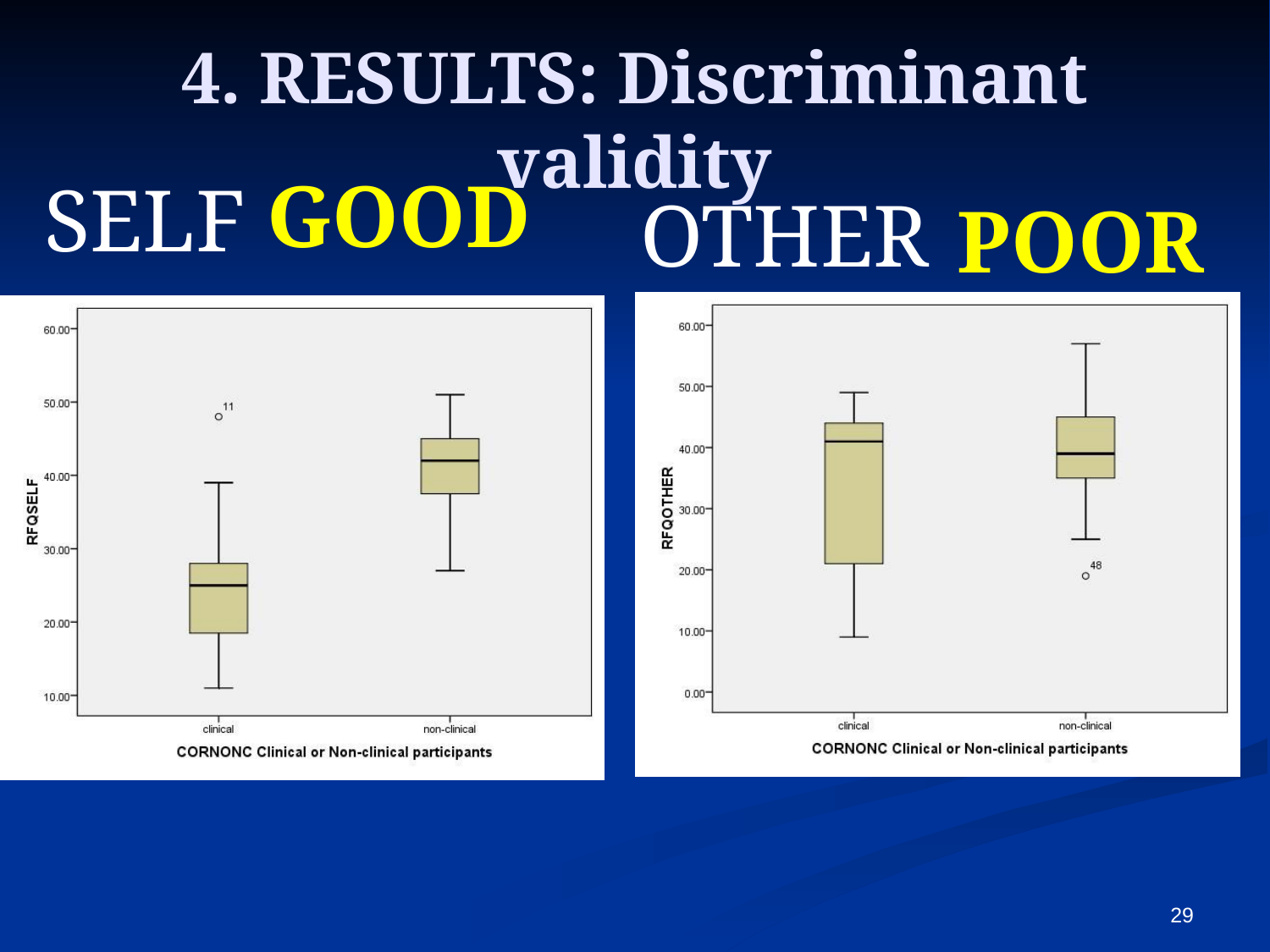

# 4. RESULTS: Discriminant validity
GOOD
SELF
OTHER
POOR
<number>

## Slide 30
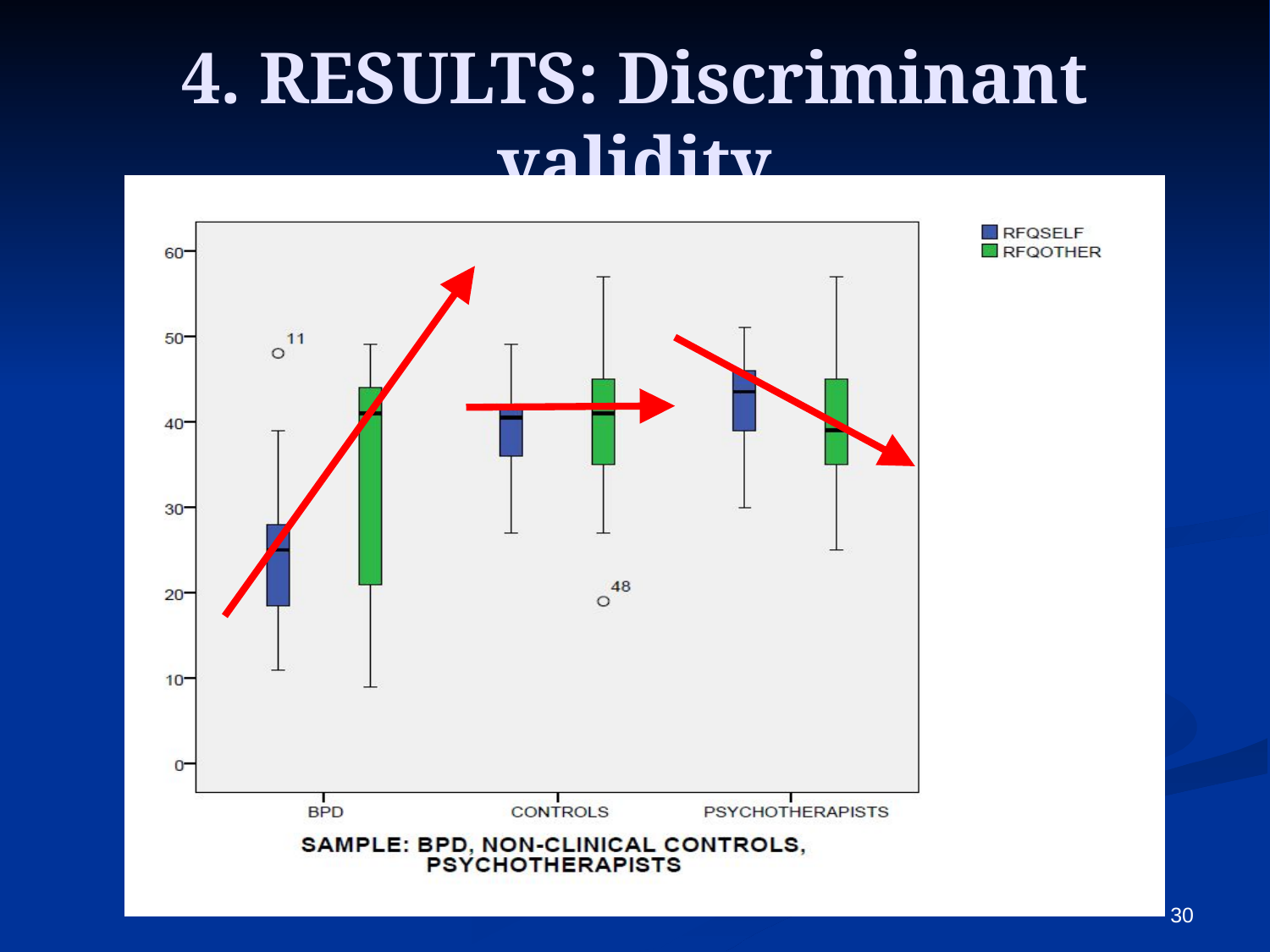

# 4. RESULTS: Discriminant validity
<number>

## Slide 31
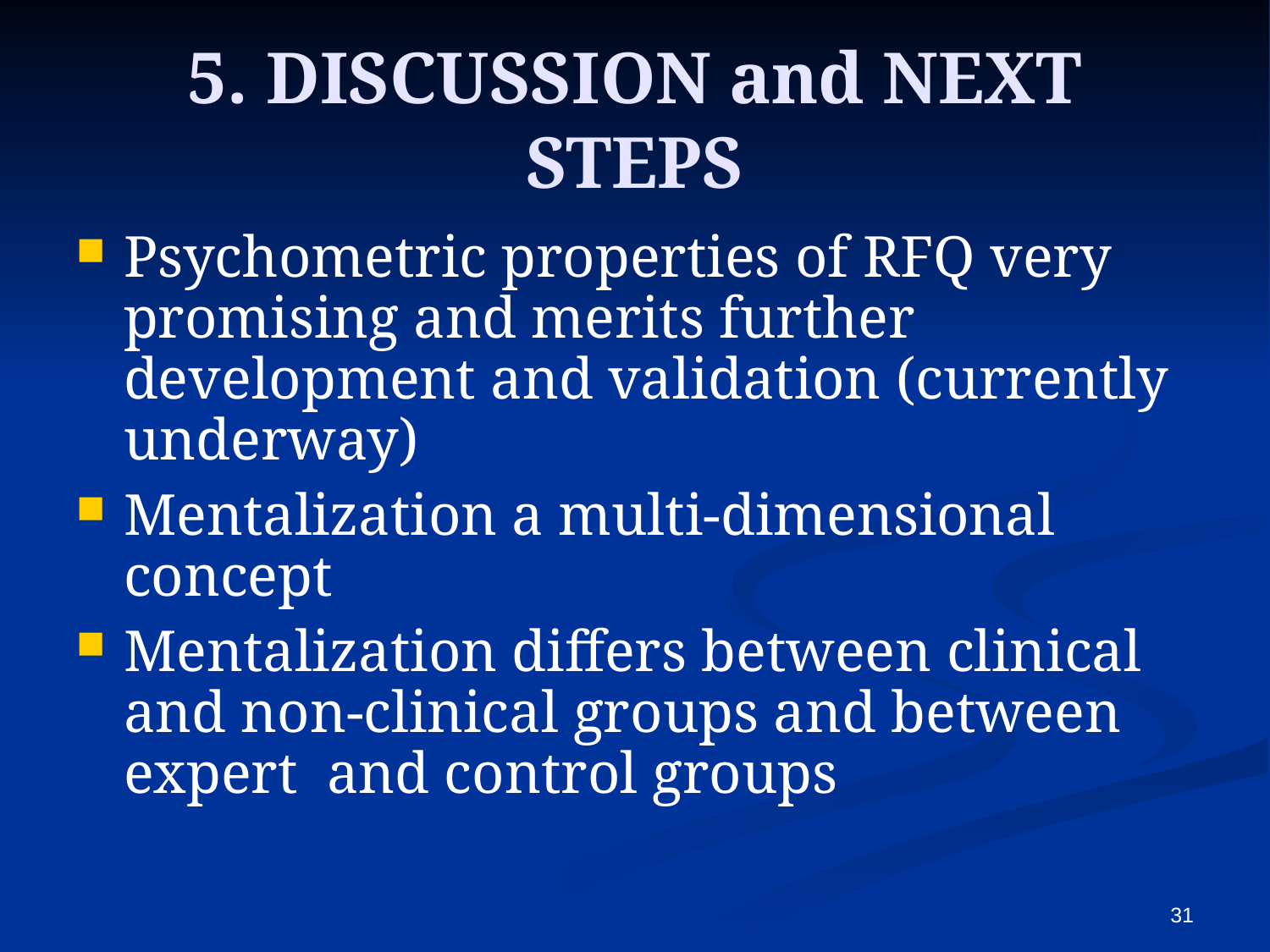

# 5. DISCUSSION and NEXT STEPS
Psychometric properties of RFQ very promising and merits further development and validation (currently underway)
Mentalization a multi-dimensional concept
Mentalization differs between clinical and non-clinical groups and between expert and control groups
<number>

## Slide 32
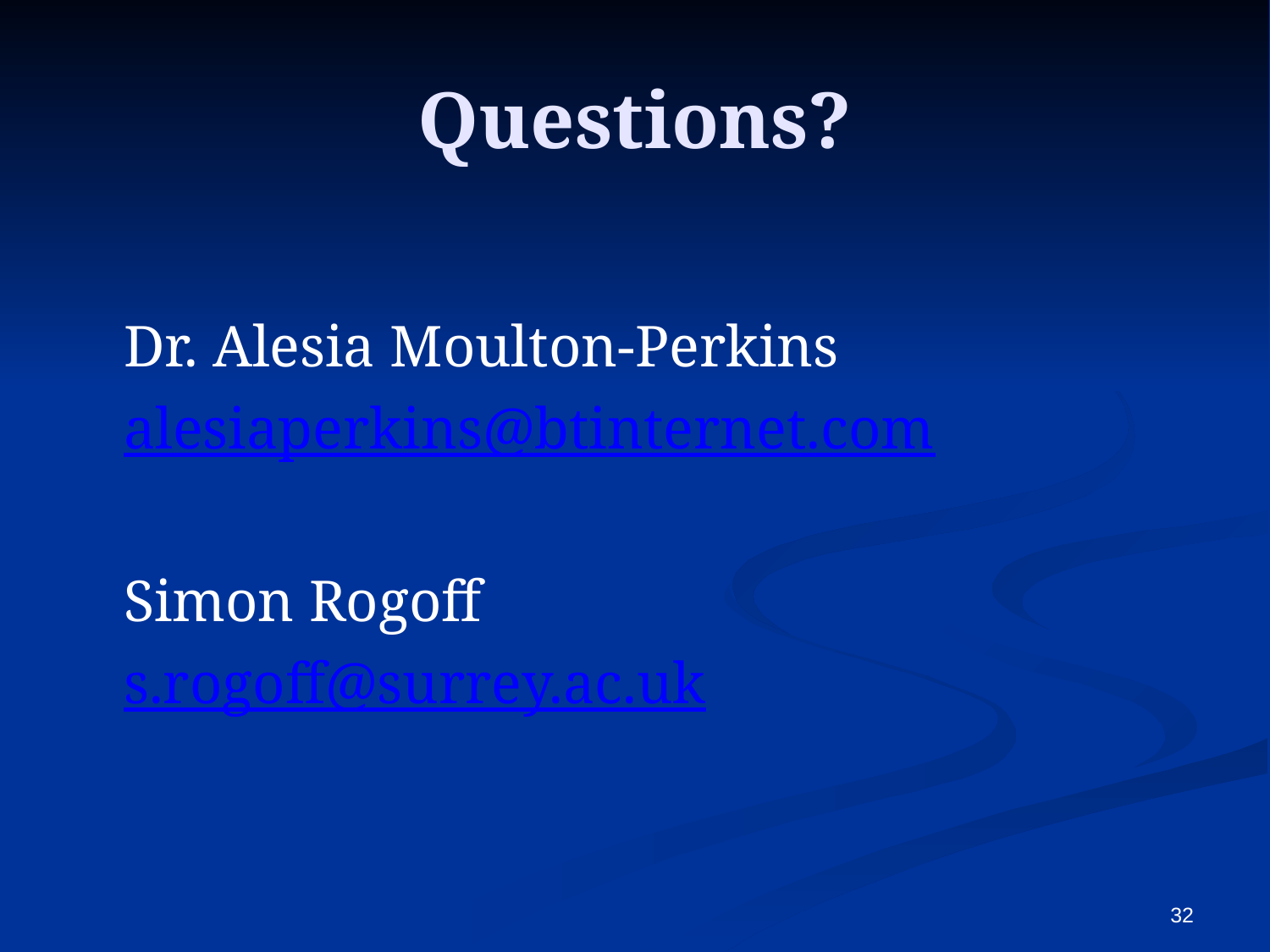

# Questions?
Dr. Alesia Moulton-Perkins
alesiaperkins@btinternet.com
Simon Rogoff
s.rogoff@surrey.ac.uk
<number>

## Slide 33
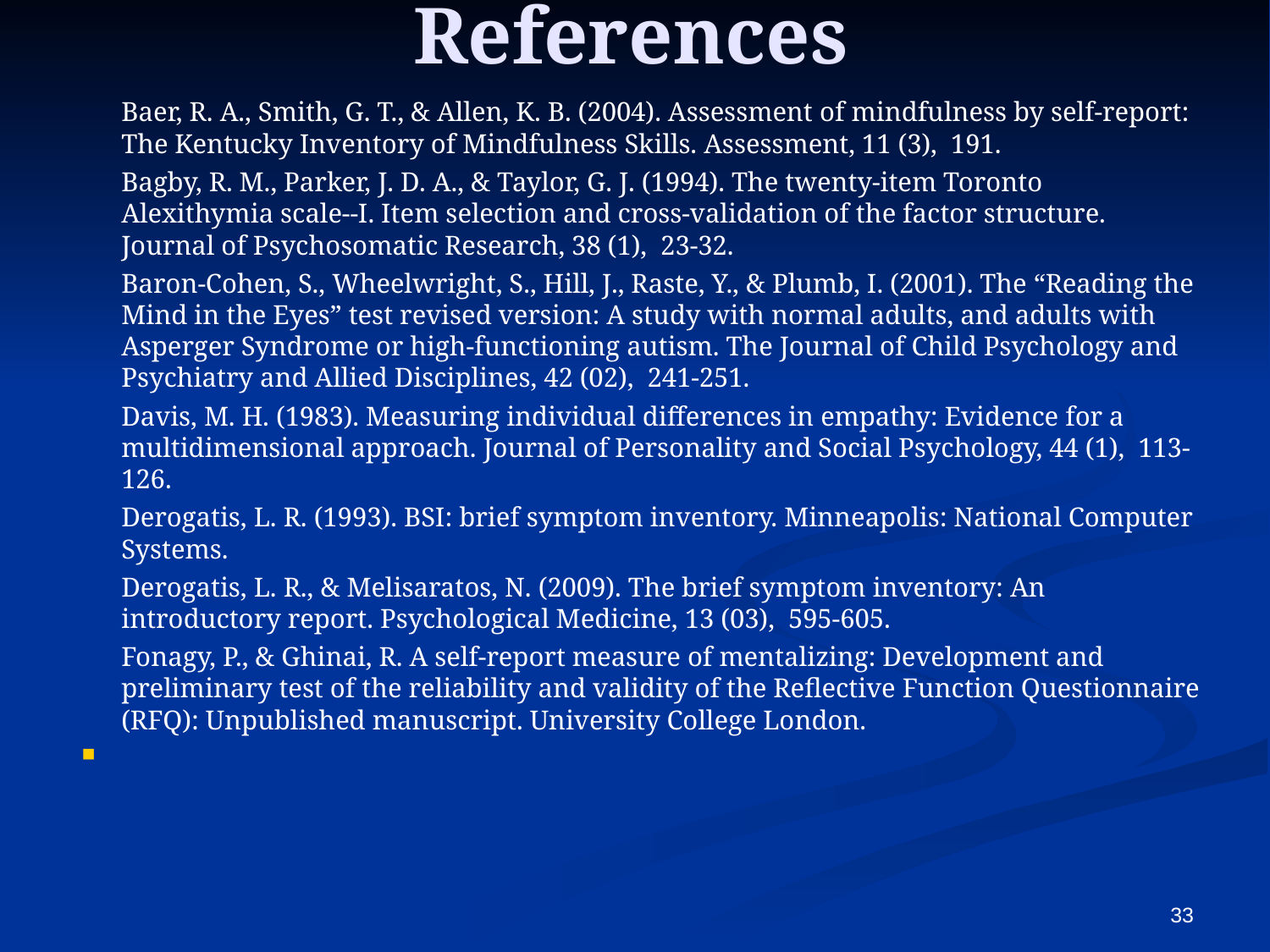

# References
	Baer, R. A., Smith, G. T., & Allen, K. B. (2004). Assessment of mindfulness by self-report: The Kentucky Inventory of Mindfulness Skills. Assessment, 11 (3), 191.
	Bagby, R. M., Parker, J. D. A., & Taylor, G. J. (1994). The twenty-item Toronto Alexithymia scale--I. Item selection and cross-validation of the factor structure. Journal of Psychosomatic Research, 38 (1), 23-32.
	Baron-Cohen, S., Wheelwright, S., Hill, J., Raste, Y., & Plumb, I. (2001). The “Reading the Mind in the Eyes” test revised version: A study with normal adults, and adults with Asperger Syndrome or high-functioning autism. The Journal of Child Psychology and Psychiatry and Allied Disciplines, 42 (02), 241-251.
	Davis, M. H. (1983). Measuring individual differences in empathy: Evidence for a multidimensional approach. Journal of Personality and Social Psychology, 44 (1), 113-126.
	Derogatis, L. R. (1993). BSI: brief symptom inventory. Minneapolis: National Computer Systems.
	Derogatis, L. R., & Melisaratos, N. (2009). The brief symptom inventory: An introductory report. Psychological Medicine, 13 (03), 595-605.
	Fonagy, P., & Ghinai, R. A self-report measure of mentalizing: Development and preliminary test of the reliability and validity of the Reflective Function Questionnaire (RFQ): Unpublished manuscript. University College London.
<number>

## Slide 34
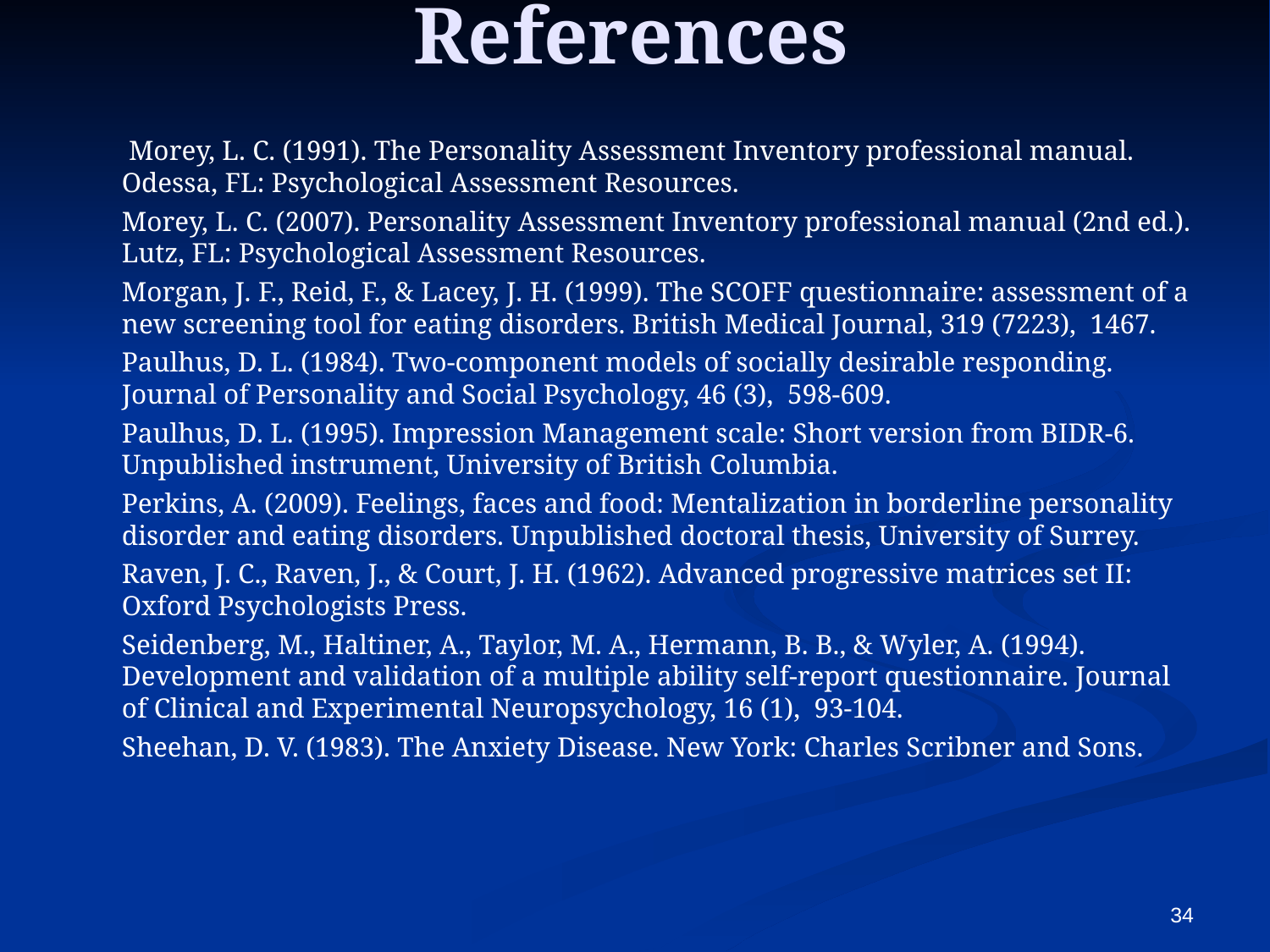

# References
	 Morey, L. C. (1991). The Personality Assessment Inventory professional manual. Odessa, FL: Psychological Assessment Resources.
	Morey, L. C. (2007). Personality Assessment Inventory professional manual (2nd ed.). Lutz, FL: Psychological Assessment Resources.
	Morgan, J. F., Reid, F., & Lacey, J. H. (1999). The SCOFF questionnaire: assessment of a new screening tool for eating disorders. British Medical Journal, 319 (7223), 1467.
	Paulhus, D. L. (1984). Two-component models of socially desirable responding. Journal of Personality and Social Psychology, 46 (3), 598-609.
	Paulhus, D. L. (1995). Impression Management scale: Short version from BIDR-6. Unpublished instrument, University of British Columbia.
	Perkins, A. (2009). Feelings, faces and food: Mentalization in borderline personality disorder and eating disorders. Unpublished doctoral thesis, University of Surrey.
	Raven, J. C., Raven, J., & Court, J. H. (1962). Advanced progressive matrices set II: Oxford Psychologists Press.
	Seidenberg, M., Haltiner, A., Taylor, M. A., Hermann, B. B., & Wyler, A. (1994). Development and validation of a multiple ability self-report questionnaire. Journal of Clinical and Experimental Neuropsychology, 16 (1), 93-104.
	Sheehan, D. V. (1983). The Anxiety Disease. New York: Charles Scribner and Sons.
<number>

## Slide 35
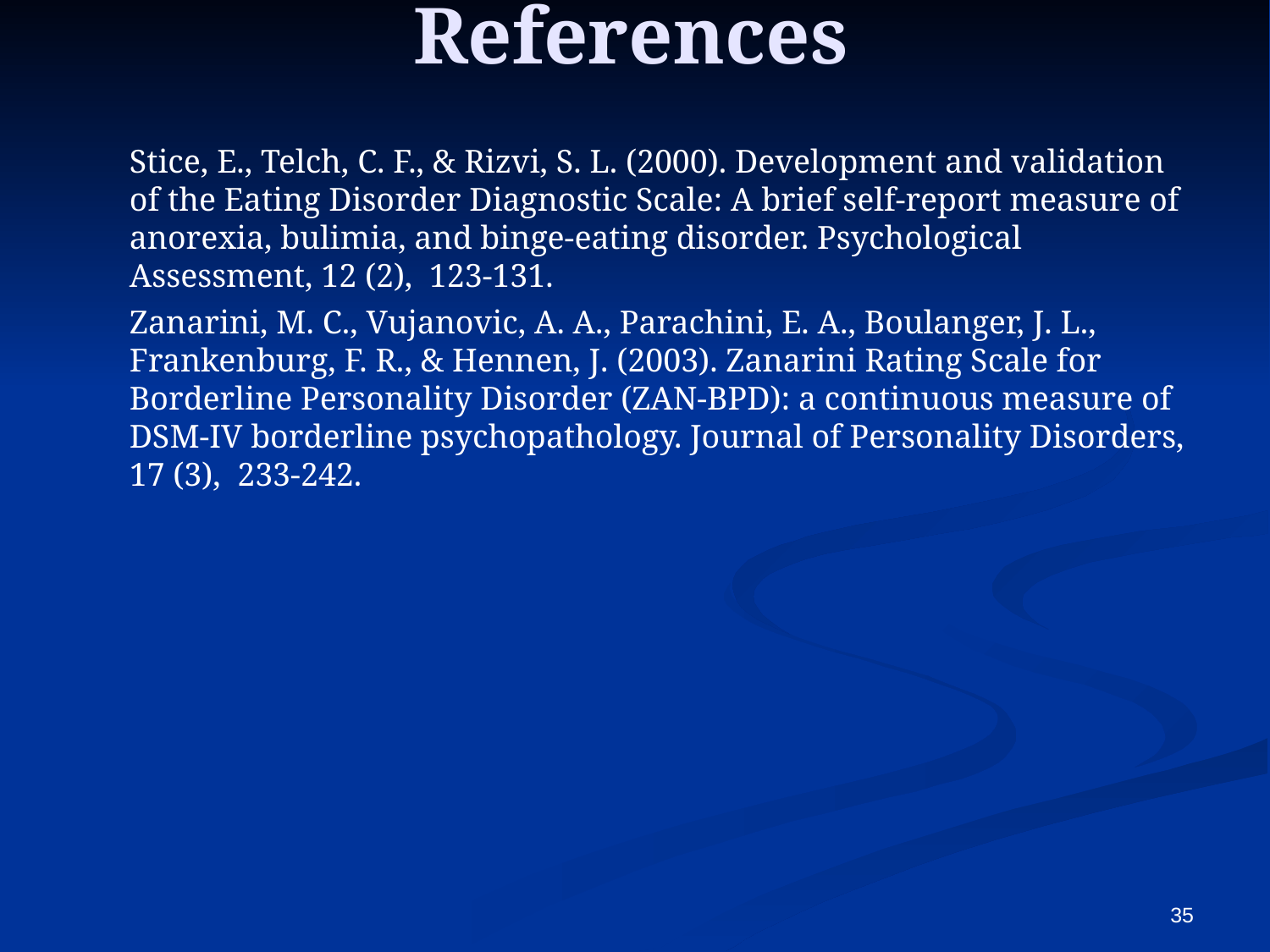

# References
	Stice, E., Telch, C. F., & Rizvi, S. L. (2000). Development and validation of the Eating Disorder Diagnostic Scale: A brief self-report measure of anorexia, bulimia, and binge-eating disorder. Psychological Assessment, 12 (2), 123-131.
	Zanarini, M. C., Vujanovic, A. A., Parachini, E. A., Boulanger, J. L., Frankenburg, F. R., & Hennen, J. (2003). Zanarini Rating Scale for Borderline Personality Disorder (ZAN-BPD): a continuous measure of DSM-IV borderline psychopathology. Journal of Personality Disorders, 17 (3), 233-242.
<number>
