# Supplementary material for: ‘Rich’ and ‘poor’ in mentalizing: Do expert mentalizers exist?
Source: PLoS One. 2021 Oct 25;16(10):e0259030. doi: 10.1371/journal.pone.0259030 (PMC8544847; doi:10.1371/journal.pone.0259030)
Supplement: S1 Text — (PDF) [file pone.0259030.s005.pdf]

S1 Text. RFQ18 self and other subscale items and RFQ54

RFQ18 self and other subscale items from RFQ54

RFQ-self items

8. I know exactly what my close friends are thinking.
12. I often get confused about what I am feeling.
16. I don't always know why I do what I do.
20. When I get angry I say things without really knowing why I am saying them.
28. Strong feelings often cloud my thinking.
35. I trust my feelings.
40. If I feel insecure I can behave in ways that put others' backs up.
45. I pay attention to my feelings.
46. In an argument, I keep the other person's point of view in mind.

RFQ-other items

1. People's thoughts are a mystery to me.
2. It's easy for me to figure out what someone else is thinking or feeling.
6. It takes me a long time to understand other people's thoughts and feelings.
7. I know exactly what my close friends are thinking.
14. Understanding what's on someone else's mind is never difficult for me.
18. It's really hard for me to figure out what goes on in other people's heads.
26. Other people's thoughts and feelings are confusing to me.
27. I can mostly predict what someone else will do.
38. I am a good mind reader.

S1 Text. RFQ18 self and other subscale items and RFQ54

RFQ54

Please work through the next 54 statements. Choose the one response that you feel describes you most clearly. Choose any number between 1 and 7 to say how much you disagree or agree with the statement. Strongly disagree is 1. Strongly agree is 7. Neither agree nor disagree is 4.

Do not think too much about it - your initial responses are usually the best. Thank you.

	Strongly DISAGREE →					Strongly AGREE
1. People's thoughts are a mystery to me.	1	2	3	4	5	6	7
2. It's easy for me to figure out what someone else is thinking or feeling.	1	2	3	4	5	6	7
3. My picture of my parents changes as I change.	1	2	3	4	5	6	7
4. I worry a great deal about what people are thinking and feeling.	1	2	3	4	5	6	7
5. I pay attention to the impact of my actions on others' feelings.	1	2	3	4	5	6	7
6. It takes me a long time to understand other people's thoughts and feelings.	1	2	3	4	5	6	7
7. I know exactly what my close friends are thinking.	1	2	3	4	5	6	7
8. I always know what I feel.	1	2	3	4	5	6	7
9. How I feel can easily affect how I understand someone else's behaviour.	1	2	3	4	5	6	7
10. I can tell how someone is feeling by looking at their eyes.	1	2	3	4	5	6	7
11. I realise that I can sometimes misunderstand my best friends' reactions.	1	2	3	4	5	6	7
12. I often get confused about what I am feeling.	1	2	3	4	5	6	7
13. I wonder what my dreams mean.	1	2	3	4	5	6	7
14. Understanding what's on someone else's mind is never difficult for me.	1	2	3	4	5	6	7
15. I believe that my parents' behaviour towards me should not be explained by how they were brought up.	1	2	3	4	5	6	7
16. I don't always know why I do what I do.	1	2	3	4	5	6	7
17. I have noticed that people often give advice to others that they actually wish to follow themselves.	1	2	3	4	5	6	7
18. It's really hard for me to figure out what goes on in other people's heads.	1	2	3	4	5	6	7

S1 Text. RFQ18 self and other subscale items and RFQ54

	Strongly DISAGREE			→			Strongly AGREE
19. Other people tell me I'm a good listener.	1	2	3	4	5	6	7
20. When I get angry I say things without really knowing why I am saying them.	1	2	3	4	5	6	7
21. I'm often curious about the meaning behind others' actions.	1	2	3	4	5	6	7
22. I really struggle to make sense of other people's feelings.	1	2	3	4	5	6	7
23. I often have to force people to do what I want them to do.	1	2	3	4	5	6	7
24. Those close to me often seem to find it difficult to understand why I do things.	1	2	3	4	5	6	7
25. I feel that, if I am not careful, I could intrude into another person's life.	1	2	3	4	5	6	7
26. Other people's thoughts and feelings are confusing to me.	1	2	3	4	5	6	7
27. I can mostly predict what someone else will do.	1	2	3	4	5	6	7
28. Strong feelings often cloud my thinking.	1	2	3	4	5	6	7
29. In order to know exactly how someone is feeling, I have found that I need to ask them.	1	2	3	4	5	6	7
30. My intuition about a person is hardly ever wrong.	1	2	3	4	5	6	7
31. I believe that people can see a situation very differently based on their own beliefs and experiences.	1	2	3	4	5	6	7
32. Sometimes I find myself saying things and I have no idea why I said them.	1	2	3	4	5	6	7
33. I like to think about the reasons behind my actions.	1	2	3	4	5	6	7
34. I normally have a good idea of what is on other people's minds.	1	2	3	4	5	6	7
35. I trust my feelings.	1	2	3	4	5	6	7
36. When I get angry I say things that I later regret.	1	2	3	4	5	6	7
37. I get confused when people talk about their feelings.	1	2	3	4	5	6	7
38. I am a good mind reader.	1	2	3	4	5	6	7
39. I frequently feel that my mind is empty.	1	2	3	4	5	6	7

S1 Text. RFQ18 self and other subscale items and RFQ54

	Strongly DISAGREE → Strongly AGREE						
40. If I feel insecure I can behave in ways that put others' backs up.	1	2	3	4	5	6	7
41. I find it difficult to see other people's points of view.	1	2	3	4	5	6	7
42. I usually know exactly what other people are thinking.	1	2	3	4	5	6	7
43. I anticipate that my feelings might change even about something I feel strongly about.	1	2	3	4	5	6	7
44. Sometimes I do things without really knowing why.	1	2	3	4	5	6	7
45. I pay attention to my feelings.	1	2	3	4	5	6	7
46. In an argument, I keep the other person's point of view in mind.	1	2	3	4	5	6	7
47. My gut feeling about what someone else is thinking is usually very accurate.	1	2	3	4	5	6	7
48. Understanding the reasons for people's actions helps me to forgive them.	1	2	3	4	5	6	7
49. I believe that there is no RIGHT way of seeing any situation.	1	2	3	4	5	6	7
50. I am better guided by reason than by my gut.	1	2	3	4	5	6	7
51. I can't remember much about when I was a child.	1	2	3	4	5	6	7
52. I believe there's no point trying to guess what's on someone else's mind.	1	2	3	4	5	6	7
53. For me actions speak louder than words.	1	2	3	4	5	6	7
54. I believe other people are too confusing to bother figuring out.	1	2	3	4	5	6	7
