# Supplementary material for: ‘Rich’ and ‘poor’ in mentalizing: Do expert mentalizers exist?
Source: PLoS One. 2021 Oct 25;16(10):e0259030. doi: 10.1371/journal.pone.0259030 (PMC8544847; doi:10.1371/journal.pone.0259030)
Supplement: S2 Text — (PDF) [file pone.0259030.s006.pdf]

S2 Text. Participants information sheet

Clinical Psychology Programme
Department of Psychology
University of Surrey
Guildford, Surrey
GU2 7XH
Tel: 01483 689441

Participant Information Sheet

'Feelings & Faces'

Dear Participant,

You are being invited to take part in a research study. Your decision to take part in this study is entirely voluntary and you are under no obligation to do so and your care will not be affected in any way. Before you decide whether or not to take part in this study it is important for you to understand why the research is being done and what it will involve. Please take time to read the following information carefully and discuss it with others if you wish. If there is anything that is not clear, or if you would like more information please ask your clinician. After you have read through the information take some time to decide whether or not you still wish to take part.

What is the purpose of the research study?
We are looking at the reasons why some people have difficulties with impulsive behaviours and what their feelings are around things like food, relationships, or money. However, some people have none of these problems, or they are very controlled in their behaviour. This study aims to look at how people pick up on what others are thinking and feeling, as well as how people think about their own thoughts and feelings. Greater knowledge in this area will help in designing better psychological therapies for people who have impulsive behaviours or strong feelings they find it difficult to deal with.

Why have I been chosen to take part in this study?
All sorts of people will be taking part in this study. We want to include a range of different people so we can see how things like interpreting other's facial expressions differ among people.

Who is organising the study?
I am Alesia Perkins, a clinical psychologist. This study forms part of a research study at University College London. We are also working with researchers at the University of Surrey.

Who has reviewed the study?
All research in the NHS is looked at by an independent group of people, called a Research Ethics Committee to protect your safety, rights, wellbeing and dignity. This study has been reviewed and given favourable opinion by the London & South East Coast Research Ethics Committee.

What will happen to me if I take part?
If you would like to take part, you will be given a questionnaire to fill out. The questionnaire asks a series of questions mainly directed at feelings about yourself and other people. The questionnaire will take about 45 minutes. When you have finished please leave it in the labelled box in the course admin office or post it back directly to the researchers in the Freepost envelope provided.

What are the possible risks and benefits of taking part?
While it is unlikely, it is possible that you might become a little tired from doing the questionnaire. You may experience some upset feelings from answering the questions. If you are interested, your name can be entered into a prize draw. Several £15 vouchers are available for winners. If you want to participate in the prize draw please fill out the consent form with your name and address. The knowledge gained from this study may help improve the treatment of people with strong emotions, impulsiveness and problems with eating.

Confidentiality
All documents relating to the study will be kept in a locked filing cabinet and only the researchers directly involved in the study will have access to them. No-one outside the study will have knowledge of your name. Your answers on the questionnaire itself will not be linked directly to your name. The completed consent form and questionnaires will be kept separately. Data will be stored electronically with a number, not personal names identifying people's answers. Your GP will not be informed of your participation in this study.

What will happen to the results of this study?
I intend publishing the results of this study for scientific purposes. Your identity will not be revealed in any publications.

Who do I speak to if I decide to withdraw from the study or if I want more information?
You are free to decline to enter or to withdraw from the research at any time without having to give a reason. If you have any questions about the study, please contact Alesia Perkins at University College London on 0207 679 1943 or Simon Rogoff at the University of Surrey on 01483 689441.

If you have concerns about disordered eating, impulsive behaviours or thoughts of hurting yourself, please contact your GP or your keyworker/clinician on your team. More general information about participating in research can be obtained from INVOLVE (promoting public involvement in NHS, public health and social care research) www.invo.org.uk, 02380 651 088. Alternatively, you can contact your local Patient Advisory Liaison Service (the number is available through NHS Direct 0845 46 47) who can also help if you want to make a complaint about this research.

To take part, please complete the questionnaire. If you would like to be entered in the prize draw fill in a consent form as well. Put your consent form and questionnaire in the envelope provided. Place it in labelled box in the course admin office or post it back in the Freepost envelope provided. Please keep this information sheet for future reference.

Many thanks, Dr. Alesia Perkins
