# Supplementary material for: ‘Rich’ and ‘poor’ in mentalizing: Do expert mentalizers exist?
Source: PLoS One. 2021 Oct 25;16(10):e0259030. doi: 10.1371/journal.pone.0259030 (PMC8544847; doi:10.1371/journal.pone.0259030)
Supplement: S3 Text — (PDF) [file pone.0259030.s007.pdf]

S3 Text. Questionnaire Pack

Feelings and faces study

The following statements inquire about your thoughts and feelings in a variety of situations. For each item, indicate how well it describes you by circling the appropriate number. Read each item carefully before responding. Answer as honestly as you can. Thank you.

	Does NOT describe me well	→			Describes me VERY well
1. I sometimes find it difficult to see things from the "other guy's point of view".	1	2	3	4	5
2. I try to look at everybody's side of a disagreement before I make a decision.	1	2	3	4	5
3. I sometimes try to understand my friends better by imagining how things look from their perspective.	1	2	3	4	5
4. If I'm sure I'm right about something, I don't waste much time listening to other people's arguments.	1	2	3	4	5
5. I believe that there are two sides to every question and try to look at them both.	1	2	3	4	5
6. When I'm upset at someone, I usually try to "put myself in his shoes" for a while.	1	2	3	4	5
7. Before criticising somebody, I try to imagine how I would feel if I were in their place.	1	2	3	4	5

Please rate each of the following statements using the scale provided. Circle the number that best describes your own opinion of what is generally true for you.

	Never or very rarely true	Rarely true	Sometimes true	Often true	Very often or always true
1. I'm good at finding the words to describe my feelings	1	2	3	4	5
2. I can easily put my beliefs, opinions, and expectations into words	1	2	3	4	5
3. I'm good at thinking of words to express my perceptions, such as how things taste, smell, or sound	1	2	3	4	5
4. It's hard for me to find the words to describe what I'm thinking	1	2	3	4	5
5. I have trouble thinking of the right words to express how I feel about things	1	2	3	4	5
6. When I have a sensation in my body, it's difficult for me to describe it because I can't find the right words	1	2	3	4	5
7. Even when I'm feeling terribly upset, I can find a way to put it into words	1	2	3	4	5
8. My natural tendency is to put my experiences into words	1	2	3	4	5
9. When I do things, my mind wanders off and I'm easily distracted	1	2	3	4	5
10. When I'm doing something, I'm only focused on what I'm doing, nothing else	1	2	3	4	5

Feelings and faces study

	Never or very rarely true	Rarely true	Sometimes true	Often true	Very often or always true
11. I drive on "automatic pilot" without paying attention to what I'm doing	1	2	3	4	5
12. When I'm reading, I focus all my attention on what I'm reading	1	2	3	4	5
13. When I do things, I get totally wrapped up in them and don't think about anything else	1	2	3	4	5
14. I don't pay attention to what I'm doing because I'm daydreaming, worrying, or otherwise distracted	1	2	3	4	5
15. When I'm doing chores, such as cleaning or laundry, I tend to daydream or think of other things	1	2	3	4	5
16. I tend to do several things at once rather than focusing on one thing at a time	1	2	3	4	5
17. When I'm working on something, part of my mind is occupied with other topics, such as what I'll be doing later, or things I'd rather be doing	1	2	3	4	5
18. I get completely absorbed in what I'm doing, so that all my attention is focused on it	1	2	3	4	5

Please mark one box for each scale.

1. The symptoms have disrupted your work*/school work:

Not at all	Mildly			Moderately			Markedly			Extremely
0	1	2	3	4	5	6	7	8	9	10

I have not worked/studied at all during the past week for reasons unrelated to the disorder.
*Work includes paid, unpaid volunteer work or training.

2. The symptoms have disrupted your social life/leisure activities:

Not at all	Mildly			Moderately			Markedly			Extremely
0	1	2	3	4	5	6	7	8	9	10

3. The symptoms have disrupted your family life/home responsibilities:

Not at all	Mildly			Moderately			Markedly			Extremely
0	1	2	3	4	5	6	7	8	9	10

Days lost
On how many days in the **last week** did your symptoms cause you to miss school or work or leave you unable to carry out your normal daily responsibilities? 0 1 2 3 4 5 6 7

Days unproductive
On how many days in the **last week** did you feel so impaired by your symptoms that even though you went to school or work, your productivity was reduced? 0 1 2 3 4 5 6 7

Feelings and faces study

	Never or very rarely true →				Almost always or always true
1. I am often confused about what emotion I am feeling.	1	2	3	4	5
2. I have physical sensations that even doctors don't understand.	1	2	3	4	5
3. When I am upset I don't know if I am sad, frightened, or angry.	1	2	3	4	5
4. I am often puzzled by sensations in my body.	1	2	3	4	5
5. I have feelings that I can't quite identify.	1	2	3	4	5
6. I don't know what's going on inside me.	1	2	3	4	5
7. I often don't know why I am angry.	1	2	3	4	5
8. It is difficult for me to find the right words for my feelings.	1	2	3	4	5
9. I am able to describe my feelings easily.	1	2	3	4	5
10. I find it hard to describe how I feel about people.	1	2	3	4	5
11. People tell me to describe my feelings more.	1	2	3	4	5
12. It is difficult for me to reveal my innermost feelings even to close friends.	1	2	3	4	5
13. I prefer to analyze problems rather than just describe them.	1	2	3	4	5
14. I prefer to just let things happen rather than to understand why they turned out that way.	1	2	3	4	5
15. Being in touch with emotions is essential.	1	2	3	4	5
16. I prefer talking to people about their daily activities rather than their feelings.	1	2	3	4	5
17. I prefer to watch "light" entertainment shows rather than psychological dramas.	1	2	3	4	5
18. I can feel close to someone, even in moments of silence.	1	2	3	4	5
19. I find examination of my feelings useful in solving personal problems.	1	2	3	4	5
20. Looking for hidden meanings in movies or plays distracts from their enjoyment.	1	2	3	4	5

S3 Text. Questionnaire Pack

Feelings and faces study

Attached is a list of problems and complaints that people have. Please read each one carefully. After you've done so, please fill in the number (0 to 4, see below) which best describes how much that problem has bothered or distressed you during the past four weeks including today. Choose only one number of each problem and do not skip any items. If you change your mind, cross out your first answer and write a new one. All questionnaires will be treated confidentially!

0= not at all; 1=a little bit; 2= moderately; 3= quite a bit; 4= extremely

How much will you bothered or distressed over the past four weeks by:

1. Nervousness or shakiness inside		28. Feeling afraid to travel on buses, subways, or trains.	
2. Faintness or dizziness		29. Trouble getting your breath	
3. The idea that someone else can control your thoughts		30. Hot or cold spells	
4. Feeling others are to blame the most of your troubles		31. Having to avoid certain things, places, or activities because they frighten you	
5. Trouble remembering things		32. Your mind going blank	
6. Feeling easily annoyed or irritated		33. Numbness or tingling in parts of your body	
7. Pains in heart or chest		34. The idea that you should be punished for your sins	
8. Feeling afraid in open spaces or on the street		35. Feeling hopeless about the future	
9. Thoughts of ending your life		36. Trouble concentrating	
10. Feeling that most people cannot be trusted		37. Feeling weak in parts of your body	
11. Poor appetite		38. Feeling tense or keyed up	
12. Suddenly scared for no reason		39. Thoughts of death or dying	
13. Temper outbursts that you could not control		40. Having urges to beat, injure, or harm someone	
14. Feeling lonely even when you are with people		41. Having urges to break or smash things	
15. Feeling blocked in getting things done		42. Feeling very self-conscious with others	
16. Feeling lonely		43. Feeling uneasy in crowds, such as shopping or at a movie	
17. Feeling blue		44. Never feeling close to another person	
18. Feeling no interest in things		45. Spells of terror or panic	
19. Feeling fearful		46. Getting into frequent arguments	
20. Your feelings being easily hurt		47. Feeling nervous when you are left alone	
21. Feeling the people are unfriendly or dislike you		48. Others not giving you proper credit your achievements	
22. Feeling inferior to others		49. Feeling so restless you couldn't sit still	
23. Nausea or upset stomach		50. Feelings of worthlessness	
24. Feeling that you are watched or talked about by others		51. Feeling that people will take advantage of you if you let them	
25. Trouble falling asleep		52. Feelings of guilt	
26. Having to check and double-check what you do		53. The idea that something is wrong with your mind	
27. Difficulty making decisions			

S3 Text. Questionnaire Pack

Feelings and faces study

	False/ Not at all true	Slightly true	Mainly true	Very true
1. My mood can shift quite suddenly	1	2	3	4
2. My attitude about myself changes a lot	1	2	3	4
3. My relationships have been stormy	1	2	3	4
4. My moods get quite intense	1	2	3	4
5. Sometimes I feel terribly empty inside	1	2	3	4
6. I want to let certain people know how much they've hurt me	1	2	3	4
7. My mood is very steady	1	2	3	4
8. I worry a lot about other people leaving me	1	2	3	4
9. People once close to me have let me down	1	2	3	4
10. I have little control over my anger	1	2	3	4
11. I often wonder what I should do with my life	1	2	3	4
12. I rarely feel very lonely	1	2	3	4
13. I sometimes do things so impulsively that I get into trouble	1	2	3	4
14. I've always been a pretty happy person	1	2	3	4
15. I can't handle separation from those close to me very well	1	2	3	4
16. I've made some real mistakes in the people I've picked as friends	1	2	3	4
17. When I'm upset, I typically do something to hurt myself	1	2	3	4
18. I've had times when I was so mad I couldn't do enough to express all my anger	1	2	3	4
19. I don't get bored very easily	1	2	3	4
20. Once someone is my friend, we stay friends	1	2	3	4
21. I'm too impulsive for my own good	1	2	3	4
22. I spend money too easily	1	2	3	4
23. I'm a reckless person	1	2	3	4
24. I am careful about how I spend my money	1	2	3	4

S3 Text. Questionnaire Pack

Feelings and faces study

Set A

In each group below, carefully circle the word that is closest in meaning to the word in heavy type above the group. Make sure you circle one word only. If you don't know the answer, have a guess or move onto the next question. The first one has been done for you as an example. Work downwards through each column.

**1. Rage**<br>1. crease 4. love<br>2. invite 5. anger<br>3. rain 6. hoist	**13. Virile**<br>1. demanding 4. familiar<br>2. concise 5. manly<br>3. vulgar 6. barbarous	**25. Obdurate**<br>1. Formidable 4. permanent<br>2. hesitant 5. stubborn<br>3. exorbitant 6. obsolete
**2. Squabble**<br>1. saw 4. lift<br>2. bubble 5. photo<br>3. mould 6. quarrel	**14. Surmount**<br>1. mountain 4. overcome<br>2. conceded 5. descend<br>3. appease 6. snub	**26. Palliate**<br>1. regenerate 4. qualify<br>2. alleviate 5. imitate<br>3. stimulate 6. erase
**3. Connect**<br>1. join 4. field<br>2. lace 5. bean<br>3. flint 6. accident	**15. Sultry**<br>1. instinctive 4. solid<br>2. sulky 5. severe<br>3. trivial 6. muggy	**27. Adulate**<br>1. increase 4. waver<br>2. admire 5. prosper<br>3. flatter 6. inflate
**4. Provide**<br>1. Harmonise 4. divide<br>2. hurt 5. commit<br>3. annoy 6. supply	**16. Criterion**<br>1. superior 4. critic<br>2. certitude 5. standard<br>3. clarion 6. crisis	**28. Felicitous**<br>1. sincere 4. faithful<br>2. valedictory 5. altruistic<br>3. voracious 6. opportune
**5. Brag**<br>1. choose 4. boast<br>2. hope 5. stone<br>3. lag 6. jerk	**17. Latent**<br>1.delayed 4. discharged<br>2. potential 5. overburdened<br>3. ingenious 6. hostile	**29. Ambit**<br>1. talisman 4. confines<br>2. armature 5. arc<br>3. camber 6. ideal
**6. Shrivel**<br>1. linger 4. heed<br>2. volunteer 5. wither<br>3. shiver 6. haunt	**18. Dwindle**<br>1. swindle 4. pander<br>2. linger 5. wheeze<br>3. diminish 6. compare	**30. Recondite**<br>1. brilliant 4. effervescent<br>2. vindictive 5. abstruse<br>3. indifferent 6. wise
**7.Mingle**<br>1. interfere 4. press<br>2. mix 5. declare<br>3. gamble 6.remark	**19. Construe**<br>1. prophesy 4. interpret<br>2. contradict 5. collect<br>3. scatter 6. anneal	**31. Cachinnation**<br>1. guffaw 4. succour<br>2. conclave 5. conjunction<br>3. cunning 6. controversy
**8. Stance**<br>1. partition 4. fixed<br>2. glance 5. slope<br>3. position 6. grief	**20. Efface**<br>1. delete 4. rotate<br>2. disgust 5. mark<br>3. adjoin 6.ascend	**32. Exiguous**<br>1. exhausting 4. prodigious<br>2. indigenous 5. esoteric<br>3. scanty 6. expedient
**9. Verify**<br>1. dedicate 4. confirm<br>2. chastise 5. change<br>3. correct 6.purify	**21. Trumpery**<br>1. etiquette 4. heraldry<br>2. worthless 5. highest<br>3. amusement 6. final	**33. Putative**<br>1. punishable 4. computable<br>2. supposed 5. worthless<br>3. aggressive 6. reconcilable
**10. Formidable**<br>1. unexpired 4. ravishing<br>2. feasible 5. orderly<br>3. tremendous 6.rememberence	**22. Perpetrate**<br>1. appropriate 4. control<br>2. propitiate 5. deface<br>3. commit 6. pierce	**34. Manumit**<br>1. manufacture 4. liberate<br>2. enumerate 5. emanate<br>3. accomplish 6. permit
**11. Thrive**<br>1. think 4. try<br>2. thrash 5. reap<br>3. blame 6. flourish	**23. Glower**<br>1. scowl 4. shine<br>2. disguise 5. gloat<br>3. aerate 6. extinguish	
**12. Docile**<br>1. meek 4. passionate<br>2. dominant 5. homely<br>3. careless 6. dumb	**24. Sensual**<br>1. controversial 4. careful<br>2. necessary 5. crucial<br>3. rational 6. carnal	

Feelings and faces study

Set B

The first one has been done for you. Work downwards through each column.

**1. Malaria**<br>1. basement<br>2. theatre<br>3. ocean<br>4. fever (circled)<br>5. fruit<br>6. tune	**13. Immerse**<br>1. frequent<br>2. reverse<br>3. rise<br>4. hug<br>5. dip<br>6. show	**25. Temerity**<br>1. impermanence<br>2. nervousness<br>3. punctuality<br>4. rashness<br>5. stability<br>6. submissiveness
**2. Fascinated**<br>1. ill-treated<br>2. poisoned<br>3. frightened<br>4. modelled<br>5. charmed<br>6. copied	**14. Conciliate**<br>1. congregate<br>2. pacify<br>3. compress<br>4. reverse<br>5. radiate<br>6. strengthen	**26. Fecund**<br>1. esculent<br>2. profound<br>3. sublime<br>4. optative<br>5. prolific<br>6. salic
**3. Liberty**<br>1. freedom<br>2. rich<br>3. forest<br>4. worry<br>5. serviette<br>6. cheerful	**15. Envisage**<br>1. enfeeble<br>2. surround<br>3. activate<br>4. contemplate<br>5. estrange<br>6. regress	**27. Abnegate**<br>1. contradict<br>2. renounce<br>3. belie<br>4. decry<br>5. execute<br>6. assemble
**4. Stubborn**<br>1. steady<br>2. obstinate<br>3. orderly<br>4. hopeful<br>5. hollow<br>6. slack	**16. Amulet**<br>1. cameo<br>2. flirtation<br>3. charm<br>4. jacket<br>5. crest<br>6. savoury	**28. Traduce**<br>1. challenge<br>2. suspend<br>3. misrepresent<br>4. attenuate<br>5. establish<br>6. conclude
**5. Precise**<br>1. natural<br>2. faulty<br>3. stupid<br>4. exact<br>5. grand<br>6. small	**17. Garrulous**<br>1. talkative<br>2. massive<br>3. ridiculous<br>4. daring<br>5. ugly<br>6. fast	**29. Vagary**<br>1. vagabond<br>2. obscurity<br>3. evasion<br>4. caprice<br>5. vulgarity<br>6. fallacy
**6. Resemblance**<br>1. memory<br>2. assemble<br>3. attendance<br>4. fondness<br>5. repose<br>6. likeness	**18. Libertine**<br>1. profligate<br>2. farrago<br>3. regicide<br>4. rescuer<br>5. canard<br>6. missionary	**30. Specious**<br>1. fallacious<br>2. palatial<br>3. nutritious<br>4. coeval<br>5. typical<br>6. flexible
**7. Anonymous**<br>1. applicable<br>2. insulting<br>3. nameless<br>4. magnificent<br>5. fictitious<br>6.untrue	**19. Bombastic**<br>1. democratic<br>2. bickering<br>3. destructive<br>4. anxious<br>5. cautious<br>6. pompous	**31. Sedulous**<br>1. rebellious<br>2. complaisant<br>3. seductive<br>4. dilatory<br>5. diligent<br>6. credulous
**8. Elevate**<br>1. raise<br>2. revolve<br>3. waver<br>4. move<br>5. work<br>6.disperse	**20. Levity**<br>1. parsimony<br>2. salutary<br>3. alacrity<br>4. frivolity<br>5. velleity<br>6. tariff	**32. Nugatory**<br>1. inimitable<br>2. sublime<br>3. numismatic<br>4. adamant<br>5. contrary<br>6. trifling
**9. Task**<br>1. horn<br>2. trap<br>3. problem<br>4. game<br>5. jail<br>6. job	**21. Whim**<br>1. complain<br>2. tonic<br>3. wind<br>4. noise<br>5. fancy<br>6. rush	**33. Adumbrate**<br>1. foreshadow<br>2. detect<br>3. elaborate<br>4. protect<br>5. eradicate<br>6. approach
**10. Courteous**<br>1. dreadful<br>2. polite<br>3. curtsey<br>4. proud<br>5. short<br>6. truthful	**22. Ruse**<br>1. limb<br>2. trick<br>3. colour<br>4. paste<br>5. burn<br>6. rude	**34. Minatory**<br>1. implacable<br>2. belittling<br>3. depository<br>4. diminutive<br>5. quiescent<br>6. threatening
**11. Prosper**<br>1. imagine<br>2. succeed<br>3. punish<br>4. propose<br>5. beseech<br>6. trespass	**23. Recumbent**<br>1. fugitive<br>2. unwieldy<br>3.penitent<br>4. cumbersome<br>5. repelling<br>6. reclining	
**12. Lavish**<br>1. unaccountable<br>2. romantic<br>3. extravagant<br>4. selfish<br>5. lawful<br>6. praise	**24. Querulous**<br>1. astringent<br>2. petulant<br>3. inquiring<br>4. fearful<br>5. curious<br>6. spurious	

S3 Text. Questionnaire Pack

Feelings and faces study

For each set of eyes, choose and circle which word best describes what the person in the picture is thinking or feeling. You may feel that more than one word is applicable but please choose just one word, the word which you consider to be most suitable. Before making your choice, make sure that you have read all 4 words. You should try to do the task as quickly as possible.

Most people surprise themselves by how well they do in this test. Even if you think you don't have a clue, just choose the one that 'feels' right.

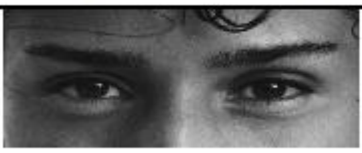<br>1. 1. playful 2. comforting 3. irritated 4. bored	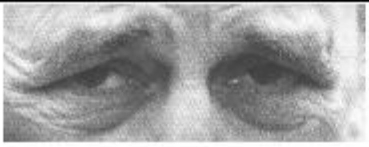<br>2. 1. terrified 2. upset 3. arrogant 4. annoyed
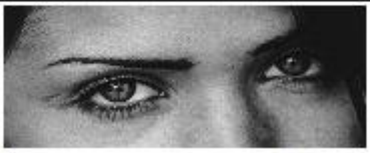<br>3. 1. joking 2. flustered 3. desire 4. convinced	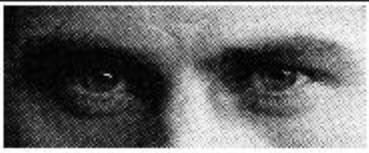<br>4. 1. joking 2. insisting 3. amused 4. relaxed
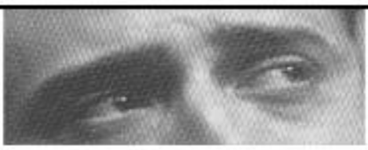<br>5. 1. irritated 2. sarcastic 3. worried 4. friendly	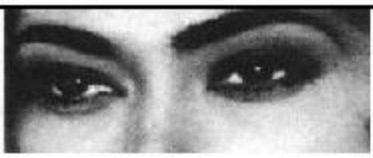<br>6. 1. aghast 2. fantasizing 3. impatient 4. alarmed

Feelings and faces study

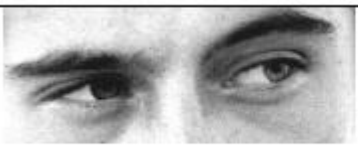

7. 1. apologetic 2. friendly 3. uneasy 4. dispirited

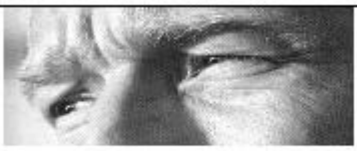

8. 1. despondent 2. relieved 3. shy 4. excited

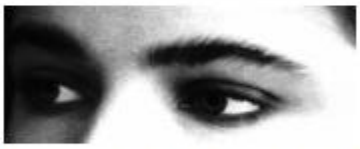

9. 1. annoyed 2. hostile 3. horrified 4. preoccupied

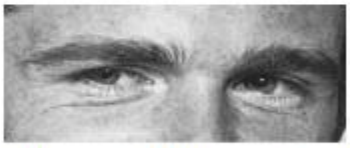

10. 1. cautious 2. insisting 3. bored 4. aghast

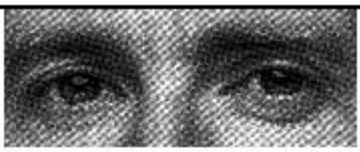

11. 1. terrified 2. amused 3. regretful 4. flirtatious

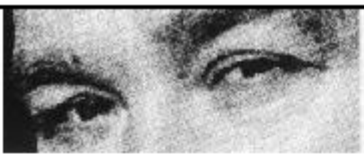

12. 1. indifferent 2. embarrassed 3. sceptical 4. dispirited

Feelings and faces study

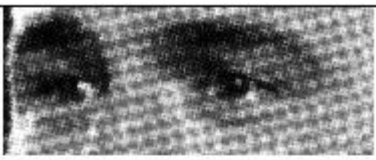

13. 1. decisive 2. anticipating 3. threatening 4. shy

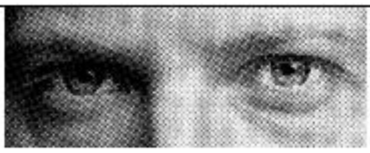

14. 1. irritated 2. disappointed 3. depressed 4. accusing

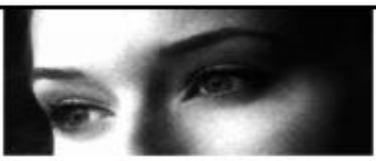

15. 1. contemplative 2. flustered 3. encouraging 4. amused

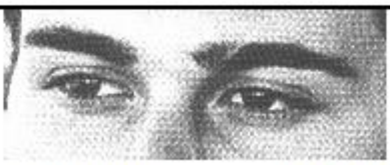

16. 1. irritated 2. thoughtful 3. encouraging 4. sympathetic

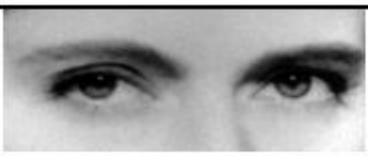

17. 1. doubtful 2. affectionate 3. playful 4. aghast

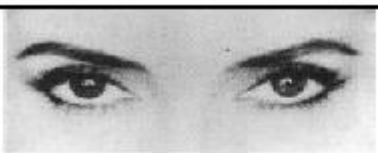

18. 1. decisive 2. amused 3. aghast 4. bored

S3 Text. Questionnaire Pack

Feelings and faces study

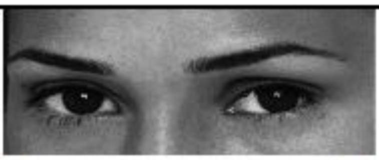

19. 1. arrogant 2. grateful 3. sarcastic 4. tentative

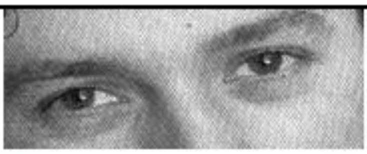

20. 1. dominant 2. friendly 3. guilty 4. horrified

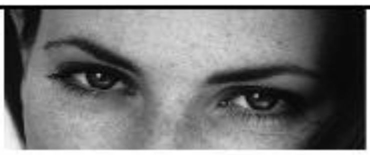

21. 1. embarrassed 2. fantasizing 3. confused 4. panicked

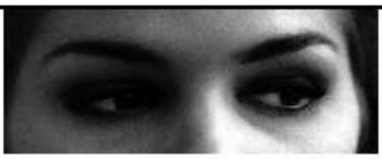

22. 1. preoccupied 2. grateful 3. insisting 4. imploring

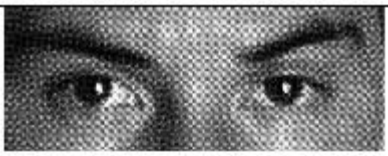

23. 1. contented 2. apologetic 3. defiant 4. curious

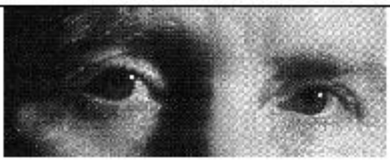

24. 1. pensive 2. irritated 3. excited 4. hostile

S3 Text. Questionnaire Pack

Feelings and faces study

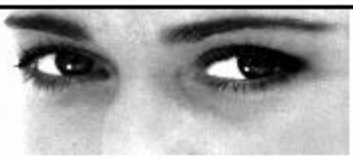 25. 1. panicked 2. incredulous 3. despondent 4. interested	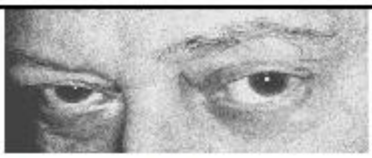 26. 1. alarmed 2. shy 3. hostile 4. anxious
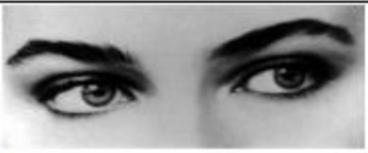 27. 1. joking 2. cautious 3. arrogant 4. reassuring	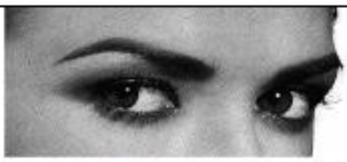 28. 1. interested 2. joking 3. affectionate 4. contented
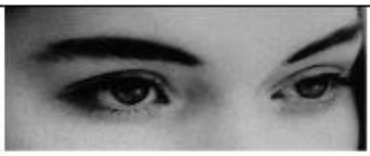 29. 1. impatient 2. aghast 3. irritated 4. reflective	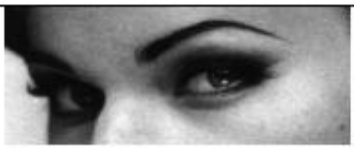 30. 1. grateful 2. flirtatious 3. hostile 4. disappointed

S3 Text. Questionnaire Pack

Feelings and faces study

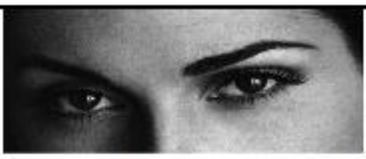

31. 1. ashamed 2. confident 3. joking 4. dispirited

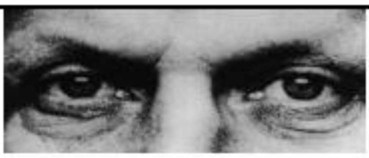

32. 1. serious 2. ashamed 3. bewildered 4. alarmed

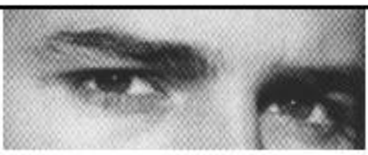

33. 1. embarrassed 2. guilty 3. fantasizing 4. concerned

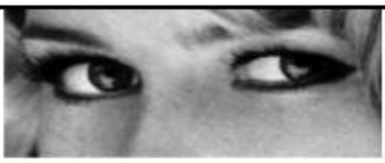

34. 1. aghast 2. baffled 3. distrustful 4. terrified

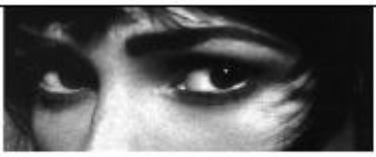

35. 1. puzzled 2. nervous 3. insisting 4. contemplative

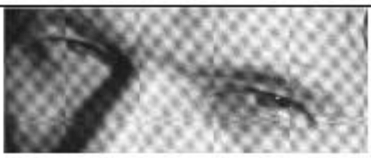

36. 1. ashamed 2. nervous 3. suspicious 4. indecisive

Feelings and faces study

	1 Not true	2	3	4 Some -what	5	6	7 Very true
1. I never cover up my mistakes.	1	2	3	4	5	6	7
2. There have been occasions when I have taken advantage of someone.	1	2	3	4	5	6	7
3. I always obey laws, even if I'm unlikely to get caught.	1	2	3	4	5	6	7
4. I have said something bad about a friend behind his/her back.	1	2	3	4	5	6	7
5. When I hear people talking privately, I avoid listening.	1	2	3	4	5	6	7
6. I have received too much change from a salesperson without telling him or her.	1	2	3	4	5	6	7
7. When I was young I sometimes stole things.	1	2	3	4	5	6	7
8. I have never dropped litter on the street.	1	2	3	4	5	6	7
9. I never look at sexy books or magazines.	1	2	3	4	5	6	7
10. I have done things that I don't tell other people about.	1	2	3	4	5	6	7
11. I have pretended to be sick to avoid work or school.	1	2	3	4	5	6	7
12. I don't gossip about other people's business.	1	2	3	4	5	6	7

Finally, please tick the following box *only* if you are a psychologist or psychotherapist with a specialist interest in 'mentalization'. ☐

Many thanks for taking the time to complete this questionnaire.
