# Supplementary material for: ‘Rich’ and ‘poor’ in mentalizing: Do expert mentalizers exist?
Source: PLoS One. 2021 Oct 25;16(10):e0259030. doi: 10.1371/journal.pone.0259030 (PMC8544847; doi:10.1371/journal.pone.0259030)
Supplement: S1 Table — (PDF) [file pone.0259030.s008.pdf]

S1_Table. Demographics

Variable	Category	BPD		Controls		Psych Therapists	
		M	*SD*	*M*	*SD*	*M*	*SD*
Age		34.65	9.911	34.85	10.360	33.38	9.618
		N	%	*N*	%	*N*	%
Gender	Female	30	78.9	28	84.8	39	97.5
	Male	8	21.1	5	15.2	1	2.5
Ethnicity	White British	30	78.9	22	66.7	30	75
	Any other White	4	10.5	6	18.2	8	20
	Other	3	7.8	5	15.1	2	5
Long term relationship?	Yes	10	26.3	15	45.5	30	75
	No	27	71.1	17	51.5	9	22.5
Employment status:	Employed	8	21.1	22	66.7	37	92.5
	Self-employed	1	2.6	8	24.2	1	2.5
	Unemployed	23	60.5	1	3.1	0	0
	Studying	3	7.9	0	0	0	0
	Retired	3	7.9	0	0	0	0
Occupation:	Modern professional	9	23.7	18	54.5	40	100
	Clerical and intermediate	9	23.7	2	6.1	-	-
	Senior managers or administrators	3	7.9	4	12.1	-	-
	Technical and craft	4	10.5	0	0	-	-
	Semi-routine manual and service	7	18.4	1	3	-	-
	Routine manual and service	1	2.6	0	0	-	-
	Middle or junior managers	1	2.6	2	6.1	-	-
	Traditional professional	2	5.2	6	18.2	-	-

Appendix 3: Demographics continued

Variable	Category	BPD		Controls		Psych Therapists	
		N	%	*N*	%	*N*	%
Education:	To age 16	11	28.9	1	3	-	-
	To age 18	9	23.7	4	12.1	-	-
	Vocational qualification	7	18.4	2	6.1	-	-
	University degree	9	23.7	15	45.5	-	-
	Post graduate degree	1	2.6	10	30.3	2	5
	Doctoral degree	1	2.6	1	3	38	95

NB: Figures exclude missing values
