# Supplementary material for: ‘Rich’ and ‘poor’ in mentalizing: Do expert mentalizers exist?
Source: PLoS One. 2021 Oct 25;16(10):e0259030. doi: 10.1371/journal.pone.0259030 (PMC8544847; doi:10.1371/journal.pone.0259030)
Supplement: S2 Table — (PDF) [file pone.0259030.s009.pdf]

S2 Table. Relationships between potential confounds and outcome variables

	Test statistic	RFQ18	RFQ-self	RFQ-other	TAS	PTS(SQRT)
**Gender**	*t*-test	-.71	-1.18	-.07	1.40	-.49
**Age**	Pearson's *r*	-.089	-.084	-.07	.06	-.01
**Ethnicity**	ANOVA *F*	1.50	1.13	1.51	.99	.29
**Relationship status**	*t*-test	1.59	2.49	.28	-3.44**	3.49**
**Employment stat EMP3**	ANOVA *F*	13.44[+]***	18.55**	4.36[+]	34.05**	9.60**
**Occupation OCCUP5**	ANOVA *F*	2.92*[+]	6.97**	.89[+]	12.24**	9.82**
**Years in education**	ANOVA *F*	7.78*[+]	14.21*	2.42[+]	12.48*[+]	4.52*
**MHV**	Pearson's *r*	.046	.14	-.07	-.24*	.27*
**BSI12(SQRT)**	Pearson's *r*	-.45**	-.63**	-.160	.82**	-.51**
**Months in therapy**	Pearson's *r*	0.00	-.06	.06	.19	-.09
**IMS**	Pearson's *r*	-.18	-.07	-.23*	-.18	.28**

BSI12 = Brief Symptom Inventory Anxiety & Depression subscales; **MHV**= Mill Hill Vocabulary Scale; **IMS**=Impression management Scale; **EMP3:** Employment status after collapsing six categories into three; **OCCUP(5)**=Occupation after collapsing eight categories into five; **SQRT**=after square root transformation.

* = sig $p < 0.05$
** = sig $p < 0.01$
[+] = Equality of variances could not be assumed. Games-Howell test of significance used (Field, 2005).
