# Supplementary material for: ‘Rich’ and ‘poor’ in mentalizing: Do expert mentalizers exist?
Source: PLoS One. 2021 Oct 25;16(10):e0259030. doi: 10.1371/journal.pone.0259030 (PMC8544847; doi:10.1371/journal.pone.0259030)
Supplement: S3 Table — (PDF) [file pone.0259030.s010.pdf]

S3 Table. Descriptive statistics for outcome measures

Variable	Total sample			BPD			Controls			Psychological Therapists		
	M	*SD*	*N*	*M*	*SD*	*N*	*M*	*SD*	*N*	*M*	*SD*	*N*
1. **RFQ18**	73.32	9.76	110	59.91	17.80	37	76.64	12.56	33	83.07	10.21	40
2. **RFQ-self**	35.72	16.98	110	25.62	7.86	37	38.24	5.98	33	42.97	4.87	40
3. **RFQ-other**	37.60	9.94	110	34.19	12.81	37	38.39	8.71	33	40.10	6.69	40
4. **TAS**	45.33	14.44	110	61.95	9.73	38	36.79	7.56	33	36.36	5.96	39
5. **PTS (SQRT)**	2.77	.85	110	2.17	.84	38	3.05	.71	33	3.12	.62	39
6. **IMS**	3.47	2.53	69	3.32	2.64	26	4.22	2.88	19	3.00	2.01	40
7. **BSI12(SQRT)**	3.52	1.85	109	5.57	.98	37	2.47	1.32	32	2.39	.96	39
8. **MHV**	64.51	8.95	109	61.27	10.05	38	67.42	7.54	32	65.71	7.90	24
9. **Therapy received (months)**	19.85	43.57	106	31.00	45.74	34	9.61	31.89	33	18.79	48.64	39
10. **PAI(BOR)**	28.46	18.84	111	51.84	9.91	38	17.58	8.60	33	15.22	5.89	40

TAS=Toronto Alexithymia Scale; **PTS**=Perspective Taking Subscale; **IMS**=Impression management Scale; **BSI12** = Brief Symptom Inventory Anxiety & Depression subscales; **MHV**= Mill Hill Vocabulary Scale; **SQRT**=after square root transformation
