# Supplementary material for: ‘Rich’ and ‘poor’ in mentalizing: Do expert mentalizers exist?
Source: PLoS One. 2021 Oct 25;16(10):e0259030. doi: 10.1371/journal.pone.0259030 (PMC8544847; doi:10.1371/journal.pone.0259030)
Supplement: S4 Table — (PDF) [file pone.0259030.s011.pdf]

S4 Table. Correlation matrix for continuous variables

	RFQ18	RFQ-self	RFQ-other	TAS	PTS (SQRT)	PAI-BOR	BSI12 (SQRT)	Months in therapy	MHV	IMS	Age
**RFQ18**	1.00	.86**	.87**	-.47**	.32**	-.56**	-.46**	.00	.05	-.18	-.09
**RFQ-self**	.86**	1.00	.49**	-.67**	.45**	-.71**	-.63**	-.07	.14	-.07	-.08
**RFQ-other**	.87**	.49**	1.00	-.14	.12	-.25**	-.16	.06	-.07	-.23*	-.07
**TAS**	-.47**	-.67**	-.14	1.00	-.52**	.85**	.83**	.19	-.24*	-.18	.06
**PTS(SQRT)**	.32**	.44**	.12	-.52**	1.00	-.59**	-.51**	-.09	.27*	.29**	-.01
**PAI-BOR**	-.55**	-.71**	-.25**	.85**	-.59**	1.00	.87**	.21*	-.31**	-.15	.05
**BSI12 (SQRT)**	-.46**	-.63**	-.16	.83**	-.51**	.87**	1.00	.18	-.19	-.20*	-.08
**Months in therapy**	.00	-.07	.06	.19	-.09	.21*	.18	1.00	.08	-.06	.41**
**MHV**	.046	.14	-.07	-.24*	.27*	-.31*	-.19	.08	1.00	.03	.44**
**IMS**	-.18	-.07	-.23*	-.18	.29**	-.15	-.20*	-.06	.03	1.00	.03
**Age**	-.09	-.08	-.07	.06	-.01	.05	.08	.41**	.44**	.03	1.00

TAS=Toronto Alexithymia Scale; **PTS**=Perspective Taking Subscale; **PAI-BOR** = Personality Assessment Inventory- Borderline subscale; **BSI12** = Brief Symptom Inventory Anxiety & Depression subscales; **MHV**= Mill Hill Vocabulary Scale; **IMS**=Impression management Scale; **SQRT**=after square root transformation.

* = sig $p < 0.05$
** = sig $p < 0.01$
