# Supplementary material for: ‘Rich’ and ‘poor’ in mentalizing: Do expert mentalizers exist?
Source: PLoS One. 2021 Oct 25;16(10):e0259030. doi: 10.1371/journal.pone.0259030 (PMC8544847; doi:10.1371/journal.pone.0259030)
Supplement: S5 Table — (PDF) [file pone.0259030.s012.pdf]

S5 Table. Normality of untransformed variables

Untransformed variables		Skewness			Kurtosis		
		BPD	Controls	Psychological Therapists	BPD	Controls	Psychological Therapists
**RFQ-18**	N	37	33	40	37	33	40
	Statistic	-.438	-.539	-.291	-.392	-.273	-.313
	St. Error	.388	.409	.374	.759	-.738	.733
	Z-score	1.13	1.32	.78	.52	0.34	.43
**RFQ-Self**	N	37	33	40	37	33	40
	Statistic	.325	-.698	-.734	-.674	-.129	-.081
	St. Error	.388	.409	.374	.759	.798	.733
	Z-score	.84	1.71	1.96	.89	.16	.11
**RFQ-Other**	N	37	33	40	37	33	40
	Statistic	-.732	-.335	.311	-.867	-.154	.005
	St. Error	.388	.409	.374	.759	.798	.733
	Z-score	1.89	0.82	0.83	1.14	0.19	0.007

Criteria for normality: *Z*-Score > 1.96 on either skewness or kurtosis considered non-normal for N<100 (Fife-Shaw, 2011)

Untransformed variables		Skewness			Kurtosis		
		BPD	Controls	Psychological Therapists	BPD	Controls	Psychological Therapists
**TAS**	N	38	33	39	38	33	39
	Statistic	.020	.359	-.091	-.373	-.396	-.340
	St. Error	.383	.409	.378	.750	.798	.741
	Z-score	.05	.88	.24	.50	.50	.46
**PTS**	N	38	33	39	38	33	39
	Statistic	-.313	-1.082	-.058	-.505	1.824	-.188
	St. Error	.383	.409	.378	.750	.798	.741
	Z-score	.82	2.64	.15	.67	2.29	.25

TAS=Toronto Alexithymia Scale; **PTS**=Perspective Taking Subscale.

Criteria for normality: *Z*-Score > 1.96 on either skewness or kurtosis considered non-normal for N < 100 (Fife-Shaw, 2011).

Untransformed variables		Skewness			Kurtosis		
		BPD	Controls	Psychological Therapists	BPD	Controls	Psychological Therapists
**BSI12**	N	38	33	39	38	32	39
	Statistic	-.315	1.956	1.976	-.472	4.443	4.499
	St. Error	.383	.414	.378	.750	.809	.741
	Z-score	.82	4.72	5.23	.63	5.49	6.07
**MHV**	N	26	19	24	26	19	24
	Statistic	.083	.183	.396	.003	.062	-.670
	St. Error	.456	.524	.472	.887	1.014	.918
	Z-score	.18	.35	.84	.003	.06	.73
**IMS**	N	38	33	40	38	33	40
	Statistic	.667	.888	.556	-.619	.243	.498
	St. Error	.383	.409	.374	.750	.798	.733
	Z-score	1.74	2.17[1]	1.49	.82	.30	.70

BSI12 =Brief Symptom Inventory (Anxiety and Depression subscales); **MHV**=Mill Hill Vocabulary Scale;
IMS= Impression Management Subscale.Criteria for normality: *Z*-Score > 1.96 on either skewness or kurtosis considered non-normal for N<100 (Fife-Shaw, 2011).[1]

[1]Marginal normality not improved by square root, Logarithmic or reciprocal transformation. Retained for parametric analyses.
