# Supplementary material for: ‘Rich’ and ‘poor’ in mentalizing: Do expert mentalizers exist?
Source: PLoS One. 2021 Oct 25;16(10):e0259030. doi: 10.1371/journal.pone.0259030 (PMC8544847; doi:10.1371/journal.pone.0259030)
Supplement: S6 Table — (PDF) [file pone.0259030.s013.pdf]

S6 Table. Normality of transformed variables

Transformed variables		Skewness			Kurtosis		
		BPD	Controls	Psychological Therapists	BPD	Controls	Psychological Therapists
**PTS (SQRT)**	N	38	33	39	38	33	39
	Statistic	.893	.050	.546	.656	1.465	.225
	St. Error	.383	.409	.378	.750	.798	.741
	Z-score	2.33[1]	0.12	1.44	.87	1.84	.30
**BSI12(SQRT)**	N	38	32	39	38	32	39
	Statistic	-.816	.960	1.058	.772	.502	1.023
	St. Error	.383	.414	.378	.750	.809	.741
	Z-score	2.13[1]	2.32	2.80	1.03	0.62	1.38

PTS=Perspective Taking Subscale; **BSI12** =Brief Symptom Inventory (Anxiety and Depression subscales).
SQRT=Square root transformation**.**

Criteria for normality: *Z*-Score > 1.96 on either skewness or kurtosis considered non-normal for N<100 (Fife-Shaw, 2011)

[1] Marginal normality but transformed variable retained for parametric analyses.
