# Supplementary material for: ‘Rich’ and ‘poor’ in mentalizing: Do expert mentalizers exist?
Source: PLoS One. 2021 Oct 25;16(10):e0259030. doi: 10.1371/journal.pone.0259030 (PMC8544847; doi:10.1371/journal.pone.0259030)
Supplement: S7 Table — (PDF) [file pone.0259030.s014.pdf]

S7 Table. Normality of residuals of main outcome measures

Outcome variables		Skewness	Kurtosis
**RFQ18**	N	110	110
	Statistic	-.479	.171
	St. Error	.230	.457
	Z-score	2.08	.37
**RFQ-self**	N	110	110
	Statistic	-.080	.815
	St. Error	.230	.457
	Z-score	.35	1.78
**RFQ-other**	N	110	110
	Statistic	-.613	-.002
	St. Error	.230	.457
	Z-score	2.66	.004
**TAS**	N	110	110
	Statistic	.006	-.354
	St. Error	.230	.457
	Z-score	.026	.77
**PTS(SQRT)**	N	110	110
	Statistic	.552	.608
	St. Error	.230	.457
	Z-score	2.40	1.3

TAS=Toronto Alexithymia Scale;
PTS=Perspective Taking Subscale;
SQRT=Square root transformation**.**

Criteria for normality: Z-Score > 3.29 on either skewness or kurtosis considered non-normal for N<200 (Fife-Shaw, 2011).
